# Digital Physical Exercise Interventions for Cognitive Functions in Older Adults: Systematic Review and Bayesian Network Meta-Analysis of Randomized Controlled Trials

**DOI:** 10.2196/92764

**Published:** 2026-08-18

**Authors:** Qing Hu, Siyi Sun, Jingru Zhu, Xiaoke Zhong, Shengyu Dai, Changhao Jiang

**Affiliations:** 1 The Center of Neuroscience and Sports, Capital University of Physical Education and Sports, No. 11, West Section of North Third Ring Road, Haidian District, Beijing, 100191, China, 86 13701287984; 2 School of Kinesiology and Health, Capital University of Physical Education and Sports, Beijing, China; 3 School of Physical Education and Sport Science, Fujian Normal University, Fuzhou, Fujian, China

**Keywords:** Bayesian network meta-analysis, digital physical exercise, cognitive function, older adults, exergames, virtual reality, remote exercise, systematic review

## Abstract

**Background:**

Cognitive decline in older adults imposes a major global burden, with physical inactivity a leading modifiable risk factor for dementia. Digital physical exercise interventions offer scalable alternatives to traditional programs, but comparative effectiveness across cognitive domains remains unclear.

**Objective:**

The aim of the study is to compare and rank 4 digital physical exercise interventions—immersive virtual reality exercise (IVR_E), nonimmersive exergame (NI_ExG), remote exercise (RE), and virtual reality exercise combined with cognitive training (VR_EC)—against routine intervention (RI) or nonintervention (NI) on global cognition, executive function, and memory function in older adults aged ≥60 years.

**Methods:**

Eligible studies were English-language randomized controlled trials evaluating digital physical exercise on any untrained cognitive outcome in older adults. In total, 6 databases (PubMed, Embase, Web of Science, CENTRAL, PsycINFO, and CINAHL) and 2 trial registries were searched from January 2010 to April 2026; reference lists were screened. Screening, data extraction, and risk-of-bias assessment were conducted independently in duplicate using the Cochrane tool. Bayesian network meta-analyses were performed in R, reporting standardized mean differences (SMDs) with 95% CIs and prediction intervals (PIs); surface under the cumulative ranking curve (SUCRA) ranked interventions, and CINeMA (Confidence in Network Meta-Analysis) assessed certainty of evidence, following PRISMA (Preferred Reporting Items for Systematic Reviews and Meta-Analyses) 2020 and PRISMA-NMA (Preferred Reporting Items for Systematic Reviews and Meta-Analyses extension for Network Meta-Analyses).

**Results:**

In total, 51 randomized controlled trials (3673 participants) were included. NI_ExG significantly outperformed both NI (SMD 0.51, 95% CI 0.26-0.78; PI −0.03 to 1.07) and RI (SMD 0.32, 95% CI 0.12-0.53; PI −0.20 to 0.86) for global cognition (30 studies); IVR_E also outperformed NI (SMD 0.74, 95% CI 0.11-1.36; PI −0.04 to 1.53) and ranked highest by SUCRA. For executive function (33 studies), only NI_ExG versus NI was significant (SMD 0.39, 95% CI 0.04-0.76; PI −0.48 to 1.29). For memory function (20 studies), RE was significantly superior to both NI (SMD 1.30, 95% CI 0.15-2.44; PI −0.05 to 2.70) and RI (SMD 1.22, 95% CI 0.15-2.28; PI 0.07-2.56), with its PI also excluding the null; VR_EC was significantly inferior to RE (SMD −1.54, 95% CI −2.89 to −0.24; PI −3.09 to −0.07). Cumulative training ≥1000 minutes was associated with more stable memory benefit. Certainty was very low for most comparisons, downgraded for unclear allocation concealment, heterogeneity, and suspected reporting bias in executive function.

**Conclusions:**

This Bayesian network meta-analysis compares 4 digital physical exercise categories against active and passive controls across 3 cognitive domains. Comparative effectiveness was domain-specific: NI_ExG most consistently benefited global cognition and executive function; RE produced the only statistically robust memory improvement; and IVR_E achieved the highest rankings but requires confirmatory trials. The scalability of RE and NI_ExG makes them practical for older adults in rural and resource-limited settings, providing an evidence base to inform clinical guidelines and digital health investment.

## Introduction

### Rationale

Research indicates that cognitive functioning declines with age [1]. Global estimates from the Global Burden of Disease Study 2021 suggest that approximately 49.1 million adults aged 65 years and older were living with Alzheimer disease and other dementias in 2021, and this number is projected to reach approximately 191 million (95% uncertainty interval 52‐330 million) by 2050 [2]. The rising prevalence of cognitive decline and dementia places an immense financial and operational burden on health care systems, creating an urgent need for scalable and cost-effective preventive strategies [3]. Without timely and appropriate interventions, cognitive impairment can irreversibly progress to neurodegenerative diseases, including mild cognitive impairment (MCI) and dementia [4,5]. The 2024 report of the Lancet standing Commission on dementia identified 14 modifiable risk factors across the life course, including physical inactivity, and estimated that addressing these factors could potentially prevent or delay up to 45% of dementia cases worldwide [6]. Among these risk factors, physical inactivity in later life has been associated with a significantly increased incidence of all-cause dementia (relative risk 1.39, 95% CI 1.16‐1.67) [3]. These findings highlight the substantial potential for prevention through behavioral interventions targeting physical activity. Numerous studies have shown that exercise effectively maintains, enhances, and even prevents cognitive decline in older adults, regardless of their health status or levels of cognitive impairment [7-9].

However, traditional face-to-face exercise interventions are costly due to the requirement for significant human resources [10]. Moreover, these interventions require participants to travel to designated training facilities, which poses a significant barrier for older adults living in rural or remote areas with limited transportation access, thereby restricting the reach and scalability of such programs. In light of these constraints, researchers have begun to explore more efficient and accessible intervention methods, leading to the emergence of digital physical exercise interventions [11,12]. In contrast to traditional methods, digital physical exercise interventions have the potential to reach large populations across diverse locations at a relatively low cost [13]. Beyond scalability, these technologies often leverage gamification elements (eg, real-time feedback, rewards, and immersion) to enhance motivation and adherence among older adults, addressing a common dropout issue in traditional exercise programs [11,12]. Existing research indicates that digital physical exercise interventions, including exergames, virtual reality (VR) exercise, and app-based exercise, significantly enhance cognitive function in older adults [14-16].

Among the various types of digital physical exercise interventions, exergames have been the most extensively studied. Recent experimental findings suggest that, compared to traditional exercise interventions of equivalent duration, exergames yield significantly greater improvements in executive function among both healthy older adults and older adults with cognitive impairment [17-19]. Research shows that exergames significantly enhance global cognition and executive function in older adults compared to routine care or daily life [20-22]. Although several studies have demonstrated that exergames significantly improve cognitive function compared to traditional exercise [17-19], other research suggests that their effects may not differ significantly from those of traditional interventions [23], and some studies have reported no significant improvements at all [24,25]. These discrepancies may be explained by factors such as older adults’ discomfort with VR devices, the risks inherent in working with this population, high dropout rates, and variations in intervention implementation.

Several systematic reviews and meta-analyses have examined the effects of specific types of digital physical exercise interventions on cognitive outcomes in older adults. Previous reviews have evaluated the effects of exergames on executive function [26] and on overall cognitive function in older adults with MCI or dementia [27,28]. Other reviews have examined the components of effective exergame-based training for cognitive improvement [29] and explored the cognitive benefits of exergames [30], as well as investigated the combined effects of VR and cognitive interventions on populations with MCI [31]. Additionally, several experimental studies have investigated the cognitive effects of online and app-based exercise in older adults [32-34]. Furthermore, network meta-analyses (NMAs) have compared the effects of different traditional exercise modalities, such as aerobic, resistance, and mind-body exercise, on cognitive function in older adults [35] and in older adults with cognitive impairment [8,36]. Most existing reviews on digital physical exercise interventions have primarily concentrated on promoting physical activity [12,13]. However, these reviews have notable limitations. First, most focused on a single type of digital intervention (eg, exergames only) rather than comparing across multiple digital modalities. Second, several were restricted to specific populations, such as those with MCI or dementia, limiting the generalizability of findings to the broader older adult population. Third, the NMAs that have compared exercise types have predominantly focused on traditional exercise modalities, without differentiating among the various forms of digital physical exercise. However, the comparative effectiveness of different types of digital physical exercise interventions—such as immersive virtual reality exercise (IVR_E), nonimmersive exergames (NI_ExG), remote exercise (RE), and virtual reality exercise combined with cognitive training (VR_EC)—has yet to be evaluated within a single network meta-analytic framework that simultaneously addresses multiple cognitive domains in older adults across the cognitive spectrum.

### Objectives

To address these gaps, this study aimed to conduct a Bayesian NMA of randomized controlled trials (RCTs) to systematically compare and rank the effectiveness of different digital physical exercise interventions on cognitive function in older adults. Specifically, we evaluated 4 types of digital physical exercise interventions—IVR_E, NI_ExGs, RE, and VR_EC—compared with routine interventions (RIs) or nonintervention (NI), on 3 cognitive outcomes: global cognition, executive function, and memory function, in older adults aged 60 years and older. By evaluating the comparative effectiveness of these interventions, this study aims to provide clinicians and policymakers with evidence-based recommendations on the most effective and feasible digital physical exercise strategies for cognitive health in aging populations.

## Methods

### Registration and Protocol

This study follows the PRISMA (Preferred Reporting Items for Systematic Reviews and Meta-Analyses) 2020 guidelines [37] and the PRISMA extension for NMA [38] and was prospectively registered in PROSPERO (CRD420251030142). The corresponding PRISMA checklist and PRISMA-NMA (Preferred Reporting Items for Systematic Reviews and Meta-Analyses extension for Network Meta-Analyses) checklist are provided in Checklist 1 and Checklist 2, respectively. The abstract was prepared in accordance with the PRISMA 2020 for Abstracts guidance, with the completed checklist provided in Checklist 3. The following amendments were made to the registered protocol during the conduct of the review: (1) the database search was updated on April 15, 2026, by rerunning the identical search strategies across all 6 databases to extend coverage to April 1, 2026; this update was not prespecified in the original protocol but was implemented to ensure currency of the evidence base. (2) Prediction intervals (PIs) were calculated for all pairwise comparisons from the NMA using the improved methods proposed by Noma et al [39], implemented via the *netmeta* package in R (R Foundation for Statistical Computing); this approach was not specified in the original protocol but was adopted to provide a more comprehensive estimate of the range of true effects expected in future similar studies. (3) Certainty of evidence was assessed using the CINeMA (Confidence in Network Meta-Analysis) framework, which was not specified in the original protocol; this addition was made to meet journal reporting standards and strengthen the transparency of evidence grading. No other deviations from the registered protocol were made.

### Eligibility Criteria

We included published, peer-reviewed RCTs that evaluated the effects of digital physical exercise interventions on cognitive functions in older adults, with no restriction on trial type or sample size. Eligible studies had to report at least 1 cognitive outcome not directly targeted by the intervention and to compare the digital exercise intervention with either cognitive training alone, physical training alone, or a passive control condition. Studies were ineligible if they did not measure any cognitive outcome or measured one but did not report the relevant results.

### Type of Studies

Eligible designs were RCTs, including parallel-group and crossover trials. Only full-text papers published in English were considered; we excluded conference abstracts, protocols, commentaries, and nonpeer-reviewed reports.

### Type of Participants

Included studies focused on older adults aged 60 years and older, encompassing both cognitively healthy individuals and those with subjective cognitive complaints, MCI, or dementia—populations previously examined in reviews of physical-exercise efficacy. Eligibility was determined by reviewing study inclusion criteria and baseline sample characteristics. The 60-year threshold follows the United Nations definition of older persons [40] and widely used criteria in aging research.

### Type of Interventions

We focused on interventions in which digital technology delivered all or most of the exercise content. These encompassed, but were not limited to, exergames (including video games, augmented reality, and VR), VR exercise, online exercise programs, and mobile apps. We excluded studies in which the digital component served only as a monitoring tool (eg, wearable activity trackers) without delivering exercise content.

### Type of Controls

We included studies comparing digital physical exercise with nondigital physical exercise, cognitive training, a sham intervention (eg, health education), or a passive control (eg, NI). For multiarm studies, all eligible control conditions were considered.

### Type of Outcomes

Outcomes were baseline-to-postintervention changes in cognitive measures not directly targeted by the intervention, either global or domain-specific. Three cognitive outcome domains were examined: (1) global cognition, assessed with composite instruments of overall cognitive ability (eg, Mini-Mental State Examination and Montreal Cognitive Assessment); (2) executive function, assessed with tasks of cognitive control, flexibility, or inhibition (eg, Trail Making Test Part B and Stroop Test); and (3) memory function, assessed with measures of encoding, storage, or retrieval (eg, digit span and verbal learning tests). Eligible cognitive measures were assessed using validated neuropsychological instruments. Studies that reported only trained cognitive outcomes (ie, tasks identical to the intervention content) were excluded. Detailed domain definitions and the full list of assessment tools appear under the Outcomes section and in Multimedia Appendix 1.

For synthesis, interventions were grouped into 4 categories (IVR_E, NI_ExG, RE, and VR_EC) and comparators into 2 (RI and NI), as detailed under the Other Variables section, corresponding directly to the comparisons specified in the Objectives section.

### Information Sources

We searched 6 electronic databases: PubMed (MEDLINE), Embase (via Embase.com), Web of Science Core Collection (SCI-EXPANDED and SSCI), the Cochrane CENTRAL (via Cochrane Library), PsycINFO (via EBSCOhost), and CINAHL Plus with Full Text (via EBSCOhost), each searched individually. The initial search (April 29, 2025) was updated on April 15, 2026, by rerunning identical strategies across all 6 databases; both covered January 1, 2010, to April 1, 2026. Records per database are reported in the PRISMA flow diagram. We also searched ClinicalTrials.gov and the World Health Organization International Clinical Trials Registry Platform registries for ongoing or recently completed trials and manually screened the reference lists of all included studies and relevant reviews. No additional online sources, conference proceedings, or gray literature were browsed, and study authors were not contacted. The search was reported per the PRISMA-S (PRISMA extension for Reporting Literature Searches in Systematic Reviews) extension [41], with the completed checklist provided in Checklist 4.

### Search Strategy

The strategy was built around 4 concept blocks—intervention, population, cognitive outcomes, and study design—using controlled vocabulary (MeSH, EMTREE, APA Thesaurus, and CINAHL Headings) and free-text synonyms with Boolean operators. The 2010 date restriction was applied because digital physical exercise interventions for older adults emerged as a distinct field around that time. Searches were limited to English-language publications because of translation resource constraints; no language restriction was applied in CENTRAL to minimize language bias. Validated RCT filters were used in each database: the Cochrane Highly Sensitive Search Strategy for PubMed [42], the Cochrane Embase RCT filter (2023 revision) [43], the Cochrane CINAHL filter [44], and an adapted PsycINFO filter; no design filter was applied to CENTRAL. Strategies were developed independently for this review and reviewed by all team members, but were not formally peer-reviewed using an instrument such as PRESS (Peer Review of Electronic Search Strategies). Full strategies, copied exactly as run, appear in Multimedia Appendix 2. The searches yielded 30,454 records across the 6 databases (PubMed: n=6842; Embase: n=9517; Web of Science: n=5963; CENTRAL: n=3286; PsycINFO: n=2714; CINAHL: n=1893), plus 239 from trial registries.

### Selection Process

Two reviewers (QH and SS) independently screened titles or abstracts and full-text reports, resolving disagreements by discussion and consulting a third reviewer (CJ) when needed. No automation tools were used. Duplicates were removed with EndNote (version 21; Clarivate), followed by manual verification.

### Data Collection Process

One reviewer (QH) extracted data from each report, and a second reviewer (SS) independently verified all extractions, with disagreements resolved by discussion and, when necessary, consultation with a third reviewer (CJ). No automation tools or figure-extraction software were used, and no data were sought from investigators. For multiple reports of the same dataset, data were taken from the most comprehensive report, supplemented by the others where necessary. Where means and SDs were not reported directly (eg, only medians and 95% CIs), we derived them using methods from the Cochrane Handbook for Systematic Reviews of Interventions [45].

### Data Items

#### Outcomes

The cognitive outcomes were global cognition, executive function, and memory function—the constructs most consistently shown to benefit from physical activity in older adults. Prior evidence indicates that physical activity improves cognition in older adults with MCI and dementia, with the most robust benefits for executive functioning and memory [46], and that fitness training has selective effects, with the largest gains for executive-control processes [47]. These domains represent distinct constructs—overall ability, higher-order control, and information retention—that together capture the breadth of exercise-related cognitive benefit: global cognition is a broad construct spanning multiple domains [36]; executive function coordinates and regulates cognitive processes during complex tasks [48,49]; and memory function concerns the storage and retrieval of information, one of the most intricate domains [50]. Because domains differ in sensitivity to exercise, all 3 were included [36]. For each domain, all compatible results in eligible studies were sought; where a study reported multiple measures within a domain, the most commonly used instrument was selected for comparability. Multimedia Appendix 1 lists the assessment tools used. Data were sought for outcomes measured at the immediate postintervention time point; follow-up assessments beyond the intervention period were not included.

#### Other Variables

We collected study characteristics (first author, publication year, country, sample size, mean age, sex ratio, and cognitive status) and intervention characteristics (type, duration, frequency, total training dose, and delivery mode). Missing or unclear information was treated as missing, with no assumptions about unreported data.

Interventions were categorized into 4 types by hardware platform and immersion level: IVR_E, using systems that isolate the user or create high immersion, such as immersive interactive walls (Cave Automatic Virtual Environment–like systems) or specialized 3D VR simulators (eg, head-mounted display); NI_ExG, console-based games on external screens (televisions or monitors) requiring gross motor movement via motion-tracking sensors (eg, Xbox Kinect and Nintendo Wii), handheld controllers, or pressure-sensitive step mats or platforms; RE, defined as home-based physical training delivered via telecommunication technologies, including videoconferencing platforms (eg, Zoom), tablet or smartphone apps, and online video guidance without interactive gamification elements; and VR_EC, which represents dual-task interventions explicitly integrating physical exercise (typically cycling or walking) with cognitive challenges (eg, memory games or spatial navigation tasks). Regarding comparison groups, we classified them as either RI, comprising active control groups receiving nondigital traditional physical exercise, isolated cognitive training (eg, board games), or health education; or NI, representing passive control groups maintaining their usual daily lifestyle or receiving usual care.

### Study Risk of Bias Assessment

Two reviewers (QH and SS) independently assessed risk of bias using the Cochrane Risk of Bias tool (version 1), per the Cochrane Handbook [45]. Because participants cannot be blinded in exercise trials, we evaluated the remaining 6 domains: random sequence generation, allocation concealment, blinding of outcome assessors, incomplete outcome data, selective reporting, and other sources. An overall judgment was assigned by the “worst domain” rule: “low risk” if all domains were low, “some concerns” if at least 1 was unclear but none high, and “high risk” if any domain was high. A third reviewer (CJ) resolved disagreements. No automation tools were used.

### Effect Measures

We calculated standardized mean differences (SMDs) and 95% CIs from pre- and postintervention data, using SMDs because studies used different instruments within the same domains. For outcome measures where a lower raw score indicated better performance (eg, completion time on the Trail Making Test), values were reverse-coded prior to SMD calculation so that positive SMD values consistently favor the intervention group. Effect sizes were interpreted as small (SMD<0.40), moderate (0.40‐0.70), or large (>0.70) per the Cochrane Handbook [45].

### Synthesis Methods

Studies were eligible for a synthesis (pairwise meta-analysis or NMA) if they provided enough data to compute an SMD and its SE for at least one domain, with the contributing set determined by data availability and the requirement of a connected comparison network. We confirmed no double counting and that each study contributed independent participants to each comparison. Where means and SDs were not reported directly (eg, only medians and 95% CIs), we derived them using Cochrane Handbook conversion methods [45].

Individual-study and synthesis results were presented using forest plots for pairwise meta-analyses, network plots, league tables of all pairwise comparisons from the Bayesian NMA, and surface under the cumulative ranking curve (SUCRA) rankings [51].

In line with the minimally informative priors of the Bayesian framework [52], we first performed conventional pairwise meta-analyses under a random-effects model. Pooled effects for each cognitive domain and the corresponding forest plots were computed in R (version 4.4.2) using the *meta* package, whereas comparison-specific and subgroup pairwise analyses were performed in Stata (version 15.0; StataCorp). For all pairwise random-effects meta-analyses, the pooled effects and their CIs were estimated using the Hartung-Knapp-Sidik-Jonkman (HKSJ) method, which estimates the weighted pooled average with greater precision and yields fewer false positives than conventional random-effects methods, as recommended by IntHout et al [53]. Because the HKSJ correction factor can fall below 1 when only a few studies with similar effects are pooled—producing CIs narrower than the conventional random-effects interval—the factor was truncated at 1 (the modified HKSJ approach) in such comparisons; comparisons informed by a single study were summarized without pooling. Heterogeneity was quantified with the *I*^2^ statistic (thresholds of 25%, 50%, and 75% for mild, moderate, and high, respectively) [38]. A random-effects model was likewise used for the NMA to account for between-study variance.

Before the NMA, 4 reviewers (QH, SS, JZ, and CJ) evaluated the transitivity assumption to confirm that clinical and methodological characteristics (populations, design, and end points) were sufficiently similar for valid indirect inference, and a network plot illustrated all relationships. We ran 4 independent Markov Chain Monte Carlo chains [54,55], each from a random state with 15,000 iterations and 5000 burn-in samples to ensure convergence [56]. All NMA used the *Gemtc* package (version 1.0‐2) in R (version 4.4.2). Interventions were ranked by SUCRA, with higher values indicating better ranking [51]. PIs were calculated for all pairwise comparisons to estimate the range of true effects expected in a future similar study [57], using the *netmeta* package in R with the improved method of Noma et al [39], which accounts for between-study heterogeneity variance (τ²) in addition to sampling error.

We used node-splitting to assess local consistency between direct and indirect estimates for each comparison. Given the heterogeneity inherent in digital interventions (eg, varying hardware and protocols), we did not exclude outliers solely on statistical grounds and prioritized subgroup analyses over meta-regression; with few studies per node and limited dose variability (eg, predominantly short-term VR studies), meta-regression was underpowered. Prespecified subgroup analyses by total training dose (high vs low), cognitive status, and intervention mode explored heterogeneity, with subgroup effects compared by interaction tests. Sensitivity analyses used the trim-and-fill method.

### Reporting Bias Assessment

To assess bias from missing results, we generated comparison-adjusted funnel plots and applied the Egger test [58]. Two reviewers (QH and SS) independently assessed reporting bias for each synthesis, resolving disagreements by discussion.

### Certainty Assessment

Certainty of evidence was assessed using the CINeMA framework [59], based on GRADE (Grading of Recommendations, Assessment, Development, and Evaluation) adapted for NMA [60]. In total, 6 domains were evaluated for each network comparison: within-study bias, reporting bias, indirectness, imprecision, heterogeneity, and incoherence. Within-study bias used the percentage contribution matrix; reporting bias was judged from funnel-plot asymmetry and the Egger test; indirectness from the match between study populations, interventions, and outcomes and the review question; imprecision from whether 95% CIs crossed a prespecified threshold (SMD 0.20); heterogeneity from *I*^2^; and incoherence from local node-splitting and the global design-by-treatment interaction test. Each domain was rated “no concerns,” “some concerns,” or “major concerns,” and ratings were summarized into an overall confidence rating (high, moderate, low, or very low). Two reviewers (QH and SS) assessed certainty independently, resolving disagreements by discussion, using the CINeMA web application [61].

### Ethical Considerations

This study involves the systematic review and secondary analysis of previously published data available in the public domain. As such, it did not involve direct contact with human participants or the collection of new primary data. Consequently, this study is exempt from institutional review board approval and informed consent requirements. The study protocol was prospectively registered with the International Prospective Register of Systematic Reviews (PROSPERO) under the registration number CRD420251030142.

## Results

### Study Selection

#### Flow of Studies

**Figure 1.**
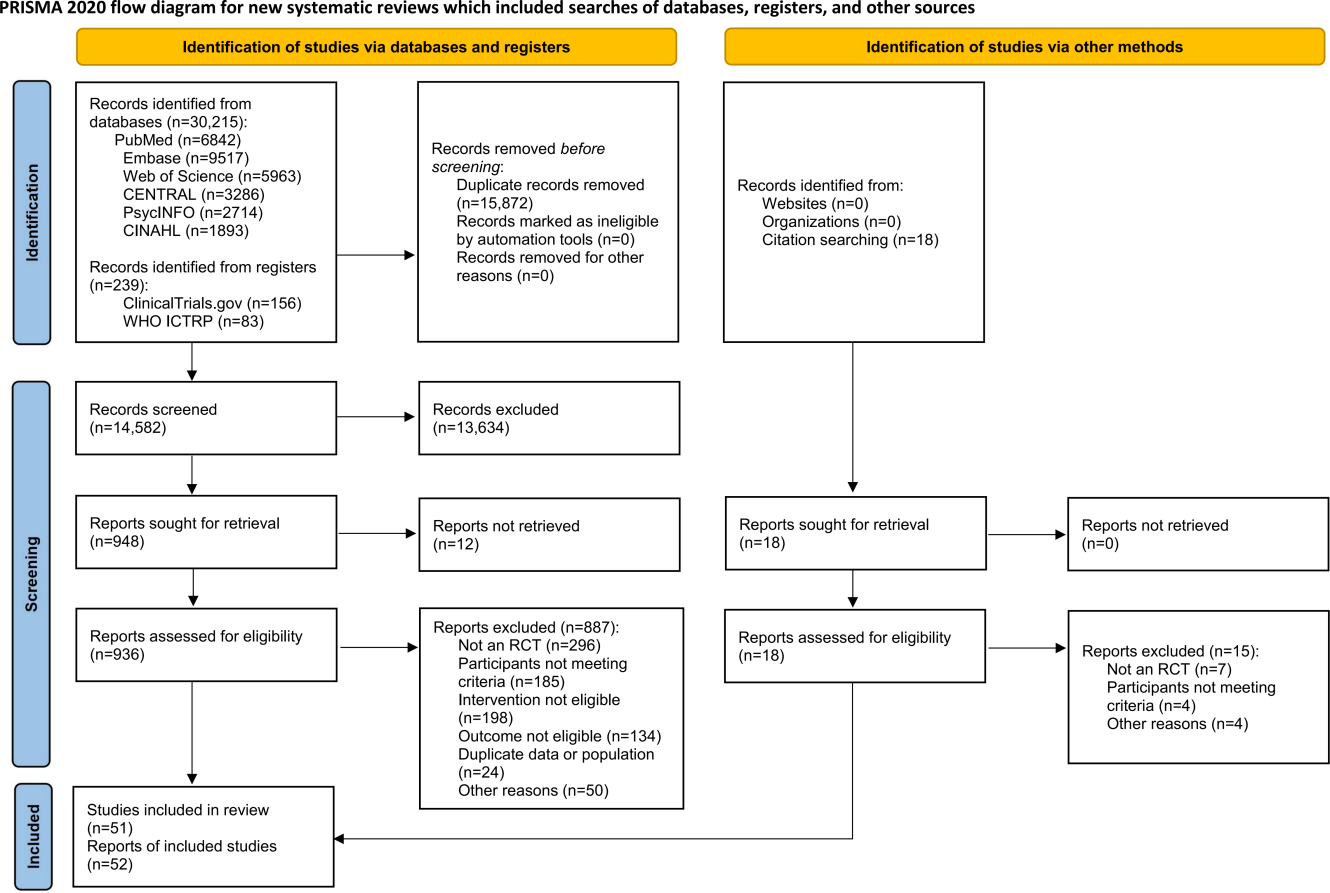
PRISMA flow diagram. PRISMA: Preferred Reporting Items for Systematic Reviews and Meta-Analyses; RCT: randomized controlled trial; WHO ICTRP: World Health Organization International Clinical Trials Registry Platform.

The database and register searches identified a total of 30,454 records (30,215 from databases and 239 from registers). After the removal of 15,872 duplicate records, 14,582 unique records remained for title and abstract screening. Of these, 13,634 were excluded, leaving 948 reports sought for full-text retrieval. In total, 12 reports could not be retrieved, resulting in 936 reports assessed for full-text eligibility. A total of 887 reports were excluded at this stage for the following reasons: not an RCT (n=296), participants not meeting criteria (n=185), intervention not eligible (n=198), outcome not eligible (n=134), duplicate data or population (n=24), and other reasons (n=50). Through other methods, 18 additional records were identified via citation searching of included studies and relevant previous reviews, of which 3 met the eligibility criteria after full-text assessment. In total, 52 reports were identified across all sources, corresponding to 51 unique studies, as 1 study published 2 reports sharing the same dataset [62,63]. Consequently, 51 studies were included in the final analysis (Figure 1).

#### Excluded Studies

Several studies that appeared potentially eligible were excluded after full-text assessment. For example, Begde et al [14] was excluded because it used a quasi-randomized design rather than a true RCT. Amjad et al [64] was excluded because the participants’ mean age was below 60 years. Further studies were excluded because the VR intervention focused on cognitive training without a physical exercise component; because participants were adults aged 50 years and older, which did not meet our age criterion of ≥60 years; and because the trial was a pilot study without randomized group allocation. A complete list of 39 studies excluded at full-text assessment, along with specific reasons for exclusion, is provided in Multimedia Appendix 3.

### Study Characteristics

The included studies comprised a total of 3673 participants (mean sample size per study 72, SD 68; range 10-349), with study-level mean ages ranging from 62.46 to 90.3 years (reported SDs ranged from 2.0 to 15.3 years). Regarding cognitive status, approximately half of the studies focused on cognitively healthy older adults (k=25), while the remaining studies targeted clinical populations, including MCI (k=14), dementia or major neurocognitive disorder (k=6), and subjective cognitive decline or at-risk groups (k=6). The average duration of interventions in the experimental groups was 12 (SD 5.4; range 2-26) weeks. Interventions were typically conducted with a frequency of 2‐3 sessions per week (SD 1.0; range 1-7 sessions), and the average session length was approximately 48 (SD 18; range 15-120) minutes.

Regarding different intervention types, 4 studies with a total of 252 participants examined the effects of IVR_E [17,65-67], 33 studies involving 2409 participants assessed NI_ExG [18,20-25,66,68-92], 7 studies with 591 participants investigated RE [16,32-34,93-95], and 8 studies including 488 participants explored VR_EC [15,19,62,96-100]. In terms of cognitive outcomes, 30 studies with a total of 2416 participants analyzed the effects on global cognition [15,16,20,21,25,32-34,62,67,70,71,73-82,86,90,92,94,97-100], 33 studies with 2467 participants examined executive function [15,17-20,22,23,25,33,34,62,65,66,68,69,72,73,76,79-85,87,89,91,93,95,96,98,100], and 20 studies with 1862 participants investigated memory function [15,22-25,33,62,65,66,68,69,76,79,80,82,85,86,88,90,96]. The detailed study characteristics are reported in Multimedia Appendix 4 [15-25,32-34,62,63,65-100].

### Risk of Bias in Studies

**Figure 2.**
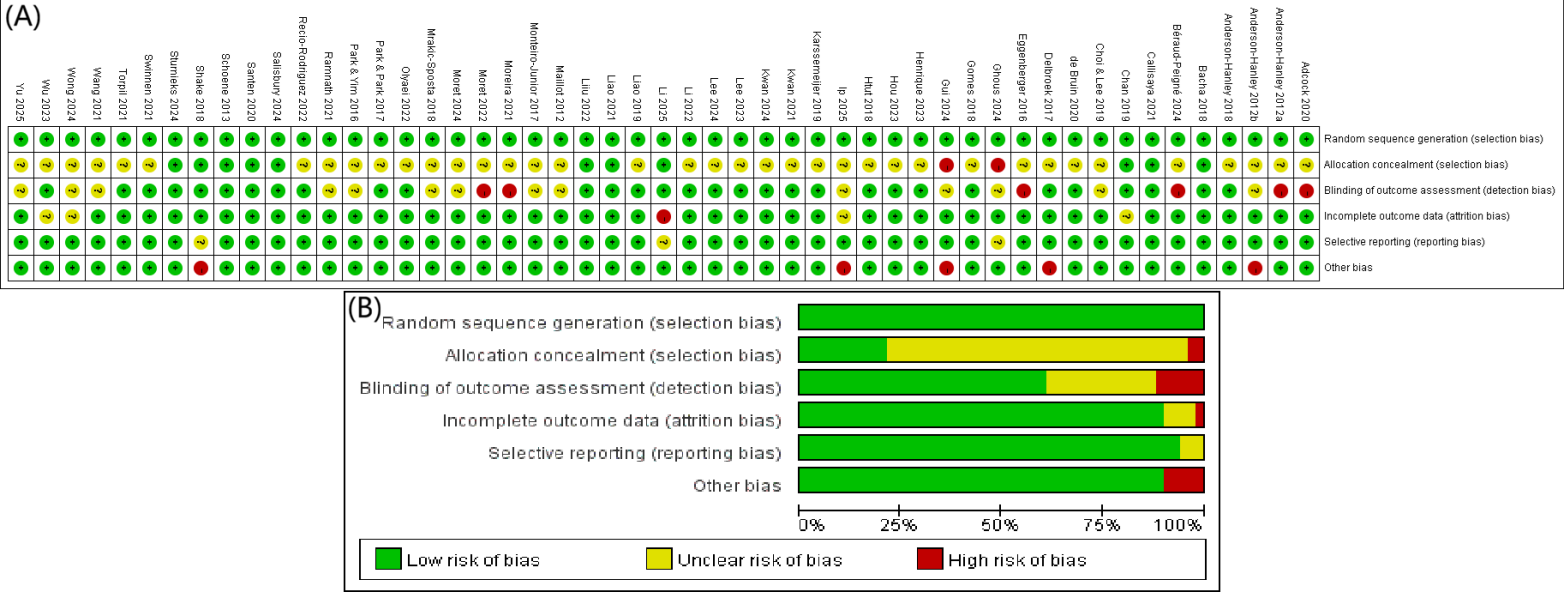
Bias risk assessment diagram. (A) Summary of the risk of bias for the included studies. Colors indicate the level of risk: green=low risk, yellow=unclear risk, and red=high risk. (B) Risk of bias graph for the included studies, showing the proportion of studies at each risk level across different bias domains [15-25,32-34,62,63,65-100].

We assessed the risk of bias in the 51 included studies, and the results are summarized in Figure 2. Among the individual domains, low risk of bias was most frequently observed in random sequence generation (51/51, 100%), incomplete outcome data (46/51, 90.2%), selective reporting (48/51, 94.1%), and other sources of bias (46/51, 90.2%). “Some concerns” were most common for allocation concealment (38/51, 74.5%) and blinding of outcome assessment (14/51, 27.5%). High risk of bias was relatively rare but present in allocation concealment (2/51, 3.9%), blinding of outcome assessment (6/51, 11.8%), incomplete outcome data (1/51, 2.0%), and other sources of bias (5/51, 9.8%).

Based on the “worst domain” rule, 8 studies were rated as low risk overall, 30 studies as having some concerns, and 13 studies as high risk of bias. Overall, these findings indicate that while random sequence generation, outcome reporting, and handling of missing data were generally robust, allocation concealment and blinding of outcome assessment remain the most frequent sources of potential bias. Attention to these domains in future trials is warranted to further improve methodological quality.

### Results of Individual Studies

**Figure 3.**
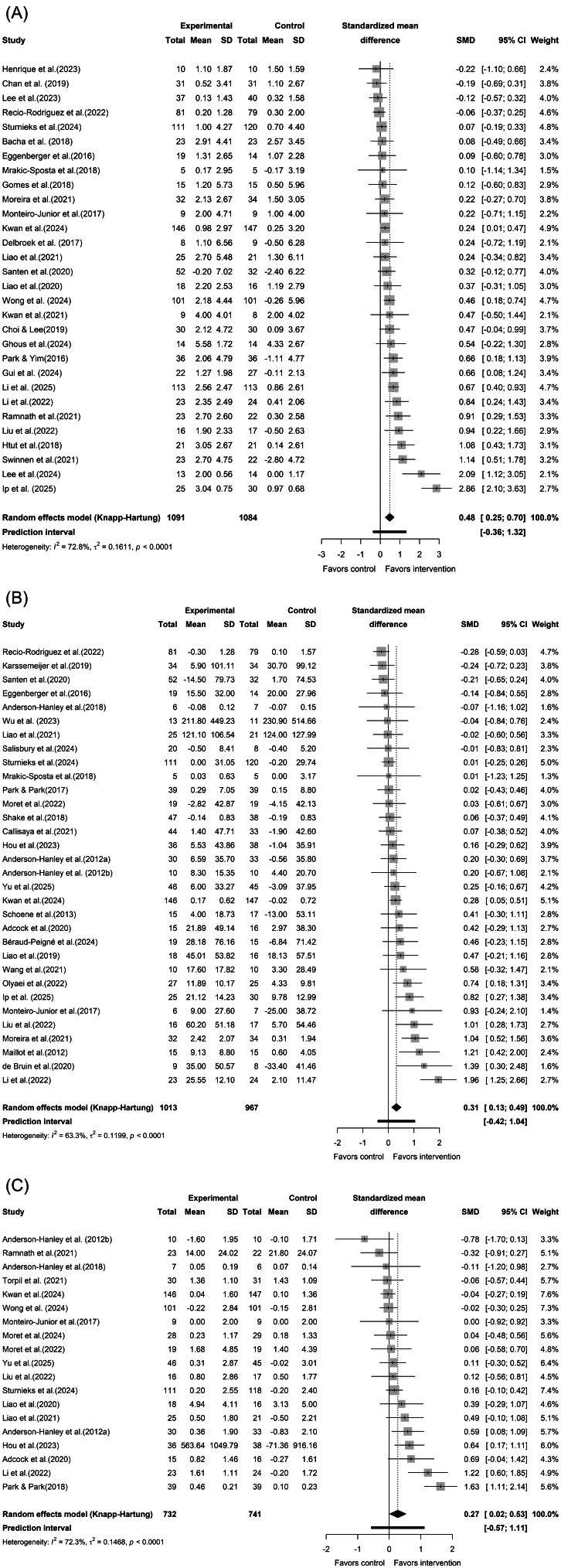
Forest plots. The forest plots show the pooled SMDs of digital physical exercise interventions for cognitive functions in older adults. (A) Global cognition, (B) executive function, and (C) memory function. Squares represent the effect estimate of individual studies (with size proportional to study weight), horizontal lines represent 95% CIs, and diamonds indicate the pooled summary estimates [15-25,32-34,62,63,65-100].

**Figure 4.**
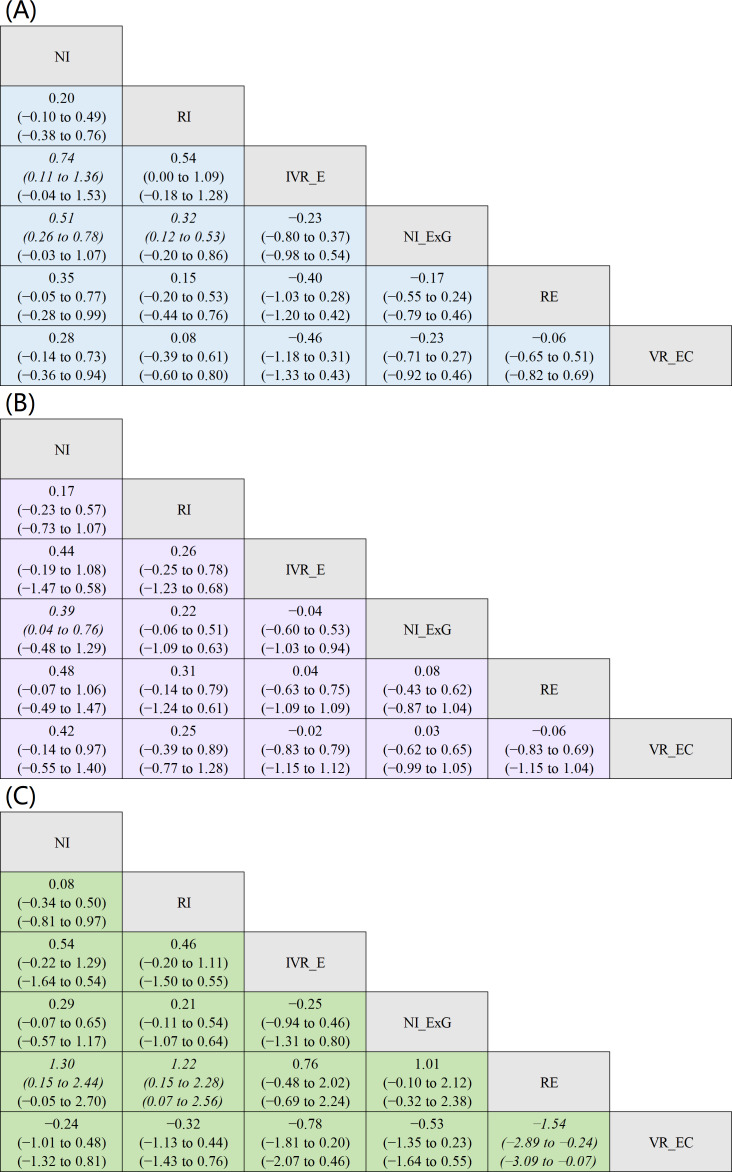
League tables. (A) Global cognition (blue), (B) executive function (purple), and (C) memory function (green). Each cell presents the SMD (top), 95% CI (middle), and PI (bottom). Statistically significant effects (based on 95% CI) are shown in italics format. IVR_E: immersive virtual reality exercise; NI: nonintervention; NI_ExG: nonimmersive exergame; PI: prediction interval; RE: remote exercise; RI: routine intervention; SMD: standardized mean difference; VR_EC: virtual reality exercise combined with cognitive training.

Study-level summary statistics, including sample sizes, means, and SDs for both experimental and control groups, along with individual effect estimates (SMD and 95% CI), are presented in Figure 3. These forest plots present direct pairwise meta-analysis results only (Table 1); the corresponding NMA estimates for the same comparisons, which incorporate indirect evidence, are reported in the text and in the league table (Figure 4) and may differ numerically. The overall pooled effect for global cognition across 30 studies was SMD 0.48 (95% CI 0.25-0.70; PI −0.36 to 1.32; *I*^2^=72.8%), representing a small-to-moderate effect. For executive function, analysis of 32 studies yielded SMD 0.31 (95% CI 0.13-0.49; PI −0.42 to 1.04; *I*^2^=63.3%), representing a small effect. Memory function analysis across 19 studies showed SMD 0.27 (95% CI 0.02-0.53; PI −0.57 to 1.11; *I*^2^=72.3%), also representing a small effect. Substantial heterogeneity was observed across all 3 outcomes. Notably, Gui et al [76] was retained in the global cognition synthesis, given the availability of complete pre- and postintervention data, but was excluded from the executive function and memory function analyses as only preintervention data were reported for those outcomes.

**Table 1.** Pairwise meta-analyses of cognitive outcomes.

Cognitive outcomes and subgroup project	Studies, k	Heterogeneity test results		Meta-analysis results	
		*P* value	*I*^2^ (%)	SMD (95% CI)	*P* value
Global cognition (excluding outliers)					
IVR_E^a^ vs RI^b^	2	.50	0.00	0.56 (−1.96 to 3.09)	.22
NI_ExG^c^ vs NI^d^	6	.34	11.20	0.60 (0.36 to 0.83)	<.001
NI_ExG vs RI	12	.06	41.50	0.25 (0.00 to 0.50)	.05
RE^e^ vs NI	1	—^f^	—	−0.13 (−0.57 to 0.32)	.59
RE vs RI	3	.02	75.40	0.37 (−0.82 to 1.56)	.31
VR_EC^g^ vs NI	3	.98	0.00	0.23 (−0.25 to 0.71)	.18
VR_EC vs RI	1	—	—	0.47 (−0.50 to 1.44)	.34
Executive function					
IVR_E vs NI_ExG	1	—	—	−0.07 (−1.16 to 1.02)	.90
IVR_E vs RI	4	.76	0.00	0.28 (−0.24 to 0.80)	.18
NI_ExG vs NI	7	.02	59.30	0.33 (−0.12 to 0.79)	.12
NI_ExG vs RI	11	<.001	64.90	0.30 (−0.06 to 0.65)	.09
RE vs NI	1	—	—	0.41 (−0.30 to 1.11)	.26
RE vs RI	4	<.001	90.70	0.39 (−1.16 to 1.95)	.48
VR_EC vs NI	3	.18	41.70	0.42 (−0.46 to 1.30)	.17
VR_EC vs RI	1	—	—	0.20 (−0.67 to 1.08)	.65
Memory function					
IVR_E vs RI	2	.65	0.00	0.52 (−2.12 to 3.15)	.24
IVR_E vs NI_ExG	1	—	—	−0.11 (−1.20 to 0.98)	.84
NI_ExG vs NI	7	.23	26.30	0.18 (−0.08 to 0.39)	.15
NI_ExG vs RI	6	<.001	85.00	0.33 (−0.40 to 1.06)	.30
RE vs RI	1	—	—	1.22 (0.60 to 1.85)	<.001
VR_EC vs NI	1	—	—	−0.04 (−0.27 to 0.19)	.73
VR_EC vs RI	1	—	—	−0.78 (−1.70 to 0.13)	.09

a IVR_E: immersive virtual reality exercise.

b RI: routine intervention.

c NI_ExG: nonimmersive exergame.

d NI: nonintervention.

e RE: remote exercise.

f Not available.

g VR_EC: virtual reality exercise combined with cognitive training.

### Results of Syntheses

#### Pairwise Meta-Analysis

Pairwise meta-analyses were conducted for each direct comparison within each cognitive outcome domain, with results presented in Table 1. After applying the HKSJ adjustment, most direct comparisons did not reach statistical significance, consistent with the wider, more conservative CIs this method yields. For global cognition, the only clearly significant comparison was NI_ExG versus NI (SMD 0.60, 95% CI 0.36-0.83); NI_ExG versus RI was marginal, with its lower confidence limit at the null (SMD 0.25, 95% CI 0.00-0.50; *P*=.05), whereas comparisons involving IVR_E, RE, and VR_EC did not reach significance. For executive function, no direct comparison remained statistically significant after the HKSJ adjustment, although point estimates were generally positive. For memory function, the only significant comparison was RE versus RI (SMD 1.22, 95% CI 0.60-1.85); however, this estimate derives from a single study and therefore requires cautious interpretation, given the absence of replication. Heterogeneity was substantial in several comparisons—most notably RE versus RI (*I*^2^=75.4% for global cognition and 90.7% for executive function) and NI_ExG versus RI (*I*^2^=64.9% for executive function and 85% for memory)—and several comparisons were informed by only one study, both of which limit the reliability of the pooled estimates. NI_ExG nonetheless rested on the largest evidence base across comparisons. Overall, once between-study uncertainty was fully propagated through the HKSJ correction, robust direct evidence was confined to NI_ExG versus NI for global cognition, indicating that most modality-specific pairwise effects should be interpreted with caution pending further trials.

#### NMA

**Figure 5.**
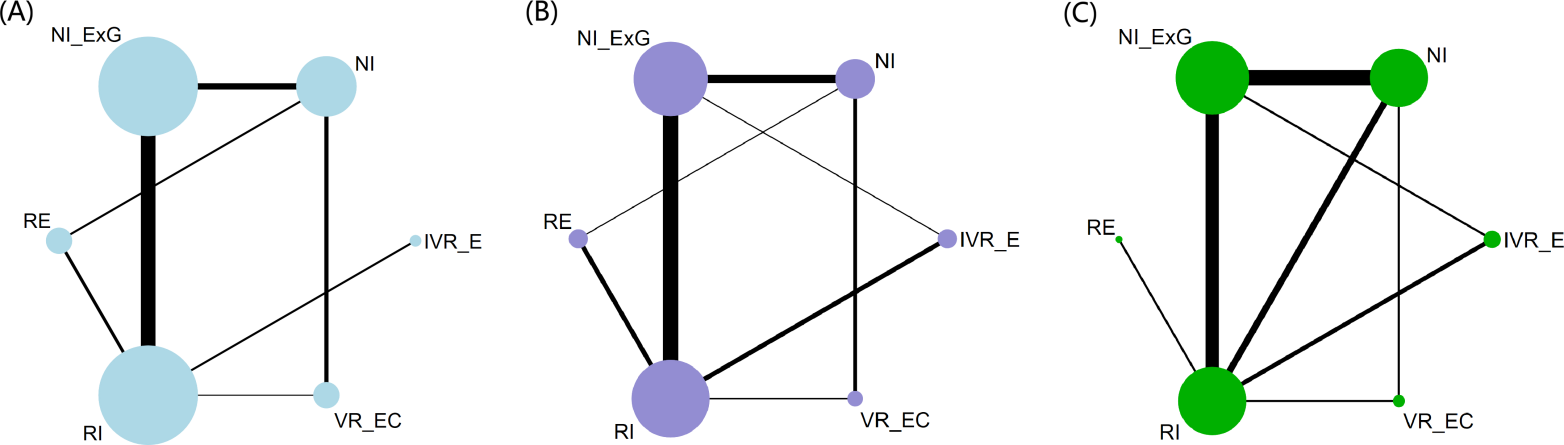
Network plots. The network plots for (A) global cognition, (B) executive function, and (C) memory function are shown. The width of each line indicates the number of studies comparing each pair of treatments, while the size of each circle reflects the sample size in each study arm. IVR_E: immersive virtual reality exercise; NI: nonintervention; NI_ExG: nonimmersive exergame; RE: remote exercise; RI: routine intervention; VR_EC: virtual reality exercise combined with cognitive training.

The NMA of cognitive outcomes, including global cognition, executive function, and memory function, compared different types of digital physical exercise interventions with one another and with the comparison groups RI and NI (Figure 5).

The relative effect estimates for all pairwise comparisons from the NMA are presented in the league table (Figure 4), with each cell displaying the SMD, 95% CI, and PI. Because network estimates for a given comparison combine direct and indirect evidence and enforce consistency across the entire network, they may differ numerically from the corresponding direct pairwise estimates reported in Table 1 and Figure 3 for the same comparison.

For global cognition, significant effects were observed for NI_ExG versus NI (SMD 0.51, 95% CI 0.26-0.78) and NI_ExG versus RI (SMD 0.32, 95% CI 0.12-0.53), indicating that NI_ExG consistently outperformed both control conditions. IVR_E also demonstrated a significant advantage over NI (SMD 0.74, 95% CI 0.11-1.36). However, the PIs for all significant comparisons crossed 0, suggesting that these benefits may not be consistently reproduced across all future clinical settings. No significant differences were observed between any 2 active digital intervention modalities.

For executive function, only NI_ExG versus NI reached statistical significance (SMD 0.39, 95% CI 0.04-0.76). All remaining comparisons, including those involving IVR_E, RE, and VR_EC, yielded CIs crossing 0. PIs similarly crossed 0 for all comparisons, indicating limited certainty in the generalizability of findings.

For memory function, RE demonstrated significant advantages over both NI (SMD 1.30, 95% CI 0.15-2.44) and RI (SMD 1.22, 95% CI 0.15-2.28). Notably, RE versus RI was the only comparison across all 3 outcomes for which the PI also excluded 0 (PI 0.07-2.56), suggesting that this effect may be more consistently replicated in future studies. VR_EC was significantly inferior to RE (SMD −1.54, 95% CI −2.89 to −0.24), and this was likewise the only head-to-head comparison between active digital modalities to reach significance across all outcomes; its PI also excluded 0 (PI −3.09 to −0.07), reinforcing the robustness of this finding. No other comparisons between active digital modalities reached statistical significance.

**Figure 6.**
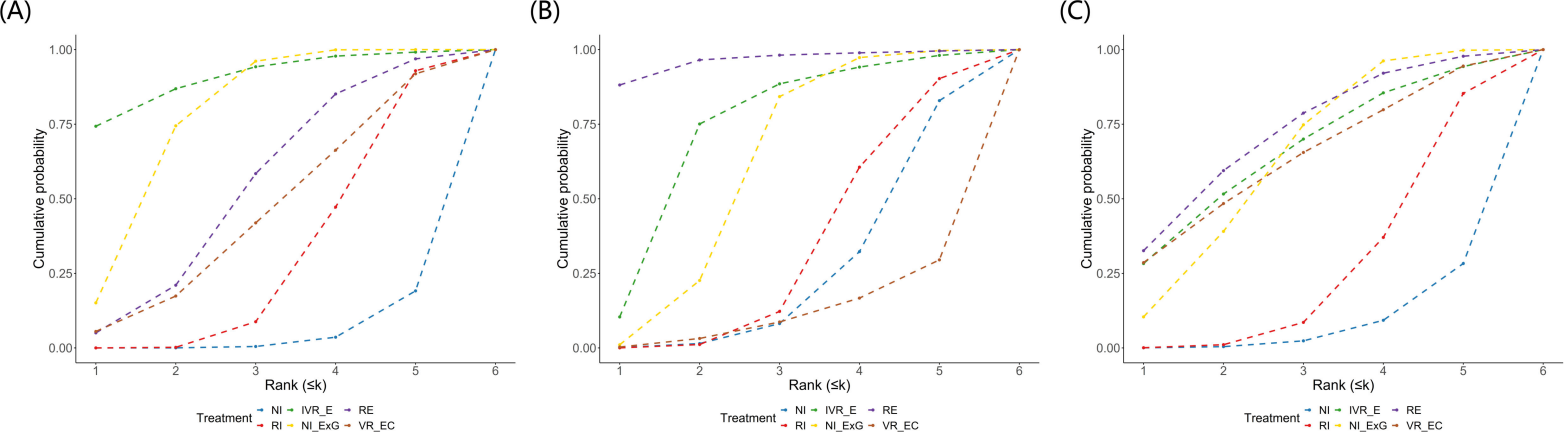
SUCRA plots. (A) Global cognition, (B) executive function, and (C) memory function. Each colored line represents a different intervention. A larger area under the curve indicates a higher SUCRA value, reflecting a more favorable ranking. IVR_E: immersive virtual reality exercise; NI: nonintervention; NI_ExG: nonimmersive exergame; RE: remote exercise; RI: routine intervention; SUCRA: surface under the cumulative ranking curve; VR_EC: virtual reality exercise combined with cognitive training.

The hierarchy of intervention efficacy was estimated using SUCRA values (Figure 6), where higher percentages indicate a greater probability of being the most effective treatment, and full ranking probabilities are detailed in Multimedia Appendix 5. Regarding global cognition, immersive VR emerged as the clear frontrunner with the highest SUCRA value of 96.6%, followed by NI_ExGs at 76.4%. RE ranked third with 57.6%, while VR_EC showed a moderate ranking of 40.3%. Furthermore, RI and NI had the lowest probabilities of being the best option.

In terms of executive function, the rankings were more distributed among the top interventions. Specifically, RE ranked highest at 73.8%, closely followed by NI_ExGs at 69.3% and immersive VR at 67.6%. These 3 digital modalities formed the top tier of interventions, and all substantially outperformed RI and NI.

Finally, concerning memory function, immersive VR achieved a dominant ranking of 82.8%, which is consistent with the significant findings in the pairwise analysis. RE also performed strongly with a value of 76.6%. In contrast, NI_ExGs and VR_EC ranked lower, while RI performed comparably to some digital interventions in this domain.

Consistency tests for global cognition, executive function, and memory function support the consistency hypothesis of the network model. Inconsistency analysis using the node-splitting method identified potential inconsistency between direct and indirect comparisons for the NI_ExG versus NI comparison in global cognition (*P*=.03), whereas no evidence of inconsistency was observed for the remaining comparisons. The detailed results are presented in Multimedia Appendix 5.

### Heterogeneity Investigations

The overall pairwise meta-analysis revealed significant heterogeneity across all 3 cognitive domains: global cognition (k=30; *I*^2^=72.8%; *P*<.001), executive function (k=32; *I*^2^=63.3%; *P*<.001), and memory function (k=19; *I*^2^=72.1%; *P*<.001). To investigate the potential sources of the observed heterogeneity, we conducted prespecified subgroup analyses based on (1) cognitive status (healthy vs impaired), (2) interventions, and (3) dose (high [>1000 minutes] vs low [≤1000 minutes]). Heterogeneity statistics are summarized in Table 2. Detailed analyses of heterogeneity and subgroup analyses are presented in Multimedia Appendix 6 [15-25,32-34,62,63,65-100].

**Table 2.** Summary of heterogeneity and subgroup analyses for cognitive outcomes.

Subgroup category	Global cognition			Executive function			Memory function		
	Studies, k	*I*^2^ (%)	*P* value	Studies, k	*I*^2^ (%)	*P* value	Studies, k	*I*^2^ (%)	*P* value
Overall	30	72.80	<.001	32	63.30	<.001	19	72.10	<.001
Overall (excluding outliers)	28	49.20	<.001	N/A^a^	N/A	N/A	N/A	N/A	N/A
Cognitive status									
Cognitively healthy	14	48.50	.02	18	53.60	<.001	10	42.80	.07
Cognitive impairment	14	16.10	.28	14	72.40	<.001	9	83.60	<.001
Interventions									
IVR_E^b^	2	0.00	.50	5	0.00	.82	2	0.00	.65
NI_ExG^c^	18	53.10	<.001	18	60.90	<.001	14	69.90	<.001
RE^d^	4	69.20	.02	5	87.90	<.001	1	—^e^	—
VR_EC^f^	4	0.00	.97	4	15.10	.32	2	57.80	.12
Total dose									
High dose (>1000 minutes)	11	46.60	.04	22	67.90	<.001	12	49.30	.03
Low dose (≤1000 minutes)	16	43.60	.03	10	21.20	.25	7	84.70	<.001

a N/A: not applicable; no outliers were identified for these outcomes.

b IVR_E: immersive virtual reality exercise.

c NI_ExG: nonimmersive exergame.

d RE: remote exercise.

e Not available; heterogeneity statistics could not be calculated because only 1 study was included.

f VR_EC: virtual reality exercise combined with cognitive training.

For global cognition, preliminary subgroup analyses across all 3 categories failed to identify a clear source of heterogeneity. Visual inspection of individual study effect estimates identified 2 outlier studies with unusually large effect sizes—Lee et al [32] and Ip et al [98]—which were subsequently excluded as sensitivity analyses. Following exclusion, overall heterogeneity was substantially reduced (from *I*^2^=72.8% to 49.2%), and cognitive status emerged as a meaningful source of heterogeneity. In the cognitively healthy subgroup, heterogeneity was moderate (*I*^2^=48.5%; *P*=.02), while the cognitively impaired subgroup demonstrated markedly lower heterogeneity (*I*^2^=16.1%; *P*=.28), suggesting more consistent intervention effects in populations with existing cognitive deficits. As no outliers were identified for executive function or memory function, all studies were retained for those outcomes.

Regarding intervention type, IVR_E showed no heterogeneity across all 3 outcomes (*I*^2^=0% for global cognition, executive function, and memory function), indicating highly reproducible effects regardless of cognitive domain. VR_EC similarly demonstrated low-to-negligible heterogeneity for global cognition (*I*^2^=0%) and executive function (*I*^2^=15.1%), though it showed moderate heterogeneity for memory function (*I*^2^=57.8%). In contrast, NI_ExG exhibited persistent moderate-to-high heterogeneity across all 3 outcomes (*I*^2^=53.1%, 60.9%, and 69.9%, respectively), likely reflecting the considerable diversity in devices, game content, and protocols within this category. RE showed high heterogeneity for both global cognition (*I*^2^=69.2%) and executive function (*I*^2^=87.9%).

For total training dose, patterns differed across outcomes. For global cognition, both high-dose (*I*^2^=46.6%) and low-dose (*I*^2^=43.6%) subgroups showed comparable, moderate heterogeneity. For executive function, low-dose interventions yielded substantially lower heterogeneity (*I*^2^=21.2%; *P*=.25) compared to high-dose interventions (*I*^2^=67.9%), suggesting greater consistency among shorter programs. For memory function, the pattern was reversed: high-dose interventions showed moderate heterogeneity (*I*^2^=49.3%; *P*=.03), while low-dose interventions remained highly heterogeneous (*I*^2^=84.7%), suggesting that sufficient training volume may be associated with more stable memory outcomes.

### Sensitivity Analyses

Sensitivity analyses were conducted using the leave-one-out method, in which each study was systematically omitted, and the remaining studies were reanalyzed to assess the robustness of the pooled estimates. Results are presented in Multimedia Appendix 6.

For all 3 cognitive outcomes, the pooled effect estimates remained stable across all leave-one-out iterations, with point estimates consistently falling within a narrow range: approximately 0.20 to 0.52 for global cognition, 0.07 to 0.42 for executive function, and 0.02 to 0.53 for memory function. No single study, when omitted, shifted the pooled estimate outside the original CI or altered the direction of effect. These findings confirm that the main results are not unduly influenced by any individual study and are robust to the exclusion of any single observation.

### Reporting Biases

Reporting biases were assessed using comparison-adjusted funnel plots and the Egger test for each cognitive outcome (Multimedia Appendix 7). For global cognition, the funnel plot appeared approximately symmetrical, and the Egger test indicated no statistically significant asymmetry (*t*_26_=−1.59; *P*=.12), suggesting no evidence of reporting bias. Similarly, for memory function, the funnel plot was largely symmetrical, and the Egger test was nonsignificant (*t*_17_=−0.42; *P*=.68), providing no indication of publication bias.

For executive function, the Egger test revealed statistically significant funnel plot asymmetry (*t*_30_=−3.00; *P*=.005). A subsequent trim-and-fill analysis was conducted to explore the potential impact of missing studies; however, the method estimated 0 missing studies (*P*=.0001), indicating that the observed asymmetry was unlikely to be attributable to selective nonpublication of small negative studies. The asymmetry may instead reflect genuine heterogeneity in effect sizes or other small-study effects. Taken together, these findings suggest that reporting bias does not substantially threaten the validity of the synthesized results across the 3 cognitive outcomes.

### Certainty of Evidence

The GRADE summary of findings table and the detailed domain-level CINeMA assessment for all comparisons across the 3 cognitive outcomes are presented in Multimedia Appendix 8.

For global cognition, the certainty was rated as low for one comparison—NI_ExG versus RI (14 direct studies)—downgraded primarily for within-study bias and heterogeneity. The remaining 14 comparisons were rated as very low, with the most common reasons for downgrading being within-study bias (all 15 comparisons, attributable to predominantly unclear allocation concealment), heterogeneity, and incoherence. Incoherence was a major concern for 8 comparisons with no direct evidence, where the network relied entirely on indirect estimates. Reporting bias and indirectness raised no concerns across any global cognition comparison.

For executive function, all 15 comparisons were rated as very low certainty. Notably, reporting bias was rated as “some concerns” for all comparisons—a pattern distinct from the other 2 outcomes—reflecting the significant funnel plot asymmetry detected by the Egger test. Within-study bias and imprecision were the other predominant downgrading factors, with incoherence raising major concerns for all 6 comparisons supported only by indirect evidence.

For memory function, the certainty was more variable. One indirect comparison—RE versus VR_EC—was rated as moderate, downgraded only for within-study bias. Two comparisons reached low certainty: RE versus RI (1 direct study; downgraded for within-study bias and heterogeneity) and the indirect comparison NI versus RE (downgraded for within-study bias and heterogeneity). The remaining 12 comparisons were rated as very low, with within-study bias and imprecision the most common downgrading factors. Indirectness and reporting bias raised no concerns for any memory function comparison.

Across all 3 outcomes, indirectness was consistently rated as no concern, reflecting the good alignment between the included studies and the review question. The pervasive within-study bias concerns—driven by unclear allocation concealment in the majority of included RCTs—and the high proportion of very low certainty ratings across all outcomes indicate that the current evidence base, while suggestive of benefit, should be interpreted with caution.

## Discussion

### Principal Findings

In line with our prespecified aims, this Bayesian NMA ranked IVR_E, NI_ExG, RE, and VR_EC against RI and NI controls on global cognition, executive function, and memory function in older adults. The principal finding is that comparative effectiveness is highly domain-specific rather than uniformly favoring the most technologically immersive option. NI_ExG produced the most consistent global-cognition improvements and was the only modality with a statistically significant advantage for executive function. RE was the only modality with statistically significant memory benefits over both controls and, uniquely among all comparisons, its PI also excluded the null, indicating the finding most likely to generalize to future clinical settings [39,57].

Although IVR_E consistently achieved the highest SUCRA ranking for global cognition and memory, its PIs crossed the null in every domain, signaling uncertainty about whether these advantages would replicate in subsequent trials [39]. VR_EC was the only active modality significantly inferior to another digital modality, namely RE, on memory. Subgroup analyses indicated more stable memory benefits when cumulative training exceeded approximately 1000 minutes, with no analogous threshold for the other 2 outcomes. Collectively, these results refine—rather than confirm—the prevailing notion in earlier exergame and immersive-VR reviews that highly immersive technologies are uniformly the most effective digital exercise format for cognitive health in older adults [31,101]; instead, they support domain-tailored selection among digital modalities.

The robust and consistent benefit of NI_ExG for global cognition aligns with previous single-modality exergame reviews, which reported moderate improvements in older adults’ overall cognitive performance [28-30]. Our network-based approach extends this by ranking NI_ExG against 3 other digital modalities within a single framework and showing it was the only modality with a statistically significant advantage over RI in this domain. Mechanistically, the NI_ExG platforms in our network (eg, Wii and Kinect) integrate moderate-intensity gross-motor activity with cognitively engaging tasks such as target tracking, motor planning, and real-time decision-making, producing a dual-task demand theorized to drive the synergistic benefits of combined physical-cognitive interventions in aging [73,77,102]—consistent with recent NMAs identifying mind-body and dual-task interventions as more effective than passive controls [35,36]. This suggests that the cognitive demand embedded within gameplay, rather than the digital delivery itself, accounts for the consistent global-cognition benefit.

Although IVR_E achieved the highest SUCRA value, the wide PIs in every IVR_E comparison reflect both the limited number of contributing studies and substantial between-study variation in immersive hardware, content, and exposure duration. Because SUCRA values capture relative ranking but not the magnitude of differences or the certainty of evidence, high rankings from sparse networks should not be interpreted as direct evidence of superiority [103,104]; read alongside the corresponding PIs [39,57], the IVR_E global-cognition results are best regarded as hypothesis-generating rather than conclusive. Similarly, the absence of statistically significant differences between any 2 active digital modalities likely reflects network sparsity together with the moderate effect sizes typical of cognitive interventions, rather than true equivalence among modalities [103]. After excluding 2 outlier studies in sensitivity analyses, cognitive status emerged as the principal source of heterogeneity in this synthesis, with the cognitively impaired subgroup showing markedly lower variability than the healthy subgroup—a pattern that may reflect a wider margin for measurable improvement and is consistent with prior evidence that physical-activity benefits are well documented in MCI and dementia [46].

The finding that NI_ExG was the only digital modality with a statistically significant advantage for executive function, and only relative to NI, parallels earlier meta-analyses focused exclusively on exergaming and executive function in older adults [26,105]. Mechanistically, NI_ExG platforms place sustained demands on the core executive subprocesses—response inhibition, working memory, and cognitive flexibility—by requiring real-time monitoring of moving targets, suppression of prepotent responses, and rapid task switching during gameplay [48,49,102]. Empirical evidence from our included trials supports this, with studies linking exergame training to increased prefrontal activation during walking [73] and to neuroplastic and inflammatory changes consistent with executive improvement [77]. The nonsignificance of NI_ExG against RI may reflect the heterogeneous composition of our active-control category, which included nondigital traditional exercise and isolated cognitive training—interventions that themselves can produce modest executive gains and thereby narrow the contrast [35,36].

By contrast, IVR_E showed remarkable cross-study consistency for executive function but failed to reach statistical significance against any control, suggesting that high immersion—absent an explicitly executive-targeted task design—may not sufficiently stimulate the prefrontal control circuitry; recent evidence indicates that the executive benefits of VR are driven mainly by trials integrating explicit cognitive challenges with movement, rather than by sensory immersion per se [31,105]. The close clustering of SUCRA values among RE, NI_ExG, and IVR_E, combined with PIs that crossed the null in every comparison, further indicates that ranking metrics alone cannot reliably discriminate among these modalities [39,57,103]. The very high within-modality heterogeneity observed for RE also tempers its top SUCRA position, given that RE protocols ranged from cognitively passive aerobic content to dual-task formats. Subgroup analyses showed that lower-dose interventions yielded substantially less heterogeneity than higher-dose ones, possibly reflecting more uniform protocol delivery in shorter programs and warranting confirmation in dose-stratified trials. Finally, the Egger-test funnel asymmetry for executive function, unaccompanied by any imputed studies in the trim-and-fill analysis, more plausibly reflects between-study heterogeneity than selective nonpublication of small negative trials [57].

The most distinctive finding concerns the memory domain: RE, rather than the more technologically immersive modalities, produced the only statistically robust improvement in memory function. It was the sole modality to outperform both control conditions and—uniquely among all comparisons across the 3 outcomes—its PI against RI also excluded the null, indicating that the effect is more likely to generalize to future clinical settings [39,57]. Mechanistically, this is biologically plausible, given that structured aerobic and combined exercise upregulates brain-derived neurotrophic factor and insulin-like growth factor 1, increases hippocampal volume, and enhances memory performance in older adults [106,107]. A recent home-based exercise randomized trial in community-dwelling older adults further demonstrated dose-related improvements in episodic and working memory following remotely supervised training, directly supporting our observation that RE confers memory benefit [108]. Nevertheless, this finding warrants caution: it derives from a single direct RE-versus-RI comparison, the corresponding GRADE certainty was low [60], and replication in adequately powered multisite trials is essential before any firm clinical recommendation.

The second distinctive memory finding is that VR_EC was the only active digital modality significantly inferior to another active modality, namely RE, with both the CIs and PIs excluding the null. Two complementary mechanisms may contribute. First, the dual-task structure of VR_EC imposes competing cognitive demands during the encoding window, and recent randomized evidence indicates that superimposing cognitive training on home-based exercise can attenuate, rather than augment, exercise-induced gains in episodic memory [108]. Second, immersive head-mounted VR can elicit visual-vestibular conflict, headset discomfort, and variable acceptance in older adults, which may compromise the engagement and consolidation processes required for memory benefit [109,110]. By contrast, IVR_E achieved the highest SUCRA value for memory but did not reach significance against any control; as in the other domains, this most plausibly reflects the small number of contributing studies, the wide PIs, and SUCRA’s insensitivity to certainty of evidence [103], so the apparent advantage is best interpreted as hypothesis-generating. Subgroup analyses provided additional context: unlike the other outcomes, lower cumulative training doses yielded substantially greater heterogeneity for memory than higher doses, suggesting that an adequate cumulative training volume may be necessary for stable memory gains, in line with prior dose-response evidence from aerobic exercise interventions [106].

### Limitations of the Evidence

Several limitations of the evidence base warrant consideration. First, CINeMA certainty was very low for most comparisons across all 3 outcomes, with only one moderate rating [59,60]; within-study bias—mainly unclear allocation concealment—was the most pervasive downgrading factor, and about a quarter of studies were at high overall risk of bias, so findings are not confirmatory without further high-quality replication. Second, substantial heterogeneity occurred across all domains, particularly within NI_ExG (diverse hardware, game content, and supervision) and RE for executive function (protocols ranging from passive aerobic to dual-task), constraining the precision of pooled estimates. Third, the evidence is sparse for several modalities: few trials contributed to the IVR_E network, and several comparisons rested on only 1 or 2 direct studies, widening PIs and limiting SUCRA’s discriminatory power [103,104]. Most consequentially, RE’s robust memory advantage rests on a single direct comparison; although its PI uniquely excluded the null [39,57], multisite replication is essential before firm clinical recommendations. Finally, all trials provided only short- to medium-term data (mean approximately 12 weeks), with no eligible follow-up beyond the immediate postintervention period, so whether benefits translate into delayed cognitive decline or dementia onset remains unknown.

### Limitations of the Review Processes

Several process limitations should also be acknowledged. First, eligibility was restricted to English-language publications in 5 of the 6 databases; although CENTRAL had no language restriction, potentially eligible non-English trials may have been missed. This is a nontrivial concern here, as a substantial body of digital exercise and cognitive intervention research has been conducted in China and other non-English–speaking regions and is frequently published in local-language journals [28,30]. Their exclusion may have underrepresented modalities actively studied in these settings—particularly exergame and VR-based interventions—reducing the comprehensiveness of the evidence base and the precision of sparsely populated nodes such as IVR_E; where intervention delivery, cultural context, or participant characteristics differ across linguistic regions, the generalizability of our estimates to non-English–speaking populations may also be limited. Future reviews incorporating major Chinese-language databases such as the China National Knowledge Infrastructure and Wanfang Database would help quantify this potential language bias. Second, search strategies were not formally peer-reviewed (eg, via the PRESS checklist), study authors were not contacted, and gray literature was not systematically browsed, so some unpublished or in-progress trials may have escaped detection. Third, although the executive-function funnel asymmetry from the Egger test was unaccompanied by imputed studies in trim-and-fill [58], small-study effects or unmeasured reporting biases cannot be excluded, so GRADE reporting-bias downgrading was applied across all executive function comparisons. Fourth, grouping interventions into 4 broad modalities may have collapsed within-category heterogeneity, particularly for NI_ExG; finer categorization was precluded by sparse nodes, and limited dose variability left meta-regression underpowered, so dichotomized-dose subgroup analyses served as a pragmatic but coarser alternative. Finally, although node-splitting supported consistency across all 3 outcomes, several comparisons—particularly in memory—relied entirely on indirect evidence, contributing to the predominance of very low GRADE certainty ratings.

### Implications

To our knowledge, this is the first Bayesian NMA to simultaneously compare and rank 4 distinct categories of digital physical exercise—IVR_E, NI_ExG, RE, and VR_EC—against both active and passive controls across 3 cognitive domains in older adults spanning the full cognitive spectrum. It differs from prior work in 2 respects: existing NMAs have largely addressed traditional exercise without differentiating digital formats [8,25,36], while reviews of digital modalities have mostly used a single-modality lens or restricted populations to MCI or dementia [26,27,29-31]. By synthesizing direct and indirect evidence within one connected network, calculating PIs for all comparisons, and providing GRADE certainty ratings across outcomes, it offers the most analytically comprehensive multimodal comparison of digital physical exercise for cognitive health in older adults to date.

The practical implications operate at 3 levels. Clinically, the results support domain-tailored modality selection: NI_ExG for global cognition or executive function; RE for memory, with attention to adequate cumulative training dose; and IVR_E, given its cross-study consistency, for standardized institutional settings pending confirmatory trials. For public health, the low-cost scalability of RE and NI_ExG without specialized facilities makes them particularly relevant for older adults in rural and resource-limited settings [10,13]. For policy, the findings provide a modality-specific evidence base to inform guideline development, digital health investment, and research funding priorities for cognitive health in aging populations [6].

### Conclusions

This Bayesian NMA provides the first integrated comparison of 4 digital physical exercise categories—IVR_E, NI_ExG, RE, and VR_EC—against active and passive controls across 3 cognitive domains in older adults spanning the full cognitive spectrum. Its principal contribution is evidence that comparative effectiveness is domain-specific rather than uniformly favoring the most technologically immersive option.

Three modality-domain pairings emerge as the most defensible for practice. NI_ExG offers the most consistent benefit for global cognition and was the only modality with a statistically significant executive-function advantage. RE produced the only robust memory improvement whose PI also excluded the null, though this rests on a single direct comparison requiring multisite replication before clinical adoption. IVR_E achieved the highest SUCRA values and remarkable cross-study consistency across all domains, but wide PIs leave its apparent superiority hypothesis-generating. For memory, a cumulative training volume of approximately 1000 minutes or more appears to be a practical prerequisite for stable benefit.

Future research should prioritize adequately powered multisite RCTs with longer follow-up to assess durability and dementia-delay potential; direct head-to-head comparisons among active digital modalities; dose-response analyses, ideally via individual participant data meta-analysis; cognitive-status stratification to guide modality selection; greater methodological rigor, particularly allocation concealment and assessor blinding; and mechanistic studies linking neuroimaging and neurotrophic markers to domain-specific effects. Meanwhile, the low-cost scalability of RE and NI_ExG makes them practical options for rural and resource-limited settings and offers a modality-specific evidence base to inform clinical guidelines and digital health investment for aging populations.

## Supplementary material

10.2196/92764 Multimedia Appendix 1 The details of cognitive assessment tools of included studies.

10.2196/92764 Multimedia Appendix 2 Search strategy for each database.

10.2196/92764 Multimedia Appendix 3 Studies excluded at full-text assessment with reasons for exclusion.

10.2196/92764 Multimedia Appendix 4 Characteristics of included studies.

10.2196/92764 Multimedia Appendix 5 Results of network ranking analysis, consistency and inconsistency tests.

10.2196/92764 Multimedia Appendix 6 Pairwise meta-analysis.

10.2196/92764 Multimedia Appendix 7 Comparison-adjusted funnel plots and the Egger test.

10.2196/92764 Multimedia Appendix 8 Grading of Recommendations, Assessment, Development and Evaluation evidence rating.

10.2196/92764 Checklist 1 PRISMA checklist.

10.2196/92764 Checklist 2 PRISMA 2020 expanded checklist.

10.2196/92764 Checklist 3 PRISMA 2020 for abstracts checklist.

10.2196/92764 Checklist 4 PRISMA-S checklist.

## Abbreviations

CINeMA: Confidence in Network Meta-Analysis
GRADE: Grading of Recommendations, Assessment, Development and Evaluation
HKSJ: Hartung-Knapp-Sidik-Jonkman
IVR_E: immersive virtual reality exercise
MCI: mild cognitive impairment
NI: nonintervention
NI_ExG: nonimmersive exergame
NMA: network meta-analysis
PI: prediction interval
PRESS: Peer Review of Electronic Search Strategies
PRISMA: Preferred Reporting Items for Systematic Reviews and Meta-Analyses
PRISMA-NMA: Preferred Reporting Items for Systematic Reviews and Meta-Analyses extension for Network Meta-Analyses
PRISMA-S: PRISMA extension for Reporting Literature Searches in Systematic Reviews
RCT: randomized controlled trial
RE: remote exercise
RI: routine intervention
SMD: standardized mean difference
SUCRA: surface under the cumulative ranking curve
VR: virtual reality
VR_EC: virtual reality exercise combined with cognitive training
