# Supplementary material for: Digital Physical Exercise Interventions for Cognitive Functions in Older Adults: Systematic Review and Bayesian Network Meta-Analysis of Randomized Controlled Trials
Source: J Med Internet Res. 2026 Aug 18;28:e92764. doi: 10.2196/92764 (PMC13485154; doi:10.2196/92764)
Supplement: Supplementary file 2 [file jmir-v28-e92764-s001.docx]

**Table S1.** The measurement tools for different outcomes.

| **Cognitive domains** | **Measurement tools of interests** |
| --- | --- |
| Global cognition | Mini-Mental State Examination (MMSE); The Montreal cognitive assessment (MoCA); Hong Kong Montreal Cognitive Assessment (HK-MoCA); Addenbrooke’s Cognitive Examination Revised (ACE-R) |
| Executive function | Trial mark test (TMT-B, TMT-A); Stroop test; Flankers task; Clock test; Tower of London (TOL); Color Trails 2 Test (CT2); Eriksen Flanker Test (EFT) |
| Memory Function | Digit span test (backward, forward) (DS-F, DS-B); the Wechsler memory scale-Reversion (WMS-R); N-back task; Chinese Version of the California Verbal Learning Test (CVVLT); Loewenstein Occupational Therapy Cognitive Assessment-Geriatric (LOTCA-G) |
