# Supplementary material for: Digital Physical Exercise Interventions for Cognitive Functions in Older Adults: Systematic Review and Bayesian Network Meta-Analysis of Randomized Controlled Trials
Source: J Med Internet Res. 2026 Aug 18;28:e92764. doi: 10.2196/92764 (PMC13485154; doi:10.2196/92764)
Supplement: Supplementary file 3 [file jmir-v28-e92764-s002.docx]

**Pubmed (MEDLINE)**

**1. Digital Physical Exercise Interventions**

("Virtual Reality"[MeSH Terms]

OR "Virtual Reality Exposure Therapy"[MeSH Terms]

OR "Video Games"[MeSH Terms]

OR "Exergaming"[MeSH Terms]

OR "Exercise Therapy"[MeSH Terms]

OR "Telerehabilitation"[MeSH Terms]

OR "Telemedicine"[MeSH Terms]

OR "Mobile Applications"[MeSH Terms]

OR "Smartphone"[MeSH Terms]

OR "virtual reality"[Title/Abstract]

OR "VR exercise"[Title/Abstract]

OR "VR training"[Title/Abstract]

OR "VR-based"[Title/Abstract]

OR "augmented reality"[Title/Abstract]

OR "mixed reality"[Title/Abstract]

OR "head-mounted display"[Title/Abstract]

OR "HMD"[Title/Abstract]

OR "immersive"[Title/Abstract]

OR "exergam*"[Title/Abstract]

OR "exer-gam*"[Title/Abstract]

OR "active video game*"[Title/Abstract]

OR "video game*"[Title/Abstract]

OR "videogam*"[Title/Abstract]

OR "motion-based game*"[Title/Abstract]

OR "interactive game*"[Title/Abstract]

OR "serious game*"[Title/Abstract]

OR "gamif*"[Title/Abstract]

OR "Wii"[Title/Abstract]

OR "Nintendo"[Title/Abstract]

OR "Xbox"[Title/Abstract]

OR "Kinect"[Title/Abstract]

OR "PlayStation"[Title/Abstract]

OR "cybercycl*"[Title/Abstract]

OR "online exercise"[Title/Abstract]

OR "web-based exercise"[Title/Abstract]

OR "web-based physical"[Title/Abstract]

OR "internet-based exercise"[Title/Abstract]

OR "remote exercise"[Title/Abstract]

OR "tele-exercise"[Title/Abstract]

OR "telerehabilitation"[Title/Abstract]

OR "tele-rehabilitation"[Title/Abstract]

OR "app-based exercise"[Title/Abstract]

OR "application-based exercise"[Title/Abstract]

OR "mobile app exercise"[Title/Abstract]

OR "mobile health"[Title/Abstract]

OR "mhealth"[Title/Abstract]

OR "m-health"[Title/Abstract]

OR "ehealth"[Title/Abstract]

OR "e-health"[Title/Abstract]

OR "digital exercise"[Title/Abstract]

OR "digital physical activity"[Title/Abstract]

OR "digital fitness"[Title/Abstract]

OR "digital intervention"[Title/Abstract]

OR "technology-based exercise"[Title/Abstract]

OR "computer-based exercise"[Title/Abstract])

**2. Population**

("Aged"[MeSH Terms]

OR "Aged, 80 and over"[MeSH Terms]

OR "Aging"[MeSH Terms]

OR "Frail Elderly"[MeSH Terms]

OR "older adult*"[Title/Abstract]

OR "older people"[Title/Abstract]

OR "older person*"[Title/Abstract]

OR "elder*"[Title/Abstract]

OR "senior*"[Title/Abstract]

OR "geriatric*"[Title/Abstract]

OR "aged"[Title/Abstract]

OR "aging"[Title/Abstract]

OR "ageing"[Title/Abstract]

OR "60 years"[Title/Abstract]

OR "65 years"[Title/Abstract]

OR "70 years"[Title/Abstract]

OR "septuagenarian*"[Title/Abstract]

OR "octogenarian*"[Title/Abstract]

OR "nonagenarian*"[Title/Abstract])

**3. Outcomes**

("Cognition"[MeSH Terms]

OR "Executive Function"[MeSH Terms]

OR "Memory"[MeSH Terms]

OR "Attention"[MeSH Terms]

OR "Cognitive Dysfunction"[MeSH Terms]

OR "Dementia"[MeSH Terms]

OR "Alzheimer Disease"[MeSH Terms]

OR "Mild Cognitive Impairment"[MeSH Terms]

OR "Neuropsychological Tests"[MeSH Terms]

OR "Mental Status and Dementia Tests"[MeSH Terms]

OR "cognitive function*"[Title/Abstract]

OR "cognition"[Title/Abstract]

OR "cognitive"[Title/Abstract]

OR "global cognition"[Title/Abstract]

OR "executive function*"[Title/Abstract]

OR "executive control"[Title/Abstract]

OR "memory"[Title/Abstract]

OR "memory function*"[Title/Abstract]

OR "memory performance"[Title/Abstract]

OR "working memory"[Title/Abstract]

OR "attention"[Title/Abstract]

OR "processing speed"[Title/Abstract]

OR "information processing"[Title/Abstract]

OR "cognitive impairment"[Title/Abstract]

OR "cognitive decline"[Title/Abstract]

OR "cognitive performance"[Title/Abstract]

OR "neuropsychological"[Title/Abstract]

OR "mental status"[Title/Abstract]

OR "MMSE"[Title/Abstract]

OR "MoCA"[Title/Abstract]

OR "Trail Making"[Title/Abstract]

OR "Stroop"[Title/Abstract]

OR "digit span"[Title/Abstract])

4. **Study Design — Cochrane Highly Sensitive Search Strategy (2008 revision)**

("randomized controlled trial"[Publication Type]

OR "controlled clinical trial"[Publication Type]

OR "randomized"[Title/Abstract]

OR "randomised"[Title/Abstract]

OR "placebo"[Title/Abstract]

OR "clinical trials as topic"[MeSH Terms]

OR "randomly"[Title/Abstract]

OR "trial"[Title/Abstract])

NOT ("animals"[MeSH Terms] NOT "humans"[MeSH Terms])

5. **Filters**

AND ("2010/01/01"[Date - Publication] : "2026/04/01"[Date - Publication])

AND English[Language]

**Embase (via Embase.com)**

**1. Intervention — EMTREE Terms**

'virtual reality'/exp

OR 'virtual reality exposure therapy'/exp

OR 'exergame'/exp

OR 'video game'/exp

OR 'serious game'/de

OR 'gamification'/de

OR 'augmented reality'/exp

OR 'mixed reality'/de

OR 'head mounted display'/de

OR 'exercise therapy'/de

OR 'telerehabilitation'/exp

OR 'telemedicine'/exp

OR 'mobile application'/exp

OR 'smartphone'/exp

OR 'mobile health'/de

**2. Intervention — Free-Text Terms**

'virtual reality':ab,ti

OR 'VR exercise':ab,ti

OR 'VR training':ab,ti

OR 'VR-based':ab,ti

OR 'augmented reality':ab,ti

OR 'mixed reality':ab,ti

OR 'head-mounted display':ab,ti

OR 'HMD':ab,ti

OR 'immersive':ab,ti

OR exergam*:ab,ti

OR 'exer-gam*':ab,ti

OR 'active video game*':ab,ti

OR 'video game*':ab,ti

OR videogam*:ab,ti

OR 'motion-based game*':ab,ti

OR 'interactive game*':ab,ti

OR 'serious game*':ab,ti

OR gamif*:ab,ti

OR 'wii':ab,ti

OR 'nintendo':ab,ti

OR 'xbox':ab,ti

OR 'kinect':ab,ti

OR 'playstation':ab,ti

OR cybercycl*:ab,ti

OR 'online exercise':ab,ti

OR 'web-based exercise':ab,ti

OR 'web-based physical':ab,ti

OR 'internet-based exercise':ab,ti

OR 'remote exercise':ab,ti

OR 'tele-exercise':ab,ti

OR telerehabilitation:ab,ti

OR 'tele-rehabilitation':ab,ti

OR 'app-based exercise':ab,ti

OR 'application-based exercise':ab,ti

OR 'mobile app exercise':ab,ti

OR 'mobile health':ab,ti

OR mhealth:ab,ti

OR 'm-health':ab,ti

OR ehealth:ab,ti

OR 'e-health':ab,ti

OR 'digital exercise':ab,ti

OR 'digital physical activity':ab,ti

OR 'digital fitness':ab,ti

OR 'digital intervention':ab,ti

OR 'technology-based exercise':ab,ti

OR 'computer-based exercise':ab,ti

**3. Intervention Combined**

1 OR 2

**4. Population — EMTREE Terms**

'aged'/exp

OR 'aging'/exp

OR 'frail elderly'/de

**5. Population — Free-Text Terms**

'older adult*':ab,ti

OR 'older people':ab,ti

OR 'older person*':ab,ti

OR elder*:ab,ti

OR senior*:ab,ti

OR geriatric*:ab,ti

OR 'aged':ab,ti

OR aging:ab,ti

OR ageing:ab,ti

OR '60 years':ab,ti

OR '65 years':ab,ti

OR '70 years':ab,ti

OR septuagenarian*:ab,ti

OR octogenarian*:ab,ti

OR nonagenarian*:ab,ti

**6. Population Combined**

4 OR 5

**7. Outcomes — EMTREE Terms**

'cognition'/exp

OR 'executive function'/de

OR 'memory'/exp

OR 'attention'/exp

OR 'cognitive defect'/exp

OR 'dementia'/exp

OR 'Alzheimer disease'/exp

OR 'mild cognitive impairment'/exp

OR 'neuropsychological test'/exp

OR 'mental status examination'/de

OR 'cognitive function'/de

**8. Outcomes — Free-Text Terms**

'cognitive function*':ab,ti

OR cognition:ab,ti

OR cognitive:ab,ti

OR 'global cognition':ab,ti

OR 'executive function*':ab,ti

OR 'executive control':ab,ti

OR memory:ab,ti

OR 'memory function*':ab,ti

OR 'memory performance':ab,ti

OR 'working memory':ab,ti

OR attention:ab,ti

OR 'processing speed':ab,ti

OR 'information processing':ab,ti

OR 'cognitive impairment':ab,ti

OR 'cognitive decline':ab,ti

OR 'cognitive performance':ab,ti

OR neuropsychological:ab,ti

OR 'mental status':ab,ti

OR 'MMSE':ab,ti

OR 'MoCA':ab,ti

OR 'Trail Making':ab,ti

OR 'Stroop':ab,ti

OR 'digit span':ab,ti

**9. Outcomes Combined**

7 OR 8

**10. Study Design — Cochrane Embase RCT Filter (Embase.com version, 2023 revision)**

'crossover procedure':de

OR 'double-blind procedure':de

OR 'randomized controlled trial'/exp

OR 'controlled clinical trial'/de

OR 'single-blind procedure':de

OR 'randomization'/de

OR random*:ab,ti

OR factorial*:ab,ti

OR crossover*:ab,ti

OR (cross NEXT/1 over*):ab,ti

OR placebo*:ab,ti

OR (doubl* NEAR/1 blind*):ab,ti

OR (singl* NEAR/1 blind*):ab,ti

OR assign*:ab,ti

OR allocat*:ab,ti

OR volunteer*:ab,ti

**11. Animal Study Exclusion — Line 1**

(rat OR rats OR mouse OR mice OR swine OR porcine OR murine OR sheep

OR lambs OR pigs OR piglets OR rabbit OR rabbits OR cat OR cats OR dog

OR dogs OR cattle OR bovine OR monkey OR monkeys OR trout OR marmoset*):ti

AND 'animal experiment'/de

**12. Animal Study Exclusion — Line 2**

'animal experiment'/de NOT ('human experiment'/de OR 'human'/de)

**13. Animal Exclusion Combined**

11 OR 12

**14. Topic Sets Combined**

3 AND 6 AND 9 AND 10

**15 Exclude Animal Studies**

14 NOT 13

**16. Apply Limits**

15 AND [2010-2026]/py AND [english]/lim

**Web of Science Core Collection (SCI-EXPANDED and SSCI)**

**1. Population**

TS=("older adult*" OR "older people" OR "older person*" OR "elder*"

OR "senior*" OR "geriatric*" OR "aged" OR "aging" OR "ageing"

OR "frail elderly" OR "60 years" OR "65 years" OR "70 years"

OR "septuagenarian*" OR "octogenarian*" OR "nonagenarian*")

**2. Intervention**

TS=("virtual reality" OR "VR exercise" OR "VR training" OR "VR-based"

OR "augmented reality" OR "mixed reality" OR "head-mounted display"

OR "HMD" OR "immersive" OR "exergam*" OR "exer-gam*"

OR "active video game*" OR "video game*" OR "videogam*"

OR "motion-based game*" OR "interactive game*" OR "serious game*"

OR "gamif*" OR "gamified exercise" OR "interactive exercis*"

OR "Wii" OR "Nintendo" OR "Xbox" OR "Kinect" OR "PlayStation"

OR "cybercycl*" OR "online exercise" OR "web-based exercise"

OR "web-based physical" OR "internet-based exercise"

OR "remote exercise" OR "tele-exercise" OR "telerehabilitation"

OR "tele-rehabilitation" OR "app-based exercise"

OR "application-based exercise" OR "mobile app exercise"

OR "mobile health" OR "mhealth" OR "m-health" OR "ehealth"

OR "e-health" OR "digital exercise" OR "digital physical activity"

OR "digital fitness" OR "digital intervention" OR "digital train*"

OR "technology-based exercise" OR "computer-based exercise"

OR "smartphone")

**3. Outcomes**

TS=("cognitive function*" OR "cognition" OR "cognitive"

OR "cognitive performance" OR "cognitive outcome*"

OR "global cognition" OR "global cognitive"

OR "executive function*" OR "executive control"

OR "working memory" OR "cognitive flexibility"

OR "inhibitory control" OR "memory" OR "memory function*"

OR "memory performance" OR "episodic memory"

OR "information processing" OR "processing speed"

OR "attention" OR "cognitive impairment" OR "cognitive decline"

OR "neuropsychological" OR "mental status"

OR "MMSE" OR "MoCA" OR "Trail Making" OR "Stroop"

OR "digit span")

**4. Study Desig**n

TS=("randomized controlled trial" OR "randomised controlled trial"

OR "randomized" OR "randomised" OR "RCT" OR "randomly"

OR "random allocation" OR "randomly assigned" OR "placebo"

OR "controlled trial" OR "clinical trial" OR "trial")

**5. Combine**

1 AND 2 AND 3 AND 4

**6. Limits**

Timespan: 2010-01-01 to 2026-04-01

Languages: English

**the Cochrane Central Register of Controlled Trials (CENTRAL)**

**1. Intervention**

MeSH descriptor: [Virtual Reality] explode all trees

OR MeSH descriptor: [Virtual Reality Exposure Therapy] explode all trees

OR MeSH descriptor: [Video Games] explode all trees

OR MeSH descriptor: [Exercise Therapy] this term only

OR MeSH descriptor: [Telerehabilitation] explode all trees

OR MeSH descriptor: [Telemedicine] explode all trees

OR MeSH descriptor: [Mobile Applications] explode all trees

OR MeSH descriptor: [Smartphone] explode all trees

OR ("virtual reality" OR "VR exercise" OR "VR training" OR "VR-based"

OR "augmented reality" OR "mixed reality" OR "head-mounted display"

OR "HMD" OR "immersive" OR exergam* OR "exer-gam*"

OR "active video game*" OR "video game*" OR videogam*

OR "motion-based game*" OR "interactive game*" OR "serious game*"

OR gamif* OR "Wii" OR "Nintendo" OR "Xbox" OR "Kinect"

OR "PlayStation" OR cybercycl* OR "online exercise"

OR "web-based exercise" OR "web-based physical"

OR "internet-based exercise" OR "remote exercise" OR "tele-exercise"

OR "telerehabilitation" OR "tele-rehabilitation"

OR "app-based exercise" OR "application-based exercise"

OR "mobile app exercise" OR "mobile health" OR "mhealth"

OR "m-health" OR "ehealth" OR "e-health" OR "digital exercise"

OR "digital physical activity" OR "digital fitness"

OR "digital intervention" OR "technology-based exercise"

OR "computer-based exercise"):ti,ab,kw

**2. Population**

MeSH descriptor: [Aged] explode all trees

OR MeSH descriptor: [Aging] explode all trees

OR MeSH descriptor: [Frail Elderly] explode all trees

OR ("older adult*" OR "older people" OR "older person*" OR elder*

OR senior* OR geriatric* OR aged OR aging OR ageing

OR "frail elderly" OR "60 years" OR "65 years" OR "70 years"

OR septuagenarian* OR octogenarian* OR nonagenarian*):ti,ab,kw

**3. Outcomes**

MeSH descriptor: [Cognition] explode all trees

OR MeSH descriptor: [Executive Function] explode all trees

OR MeSH descriptor: [Memory] explode all trees

OR MeSH descriptor: [Attention] explode all trees

OR MeSH descriptor: [Cognitive Dysfunction] explode all trees

OR MeSH descriptor: [Dementia] explode all trees

OR MeSH descriptor: [Alzheimer Disease] explode all trees

OR MeSH descriptor: [Neuropsychological Tests] explode all trees

OR ("cognitive function*" OR cognition OR cognitive

OR "global cognition" OR "executive function*" OR "executive control"

OR memory OR "memory function*" OR "memory performance"

OR "working memory" OR attention OR "processing speed"

OR "information processing" OR "cognitive impairment"

OR "cognitive decline" OR "cognitive performance"

OR neuropsychological OR "mental status"

OR "MMSE" OR "MoCA" OR "Trail Making" OR "Stroop"

OR "digit span"):ti,ab,kw

**4. Combine**

1 AND 2 AND 3

**5. Limits**

Publication Year from 2010 to 2026

**PsycINFO (via EBSCOhost)**

**S1 Intervention — Thesaurus Terms**

DE "Virtual Reality"

OR DE "Augmented Reality"

OR DE "Computer Games"

OR DE "Telemedicine"

OR DE "Telehealth"

OR DE "Mobile Devices"

OR DE "Mobile Applications (Electronic)"

OR DE "Exercise"

OR DE "Physical Activity"

OR DE "Rehabilitation"

**S2 Intervention — Free-Text Terms**

TI ("virtual reality" OR "VR exercise" OR "VR training" OR "VR-based"

OR "augmented reality" OR "mixed reality" OR "head-mounted display"

OR "HMD" OR "immersive" OR exergam* OR "exer-gam*"

OR "active video game*" OR "video game*" OR videogam*

OR "motion-based game*" OR "interactive game*" OR "serious game*"

OR gamif* OR "Wii" OR "Nintendo" OR "Xbox" OR "Kinect"

OR "PlayStation" OR cybercycl* OR "online exercise"

OR "web-based exercise" OR "web-based physical"

OR "internet-based exercise" OR "remote exercise" OR "tele-exercise"

OR "telerehabilitation" OR "tele-rehabilitation"

OR "app-based exercise" OR "application-based exercise"

OR "mobile app exercise" OR "mobile health" OR "mhealth"

OR "m-health" OR "ehealth" OR "e-health" OR "digital exercise"

OR "digital physical activity" OR "digital fitness"

OR "digital intervention" OR "technology-based exercise"

OR "computer-based exercise" OR "smartphone")

OR AB ("virtual reality" OR "VR exercise" OR "VR training" OR "VR-based"

OR "augmented reality" OR "mixed reality" OR "head-mounted display"

OR "HMD" OR "immersive" OR exergam* OR "exer-gam*"

OR "active video game*" OR "video game*" OR videogam*

OR "motion-based game*" OR "interactive game*" OR "serious game*"

OR gamif* OR "Wii" OR "Nintendo" OR "Xbox" OR "Kinect"

OR "PlayStation" OR cybercycl* OR "online exercise"

OR "web-based exercise" OR "web-based physical"

OR "internet-based exercise" OR "remote exercise" OR "tele-exercise"

OR "telerehabilitation" OR "tele-rehabilitation"

OR "app-based exercise" OR "application-based exercise"

OR "mobile app exercise" OR "mobile health" OR "mhealth"

OR "m-health" OR "ehealth" OR "e-health" OR "digital exercise"

OR "digital physical activity" OR "digital fitness"

OR "digital intervention" OR "technology-based exercise"

OR "computer-based exercise" OR "smartphone")

**S3 Intervention Combined**

S1 OR S2

**S4 Population — Thesaurus Terms**

DE "Aging"

OR DE "Elder Care"

OR DE "Geriatrics"

OR DE "Geriatric Patients"

**S5 Population — Free-Text Terms**

TI ("older adult*" OR "older people" OR "older person*" OR elder*

OR senior* OR geriatric* OR aged OR aging OR ageing

OR "frail elderly" OR "60 years" OR "65 years" OR "70 years"

OR septuagenarian* OR octogenarian* OR nonagenarian*)

OR AB ("older adult*" OR "older people" OR "older person*" OR elder*

OR senior* OR geriatric* OR aged OR aging OR ageing

OR "frail elderly" OR "60 years" OR "65 years" OR "70 years"

OR septuagenarian* OR octogenarian* OR nonagenarian*)

**S6 Population Combined**

S4 OR S5

**S7 Outcomes — Thesaurus Terms**

DE "Cognition"

OR DE "Cognitive Ability"

OR DE "Executive Function"

OR DE "Memory"

OR DE "Short Term Memory"

OR DE "Long Term Memory"

OR DE "Working Memory"

OR DE "Attention"

OR DE "Cognitive Impairment"

OR DE "Dementia"

OR DE "Alzheimer's Disease"

OR DE "Mild Cognitive Impairment"

OR DE "Neuropsychological Assessment"

**S8 Outcomes — Free-Text Terms**

TI ("cognitive function*" OR cognition OR cognitive

OR "global cognition" OR "executive function*" OR "executive control"

OR memory OR "memory function*" OR "memory performance"

OR "working memory" OR attention OR "processing speed"

OR "information processing" OR "cognitive impairment"

OR "cognitive decline" OR "cognitive performance"

OR neuropsychological OR "mental status"

OR "MMSE" OR "MoCA" OR "Trail Making" OR "Stroop"

OR "digit span")

OR AB ("cognitive function*" OR cognition OR cognitive

OR "global cognition" OR "executive function*" OR "executive control"

OR memory OR "memory function*" OR "memory performance"

OR "working memory" OR attention OR "processing speed"

OR "information processing" OR "cognitive impairment"

OR "cognitive decline" OR "cognitive performance"

OR neuropsychological OR "mental status"

OR "MMSE" OR "MoCA" OR "Trail Making" OR "Stroop"

OR "digit span")

**S9 Outcomes Combined**

S7 OR S8

**S10 Study Design — RCT Filter (adapted from Harvard Library / ISSG for PsycINFO EBSCOhost)**

DE "Clinical Trials"

OR DE "Randomized Controlled Trials"

OR DE "Random Assignment"

OR DE "Placebo"

OR DE "Treatment Effectiveness Evaluation"

OR TI (random* OR trial OR placebo* OR "controlled trial"

OR "clinical trial" OR RCT)

OR AB (random* OR placebo* OR "randomly assigned"

OR "random allocation" OR "controlled trial" OR "clinical trial")

**S11 Combine Topic Sets**

S3 AND S6 AND S9 AND S10

**S12 Apply Limits**

S11

Limiters — Publication Year: 2010-2026

Limiters — Language: English

**CINAHL Plus with Full Text (via EBSCOhost)**

**S1 Intervention — CINAHL Headings**

MH "Virtual Reality+"

OR MH "Augmented Reality"

OR MH "Video Games"

OR MH "Therapeutic Exercise+"

OR MH "Telerehabilitation+"

OR MH "Telemedicine+"

OR MH "Mobile Applications"

OR MH "Smartphone"

**S2 Intervention — Free-Text Terms**

TI ("virtual reality" OR "VR exercise" OR "VR training" OR "VR-based"

OR "augmented reality" OR "mixed reality" OR "head-mounted display"

OR "HMD" OR "immersive" OR exergam* OR "exer-gam*"

OR "active video game*" OR "video game*" OR videogam*

OR "motion-based game*" OR "interactive game*" OR "serious game*"

OR gamif* OR "Wii" OR "Nintendo" OR "Xbox" OR "Kinect"

OR "PlayStation" OR cybercycl* OR "online exercise"

OR "web-based exercise" OR "web-based physical"

OR "internet-based exercise" OR "remote exercise" OR "tele-exercise"

OR "telerehabilitation" OR "tele-rehabilitation"

OR "app-based exercise" OR "application-based exercise"

OR "mobile app exercise" OR "mobile health" OR "mhealth"

OR "m-health" OR "ehealth" OR "e-health" OR "digital exercise"

OR "digital physical activity" OR "digital fitness"

OR "digital intervention" OR "technology-based exercise"

OR "computer-based exercise" OR "smartphone")

OR AB ("virtual reality" OR "VR exercise" OR "VR training" OR "VR-based"

OR "augmented reality" OR "mixed reality" OR "head-mounted display"

OR "HMD" OR "immersive" OR exergam* OR "exer-gam*"

OR "active video game*" OR "video game*" OR videogam*

OR "motion-based game*" OR "interactive game*" OR "serious game*"

OR gamif* OR "Wii" OR "Nintendo" OR "Xbox" OR "Kinect"

OR "PlayStation" OR cybercycl* OR "online exercise"

OR "web-based exercise" OR "web-based physical"

OR "internet-based exercise" OR "remote exercise" OR "tele-exercise"

OR "telerehabilitation" OR "tele-rehabilitation"

OR "app-based exercise" OR "application-based exercise"

OR "mobile app exercise" OR "mobile health" OR "mhealth"

OR "m-health" OR "ehealth" OR "e-health" OR "digital exercise"

OR "digital physical activity" OR "digital fitness"

OR "digital intervention" OR "technology-based exercise"

OR "computer-based exercise" OR "smartphone")

**S3 Intervention Combined**

S1 OR S2

**S4 Population — CINAHL Headings**

MH "Aged+"

OR MH "Aged, 80 and Over"

OR MH "Frail Elderly"

**S5 Population — Free-Text Terms**

TI ("older adult*" OR "older people" OR "older person*" OR elder*

OR senior* OR geriatric* OR aged OR aging OR ageing

OR "frail elderly" OR "60 years" OR "65 years" OR "70 years"

OR septuagenarian* OR octogenarian* OR nonagenarian*)

OR AB ("older adult*" OR "older people" OR "older person*" OR elder*

OR senior* OR geriatric* OR aged OR aging OR ageing

OR "frail elderly" OR "60 years" OR "65 years" OR "70 years"

OR septuagenarian* OR octogenarian* OR nonagenarian*)

**S6 Population Combined**

S4 OR S5

**S7 Outcomes — CINAHL Headings**

MH "Cognition+"

OR MH "Executive Function"

OR MH "Memory+"

OR MH "Attention+"

OR MH "Cognition Disorders+"

OR MH "Dementia+"

OR MH "Alzheimer's Disease"

OR MH "Mild Cognitive Impairment"

OR MH "Neuropsychological Tests+"

**S8 Outcomes — Free-Text Terms**

TI ("cognitive function*" OR cognition OR cognitive

OR "global cognition" OR "executive function*" OR "executive control"

OR memory OR "memory function*" OR "memory performance"

OR "working memory" OR attention OR "processing speed"

OR "information processing" OR "cognitive impairment"

OR "cognitive decline" OR "cognitive performance"

OR neuropsychological OR "mental status"

OR "MMSE" OR "MoCA" OR "Trail Making" OR "Stroop"

OR "digit span")

OR AB ("cognitive function*" OR cognition OR cognitive

OR "global cognition" OR "executive function*" OR "executive control"

OR memory OR "memory function*" OR "memory performance"

OR "working memory" OR attention OR "processing speed"

OR "information processing" OR "cognitive impairment"

OR "cognitive decline" OR "cognitive performance"

OR neuropsychological OR "mental status"

OR "MMSE" OR "MoCA" OR "Trail Making" OR "Stroop"

OR "digit span")

**S9 Outcomes Combined**

S7 OR S8

**S10 Study Design — Cochrane CINAHL RCT Filter (Glanville et al. 2019)**

(MH "Randomized Controlled Trials+"

OR MH "Double-Blind Studies"

OR MH "Single-Blind Studies"

OR MH "Random Assignment"

OR MH "Pretest-Posttest Design+"

OR MH "Cluster Sample"

OR TI (randomised OR randomized)

OR AB (random*)

OR TI (trial)

OR (MH "Sample Size" AND AB (assigned OR allocated OR control))

OR MH "Placebos"

OR PT "randomized controlled trial"

OR AB (control W5 group)

OR MH "Crossover Design"

OR MH "Comparative Studies"

OR AB (cluster W3 RCT))

NOT

((MH "Animals+" OR MH "Animal Studies" OR TI "animal model*")

NOT MH "Human")

**S11 Combine Topic Sets**

S3 AND S6 AND S9 AND S10

**S12 Apply Limits**

S11

Limiters — Publication Year: 2010-2026

Limiters — Language: English
