# Supplementary material for: Digital Physical Exercise Interventions for Cognitive Functions in Older Adults: Systematic Review and Bayesian Network Meta-Analysis of Randomized Controlled Trials
Source: J Med Internet Res. 2026 Aug 18;28:e92764. doi: 10.2196/92764 (PMC13485154; doi:10.2196/92764)
Supplement: Supplementary file 4 [file jmir-v28-e92764-s003.docx]

| **No** | **Reference** | **Reasons for exclusion** |
| --- | --- | --- |
| **1** | **1. Amjad I, Toor H, Niazi IK, et al. Xbox 360 Kinect Cognitive Games Improve Slowness, Complexity of EEG, and Cognitive Functions in Subjects with Mild Cognitive Impairment: A Randomized Control Trial. Games Health J. 2019;8(2):144-152. doi:10.1089/g4h.2018.0029** | **Participants not meeting criteria** |
| **2** | **1. An SJ, Jung S, Han A, et al. Effectiveness of functional mobile games to enhance attention in individuals with mild cognitive impairment: a study using the Montreal cognitive assessment. Yonsei Med J. 2025;66(11):731-742. doi:10.3349/ymj.2025.0029** | **Intervention not eligible** |
| **3** | **1. Anderson-Hanley C, Stark J, Wall KM, et al. The interactive physical and cognitive exercise system (iPACES™): effects of a 3-month in-home pilot clinical trial for mild cognitive impairment and caregivers. Clin Interv Aging. 2018;13:1565-1577. doi:10.2147/CIA.S160756** | **Not an RCT** |
| **4** | **1. Ballesteros S, Rieker JA, Mayas J, et al. Effects of multidomain versus single-domain training on executive control and memory in older adults: study protocol for a randomized controlled trial. Trials. 2020;21:404. doi:10.1186/s13063-020-04293-3** | **Not an RCT** |
| **5** | **1. Barcelos N, Shah N, Cohen K, et al. Aerobic and cognitive exercise (ACE) pilot study for older adults: executive function improves with cognitive challenge while exergaming. J Int Neuropsychol Soc. 2015;21(10):768-779. doi:10.1017/S1355617715001083** | **Not an RCT** |
| **6** | **1. Begde A, Alqurafi A, Pain MTG, Blenkinsop G, Wilcockson TDW, Hogervorst E. The effectiveness of home-based exergames training on cognition and balance in older adults: a comparative quasi-randomized study of two exergame interventions. Innov Aging. 2023;7(8):igad102. doi:10.1093/geroni/igad102** | **Not an RCT** |
| **7** | **1. Belleville S, Cuesta M, Bieler-Aeschlimann M, et al. Pre-frail older adults show improved cognition with StayFitLonger computerized home-based training: a randomized controlled trial. Geroscience. 2023;45(2):811-822. doi:10.1007/s11357-022-00674-5** | **Other reasons** |
| **8** | **1. Béraud-Peigné N, Maillot P, Perrot A. The user experience of an immersive and interactive wall exergame in older adults. Games Health J. 2023;12(3):220-227. doi:10.1089/g4h.2022.0075** | **Not an RCT** |
| **9** | **1. Cc H, Ps L, Wc H, et al. The effectiveness of a virtual reality-based tai chi exercise on cognitive and physical function in older adults with cognitive impairment. Dement Geriatr Cogn Disord. 2018;46(5-6). doi:10.1159/000494659** | **Outcome not eligible** |
| **10** | **1. de Souto Barreto P, Pothier K, Soriano G, et al. A web-based multidomain lifestyle intervention for older adults: the eMIND randomized controlled trial. J Prev Alzheimer’s Dis. 2021;8(2):142-150. doi:10.14283/jpad.2020.70** | **Other reasons** |
| **11** | **1. Dino MJS, Dion KW, Abadir PM, et al. Mixed reality technology for older adults: evaluating the impact of a novel virtual humanoid coach in a community-based physical exercise program in the Philippines. Health Inf J. 2024;30(3):14604582241267793. doi:10.1177/14604582241267793** | **Not an RCT** |
| **12** | **1. Eggenberger P, Annaheim S, Kündig KA, Rossi RM, Münzer T, de Bruin ED. Heart rate variability mainly relates to cognitive executive functions and improves through exergame training in older adults: a secondary analysis of a 6-month randomized controlled trial. Front Aging Neurosci. 2020;12:197. doi:10.3389/fnagi.2020.00197** | **Outcome not eligible** |
| **13** | **1. Eggenberger P, Schumacher V, Angst M, Theill N, de Bruin ED. Does multicomponent physical exercise with simultaneous cognitive training boost cognitive performance in older adults? A 6-month randomized controlled trial with a 1-year follow-up. Clin Interv Aging. 2015;10:1335-1349. doi:10.2147/CIA.S87732** | **Outcome not eligible** |
| **14** | **1. Ferreira S, Marmeleira J, del Pozo-Cruz J, et al. Acute effects of augmented reality exergames versus cycle ergometer on reaction time, visual attention, and verbal fluency in community older adults. Int J Environ Res Public Health. 2022;19(22):14667. doi:10.3390/ijerph192214667** | **Not an RCT** |
| **15** | **1. Ferreira S, Raimundo A, Pozo-Cruz J del, et al. Effects of multimodal exercise with augmented reality on cognition in community-dwelling older adults. J Am Med Dir Assoc. 2024;25(6). doi:10.1016/j.jamda.2024.01.023** | **Not an RCT** |
| **16** | **1. Garcia JA, Schoene D, Lord SR, Delbaere K, Valenzuela T, Navarro KF. A bespoke kinect stepping exergame for improving physical and cognitive function in older people: a pilot study. Games Health J. 2016;5(6):382-388. doi:10.1089/g4h.2016.0070** | **Not an RCT** |
| **17** | **1. Gunst M, De Meyere I, Willems H, Schoenmakers B. Effect of exergaming on wellbeing of residents in a nursing home: a single blinded intervention study. Aging Clin Exp Res. 2022;34(1):151-157. doi:10.1007/s40520-021-01903-1** | **Participants not meeting criteria** |
| **18** | **1. H N, S R, M S, Jp FG, J PG. Effect of virtual reality exercises on the cognitive status and dual motor task performance of the aging population. International Journal of Environmental Research and Public Health. 2021;18(15). doi:10.3390/ijerph18158005** | **Not an RCT** |
| **19** | **1. Hou HY, Li HJ. Effects of exergame and video game training on cognitive and physical function in older adults: A randomized controlled trial. Appl Ergon. 2022;101:103690. doi:10.1016/j.apergo.2022.103690** | **Outcome not eligible** |
| **20** | **1. Huang KT. Exergaming executive functions: an immersive virtual reality-based cognitive training for adults aged 50 and older. Cyberpsychol Behav Social Networking. 2020;23(3):143-149. doi:10.1089/cyber.2019.0269** | **Participants not meeting criteria** |
| **21** | **1. Kayama H, Okamoto K, Nishiguchi S, Yamada M, Kuroda T, Aoyama T. Effect of a kinect-based exercise game on improving executive cognitive performance in community-dwelling elderly: case control study. J Med Internet Res. 2014;16(2):e61. doi:10.2196/jmir.3108** | **Not an RCT** |
| **22** | **1. Kwan RY, Lee D, Lee PH, et al. Effects of an mHealth brisk walking intervention on increasing physical activity in older people with cognitive frailty: pilot randomized controlled trial. JMIR mHealth uHealth. 2020;8(7):e16596. doi:10.2196/16596** | **Outcome not eligible** |
| **23** | **1. Monteblanco Cavalcante M, Fraga I, Dalbosco B, et al. Exergame training-induced neuroplasticity and cognitive improvement in institutionalized older adults: a preliminary investigation. Physiology & Behavior. 2021;241:113589. doi:10.1016/j.physbeh.2021.113589** | **Not an RCT** |
| **24** | **1. Monteiro-Junior RS, Da Silva Figueiredo LF, Maciel-Pinheiro PDT, et al. Acute effects of exergames on cognitive function of institutionalized older persons: a single-blinded, randomized and controlled pilot study. Aging Clin Exp Res. 2017;29(3):387-394. doi:10.1007/s40520-016-0595-5** | **Outcome not eligible** |
| **25** | **1. Nuvolini RA, Silva KGD, Freitas TBD, Doná F, Torriani-Pasin C, Pompeu JE. Exergame-based program and conventional physiotherapy based on core areas of the European guideline similarly improve gait and cognition in people with parkinson’s disease: randomized clinical trial. Games Health J. 2025;14(5):358-368. doi:10.1089/g4h.2024.0116** | **Participants not meeting criteria** |
| **26** | **1. Ogawa EF, Huang H, Yu LF, et al. Effects of exergaming on cognition and gait in older adults at risk for falling. Med Sci Sports Exercise. 2020;52(3):754. doi:10.1249/MSS.0000000000002167** | **Not an RCT** |
| **27** | **1. Oliveira LM, Evangelista e Souza EH, Alves MR, et al. 2D Virtual Reality-Based Exercise Improves Spatial Navigation in Institutionalized Non-robust Older Persons: A Preliminary Data Report of a Single-Blind, Randomized, and Controlled Study. Front Neurol. 2021;11:609988. doi:10.3389/fneur.2020.609988** | **Outcome not eligible** |
| **28** | **1. Ordnung M, Hoff M, Kaminski E, Villringer A, Ragert P. No overt effects of a 6-week exergame training on sensorimotor and cognitive function in older adults. A preliminary investigation. Front Hum Neurosci. 2017;11:160. doi:10.3389/fnhum.2017.00160** | **Outcome not eligible** |
| **29** | **1. Peng HT, Tien CW, Lin PS, Peng HY, Song CY. Novel Mat Exergaming to Improve the Physical Performance, Cognitive Function, and Dual-Task Walking and Decrease the Fall Risk of Community-Dwelling Older Adults. Front Psychol. 2020;11:1620. doi:10.3389/fpsyg.2020.01620** | **Not an RCT** |
| **30** | **1. Phirom K, Kamnardsiri T, Sungkarat S. Beneficial effects of interactive physical-cognitive game-based training on fall risk and cognitive performance of older adults. Int J Environ Res Public Health. 2020;17(17):6079. doi:10.3390/ijerph17176079** | **Not an RCT** |
| **31** | **1. Rosenberg A, Untersteiner H, Guazzarini AG, et al. A digitally supported multimodal lifestyle program to promote brain health among older adults (the LETHE randomized controlled feasibility trial): study design, progress, and first results. Alzheimer’s Res Ther. 2024;16:252. doi:10.1186/s13195-024-01615-4** | **Not an RCT** |
| **32** | **1. Rozental-Iluz C, Zeilig G, Weingarden H, Rand D. Improving executive function deficits by playing interactive video-games: secondary analysis of a randomized controlled trial for individuals with chronic stroke. Eur J Phys Rehabil Med. 2016;52(4):508-515.** | **Participants not meeting criteria** |
| **33** | **1. Wais PE, Arioli M, Anguera-Singla R, Gazzaley A. Virtual reality video game improves high-fidelity memory in older adults. Sci Rep. 2021;11:2552. doi:10.1038/s41598-021-82109-3** | **Intervention not eligible** |
| **34** | **1. Wan C, Zhang Q, Qiu Y, et al. Effects of dual-task mode brain-computer interface based on motor imagery and virtual reality on balance and attention in patients with stroke: a randomized controlled pilot trial. J NeuroEng Rehabil. 2025;22:187. doi:10.1186/s12984-025-01730-9** | **Participants not meeting criteria** |
| **35** | **1. Werner C, Rosner R, Wiloth S, Lemke NC, Bauer JM, Hauer K. Time course of changes in motor-cognitive exergame performances during task-specific training in patients with dementia: identification and predictors of early training response. J NeuroEng Rehabil. 2018;15:100. doi:10.1186/s12984-018-0433-4** | **Not an RCT** |
| **36** | **1. Wu S, Jo EA, Ji H, et al. Exergaming Improves Executive Functions in Patients With Metabolic Syndrome: Randomized Controlled Trial. JMIR Serious Games. 2019;7(3):e13575. doi:10.2196/13575** | **Participants not meeting criteria** |
| **37** | **1. Yun SJ, Hyun SE, Oh BM, Seo HG. Fully immersive virtual reality exergames with dual-task components for patients with parkinson’s disease: a feasibility study. J Neuroeng Rehabil. 2023;20:92. doi:10.1186/s12984-023-01215-7** | **Not an RCT** |
| **38** | **1. Zhu YZ, Lin CF, Yang HL, Jin G, Chiu HL. Effects of exergaming on cognitive functions and loneliness of older adults with cognitive frailty. Int J Geriatr Psychiatry. 2023;38(6):e5944. doi:10.1002/gps.5944** | **Not an RCT** |
| **39** | **1. Zukowski LA, Shaikh FD, Haggard AV, Hamel RN. Acute effects of virtual reality treadmill training on gait and cognition in older adults: a randomized controlled trial. PLOS One. 2022;17(11):e0276989. doi:10.1371/journal.pone.0276989** | **Outcome not eligible** |
