# Supplementary material for: Digital Physical Exercise Interventions for Cognitive Functions in Older Adults: Systematic Review and Bayesian Network Meta-Analysis of Randomized Controlled Trials
Source: J Med Internet Res. 2026 Aug 18;28:e92764. doi: 10.2196/92764 (PMC13485154; doi:10.2196/92764)
Supplement: Supplementary file 5 [file jmir-v28-e92764-s004.docx]

**Multimedia Appendix 4. Characteristics of included studies.**

| **Study** | **Region** | **Participants (experiment vs control)** | | | | **Experimental group** | | | | | | **Comparison group(s)** | | **Cognitive test** |
| --- | --- | --- | --- | --- | --- | --- | --- | --- | --- | --- | --- | --- | --- | --- |
|  |  | **Sample size** | **Age** | **Male/female** | **Cognition** | **Intervention** | **Type** | **Total Dose** | **Length** | **Frequency** | **Duration** | **Intervention** | **Type** |  |
| **Adcock et al. (2020) [69]** | **Switzerland** | **15 vs 16** | **77.0 ± 6.4 vs 70.9 ± 5** | **5/10 vs 10/6** | **healthy** | **Multi-component sensory training, including Tai Chi-inspired exercises, dance, and gait-based cognitive games** | **NI_ExG** | **high** | **16 weeks** | **3 times per week** | **30-40 minutes** | **Standard care** | **NI** | **EF(Stroop test,TMT) MF(MFS-R)** |
| **Anderson-Hanley et al. (2012) [65]** | **United States** | **38 vs 41** | **75.7 ± 9.9 vs 81.6 ± 6.2** | **5/33 vs 12/29** | **healthy** | **Virtual reality cycling** | **IVR_E** | **high** | **3 months** | **5 times per week** | **45 minutes** | **Conventional stationary cycling exercise** | **RI** | **EF(Stroop test) MF(Digit span test)** |
| **Anderson-Hanley et al. (2012) [96]** | **United States** | **10 vs 10** | **72.8 ± 8.6 vs 73.7 ± 9.1** | **2/8 vs 0/10** | **healthy** | **Stationary bike combined with screen-based virtual environment and games, including virtual cycling routes, competitions, and interactive tasks** | **VR_EC** | **low** | **3 months** | **5 times per week** | **45 minutes** | **Traditional stationary bike exercise using the same equipment, without virtual environment or game content** | **RI** | **EF(Color Trails 2 Test,Stroop C Test) MF(Digit Span Backwards Test)** |
| **Anderson-Hanley et al. (2018) [66]** | **United States** | **7 vs 7 vs 20 vs 33** | **80.9 ± 12.3 vs 75.4 ± 9.8 vs NR vs NR** | **3/4 vs 4/3 vs NR vs NR** | **MCI** | **Cycling (low cognitive compliance: travel and high cognitive load: games)** | **IVR_E and NI_ExG** | **high** | **6 months** | **2–5 times per week** | **20-45 minutes** | **Game intervention only or conventional stationary cycling only** | **RI** | **EF(Stroop test) MF(Digit span test)** |
| **Bacha et al. (2018) [70]** | **Brazil** | **23 vs 23** | **71.0 ± 4.5 vs 66.5 ± 3.8** | **8/15 vs 4/19** | **healthy** | **Kinect Adventures four games (“Space Pop”, “20,000 Leaks”, “Reflex Ridge”, “River Rush”)** | **NI_ExG** | **low** | **7 weeks (14 sessions)** | **2 times per week** | **60 minutes** | **Conventional physical therapy, including warm-up, static/dynamic balance training, endurance training, strength training, motor coordination, stretching, and relaxation** | **RI** | **GC(MoCA)** |
| **Béraud-Peigné et al. (2024) [17]** | **France** | **19 vs 15** | **69.63 ± 5.3 vs 70.27 ± 5.8** | **7/12 vs 8/7** | **healthy** | **Exergames based on immersive interactive walls** | **IVR_E** | **high** | **12 weeks** | **2 times per week** | **60 minutes** | **Traditional walking and muscle strengthening training** | **RI** | **EF(Stroop test,TMT)** |
| **Callisaya et al. (2021) [93]** | **Australia** | **49 vs 44** | **72.9 ± 7.2 vs 72.8 ± 6.9** | **17/27 vs 22/27** | **Cognitive Impairment** | **Use the StandingTall program on your iPad for physical exercise (balance and strength training)** | **RE** | **high** | **6 months** | **weekly** | **120 minutes per week** | **Physical activity advice only** | **RI** | **EF(TMT)** |
| **Chan et al. (2019) [71]** | **Taiwan, China** | **31 vs 31** | **73.52 vs 72.32** | **2/29 vs 5/26** | **healthy** | **Hot Plus interactive health service system training, completing hand-eye coordination, upper/lower limb/trunk, and functional activity exercises through visual and auditory feedback** | **NI_ExG** | **low** | **8 weeks** | **3 times per week** | **30 minutes** | **Passive information activities on tablet, such as reading online news, puzzles, and spot-the-difference games** | **RI** | **GC(MoCA)** |
| **Choi & Lee (2019) [94]** | **South Korea** | **30 vs 30** | **77.27 ± 4.37 vs 75.37 ± 3.97** | **5/25 vs 4/26** | **MCI** | **Rowing** | **RE** | **low** | **6 weeks** | **2 times per week** | **60 minutes** | **Home-based exercises (including Williams flexion exercises, crunches, side leg lifts, and prone trunk extensions)** | **RI** | **GC(MoCA)** |
| **de Bruin et al. (2020) [72]** | **Switzerland** | **9 vs 8** | **86.1 ± 5.9 vs 90.3 ± 5.9** | **4/5 vs 3/5** | **Mild to Moderate Cognitive Impairment** | **Random resonance whole-body vibration + exergame dance training** | **NI_ExG** | **low** | **8 weeks** | **3 times per week** | **15 minutes** | **Low-intensity stochastic resonance whole-body vibration with passive trampoline training** | **RI** | **EF(TMT)** |
| **Delbroek et al. (2017) [97]** | **Belgium** | **10 vs 10** | **86.9 ± 5.6 vs 87.5 ± 6.6** | **2/8 vs 5/5** | **MCI** | **Nine balance, weight-bearing, memory, attention, and dual-task exercises using BioRescue platform to control tasks on screen via center-of-pressure movements** | **VR_EC** | **low** | **6 weeks** | **2 times per week** | **30 minutes** | **Routine care** | **NI** | **GC(MoCA)** |
| **Eggenberger et al. (2016) [73]** | **Switzerland** | **19 vs 14** | **72.8 ± 5.9 vs 77.8 ± 7.4** | **7/12 vs 5/9** | **healthy** | **Interactive video game dance** | **NI_ExG** | **low** | **8 weeks** | **3 times per week** | **30 minutes** | **Conventional balance and stretching training** | **RI** | **GC(MoCA) EF(Stroop test,TMT)** |
| **Ghous et al.(2024) [74]** | **Pakistan** | **14 VS 14** | **66.91±3.79 vs 66.72±2.91** | **8/6 vs 10/4** | **healthy** | **Using Nintendo Wii console, Wii Balance Board, and Wii Fit Plus software to perform games such as table tilting, ball balancing, tightrope walking, penguin slide, balance obstacle skiing, and jogging** | **NI_ExG** | **low** | **8 weeks** | **3 days per week** | **30 minutes/day for first 4 weeks, 40 minutes/day for last 4 weeks** | **Task-oriented circuit training including 10 exercises: sit-to-stand, self-sway, standing balance, step-ups, standing hamstring curl, tandem walk, Swiss ball squats, backward walking, side leg raises, and marching in place** | **RI** | **GC(MoCA)** |
| **Gomes et al. (2018) [75]** | **Brazil** | **15 vs 15** | **84 ± 6 vs 83 ± 5.87** | **NR** | **healthy** | **Exergames based on Nintendo Wii Fit Plus™** | **NI_ExG** | **low** | **7 weeks** | **2 times per week** | **50 minutes** | **Physical activity advice only** | **RI** | **GC(MoCA)** |
| **Gui et al. (2024) [76]** | **China** | **22 vs 27** | **63.32 ± 4.16 vs 62.93 ± 4.80** | **6/16 vs 4/23** | **MCI** | **Video game training on stationary bike (Yonder: The Cloud Catcher Chronic, YonderCCC), performing visual-cognitive tasks while cycling at 50–70% maximal heart rate** | **NI_ExG** | **high** | **4 months** | **2 times per week** | **60 minutes** | **Only daily life activities** | **NI** | **GC(MoCA) EF(TMT B-A) MF(Digit Span Forward & Backward)** |
| **Henrique et al. (2023) [77]** | **Brazil** | **10 vs 10** | **64.3 ± 2.0 vs 63.8 ± 2.4** | **all female** | **healthy** | **Combining exergames with physical training (limb training)** | **NI_ExG** | **low** | **6 weeks** | **2 times per week** | **60 minutes** | **Identical limb exercise training as experimental group without exergaming** | **RI** | **GC(MoCA)** |
| **Hou et al. (2023) [23]** | **China** | **41 vs 44 vs 43** | **67.41 ± 3.86 vs 68.84 ± 4.45 vs 67.44 ± 4.94** | **11/30 vs 15/29 vs 15/28** | **healthy** | **Exergames based on Nintendo Wii Sports (tennis, bowling, baseball, golf)** | **NI_ExG** | **high** | **16 weeks** | **3 times per week** | **60 minutes** | **Recumbent cycling or usual care only** | **RI or NI** | **EF(Stroop test,TMT) MF(2-back task)** |
| **Htut et al. (2018) [78]** | **Myanmar** | **21 vs 21 vs 21 vs 21** | **75.9 ± 5.65 vs 75.8 ± 4.89 vs 75.6 ± 5.33 vs 76.0 ± 5.22** | **13/8 vs 10/11 vs 12/9 vs 12/9** | **healthy** | **Exergames based on Xbox 360 (six games selected each time, involving upper and lower limb movement and balance training)** | **NI_ExG** | **low** | **8 weeks** | **3 times per week** | **30 minutes** | **Conventional physical exercise or cognitive exercise (including checkers, block games, and matching games) or usual care only** | **RI or NI** | **GC(MoCA)** |
| **Ip et al. (2025) [98]** | **Hong Kong, China** | **25 vs 30** | **71.96 ± 5.11 vs 77.23 ± 4.59** | **0/25 vs 6/24** | **MCI** | **VirCube VR CAVE training, including fire emergency handling, outdoor walking, balance games, and daily community activities** | **VR_EC** | **low** | **8–10 weeks (16 sessions)** | **2 times per week** | **45 minutes** | **Routine care** | **NI** | **GC(HK-MoCA) EF(TMT-A & TMT-B)** |
| **Karssemeijer et al. (2019) [18]** | **Netherlands** | **38 vs 38 vs 39** | **79.0 ± 6.9 vs 80.9 ± 6.1 vs 79.8 ± 6.5** | **20/18 vs 21/17 vs 21/18** | **Mild to Moderate Dementia** | **Exergames (cycling training combined with cognitive tasks using Bike Labyrinth)** | **NI_ExG** | **high** | **12 weeks** | **3 times per week** | **30-50 minutes** | **Cycling without cognitive tasks or relaxation and flexibility training** | **RI** | **EF(Stroop test,TMT)** |
| **Kwan et al. (2021) [99]** | **Hong Kong SAR, China** | **9 vs 8** | **73.0 ± 7.5 vs 77.5 ± 15.3** | **1/8 vs 1/7** | **MCI** | **Conduct cognitive games and cycling training simultaneously on a VR platform** | **VR_EC** | **low** | **8 weeks** | **2 times per week** | **30 minutes** | **Non-VR sequential motor-cognitive training (tablet-based cognitive games followed by cycling)** | **RI** | **GC(MoCA)** |
| **Kwan et al. (2024) [15]** | **Hong Kong SAR, China** | **146 vs 147** | **75.2 ± 7.1 vs 73.9 ± 6.6** | **36/110 vs 28/119** | **MCI** | **Simulate everyday activities such as shopping and cooking through VR games while riding a bicycle.** | **VR_EC** | **low** | **8 weeks** | **2 times per week** | **60 minutes** | **Standard care** | **NI** | **GC(MoCA) EF(Stroop test,TMT) MF(Digital span test)** |
| **Lee et al. (2023) [16]** | **South Korea** | **37 vs 40** | **70.3 ± 4.21 vs 69.88 ± 4.19** | **10/27 vs 9/31** | **SCD** | **Use the Silvia mobile app for daily video-assisted exercise** | **RE** | **low** | **12 weeks** | **5 times per week** | **15 minutes** | **Usual care only** | **NI** | **GC(MMSE)** |
| **Lee et al. (2024) [32]** | **South Korea** | **13 vs 14** | **70.64 ± 3.14 vs 71.50 ± 5.93** | **5/8 vs 5/9** | **SCD** | **Weight resistance training based on online videos** | **RE** | **high** | **8 weeks** | **3 times per week** | **40-50 minutes** | **Usual care only** | **NI** | **GC(MMSE)** |
| **Li et al. (2022) [33]** | **United States** | **23 vs 22 vs 24** | **74.4 ± 5.1 vs 74.5 ± 5.6 vs 74.9 ± 6.3** | **7/16 vs 7/15 vs 9/15** | **MCI** | **Online Tai Chi training based on the Zoom platform (combined with cognitive exercises)** | **RE** | **high** | **16 weeks** | **2 times per week** | **60 minutes** | **Identical Tai Chi training as experimental group without cognitive components or muscle stretching** | **RI** | **GC(MoCA) EF(Stroop test,TMT) MF(Digital span test)** |
| **Li et al. (2025) [92]** | **China** | **113 vs 113** | **73.12 ± 4.31 vs 72.29 ± 4.85** | **46/67 vs 53/60** | **MCI or Mild-to-Moderate Dementia** | **Nintendo Switch WarioWare: Move It! exergame** | **NI_ExG** | **high** | **12 weeks** | **2 times per week** | **60 minutes** | **Routine care** | **NI** | **GC(MoCA)** |
| **Liao et al. (2019) [62]** | **Taiwan, China** | **18 vs 16** | **75.5 ± 5.2 vs 73.1 ± 6.8** | **7/11 vs 4/12** | **MCI** | **VR-based motor cognition training (simplified 24-form Yang-style Tai Chi, resistance training, aerobic exercise, and functional tasks related to daily living activities; VR games such as riding the subway, cooking in the kitchen, working in a convenience store, etc.)** | **VR_EC** | **high** | **12 weeks** | **3 times per week** | **60 minutes** | **Conventional exercise (resistance, aerobic, and balance training) combined with cognitive training (e.g., walking while reciting poetry, naming flora/fauna while obstacle crossing, solving math problems during resistance training, drawing circles in air with alternating hands, Chinese character radical identification during chair stands)** | **RI** | **EF(Stroop test,TMT)** |
| **Liao et al. (2020) [63]** | **Taiwan, China** | **18 vs 16** | **75.5 ± 5.2 vs 73.1 ± 6.8** | **7/11 vs 4/12** | **MCI** | **VR-based motor cognition training (simplified 24-form Yang-style Tai Chi, resistance training, aerobic exercise, and functional tasks related to daily living activities; VR games such as riding the subway, cooking in the kitchen, working in a convenience store, etc.)** | **VR_EC** | **high** | **12 weeks** | **3 times per week** | **60 minutes** | **Conventional exercise (resistance, aerobic, and balance training) combined with cognitive training (e.g., walking while reciting poetry, naming flora/fauna while obstacle crossing, solving math problems during resistance training, drawing circles in air with alternating hands, Chinese character radical identification during chair stands)** | **RI** | **GC(MoCA) MF(CVVLT)** |
| **Liao et al. (2021) [79]** | **Taiwan, China** | **25 vs 21** | **79.6 ± 9.0 vs 83.8 ± 5.1** | **9/16 vs 6/15** | **healthy** | **Kinect-based motion-sensing games (resistance training, aerobic exercise, tai chi, and balance training)** | **NI_ExG** | **high** | **12 weeks** | **3 times per week** | **60 minutes** | **Conventional exercise (resistance, aerobic, and balance training)** | **RI** | **GC(MoCA) EF(TMT-B) MF(N-back task,CVVLT)** |
| **Liu et al. (2022) [80]** | **Taiwan, China** | **16 vs 17 vs 17** | **74.6 ± 6.1 vs 73.2 ± 6.3 vs 73.4 ± 6.5** | **4/12 vs 5/12 vs 6/11** | **MCI** | **Tai Chi Training** | **NI_ExG** | **high** | **12 weeks** | **3 times per week** | **50 minutes** | **Traditional Tai Chi training or usual care only** | **RI or NI** | **GC(MoCA) EF(TMT) MF(N-back task,CVVLT)** |
| **Maillot et al. (2012) [81]** | **France** | **15 vs 15** | **73.47 ± 4.10 vs 73.47 ± 3.00** | **4/11 vs 3/12** | **healthy** | **Exergames based on Nintendo Wii (involving various physical movements and cognitive judgment tasks, such as tennis, soccer headers on a balance board, ski jumps, and trampolines, etc.)** | **NI_ExG** | **high** | **12 weeks** | **2 times per week** | **60 minutes** | **Usual care only** | **NI** | **EF(TMT,SCWT)** |
| **Monteiro-Junior et al. (2017) [82]** | **Brazil** | **14 vs 15** | **85 ± 8 vs 86 ± 5** | **3/11 vs 3/12** | **Risk of Cognitive Decline** | **Exergames based on Nintendo Wii (rowing squats, penguin sliding, basic running, passing, heavy bag, and basic dancing)** | **NI_ExG** | **high** | **12-16 weeks** | **2 times per week** | **30-45 minutes** | **Same six exercises as experimental group without VR feedback** | **RI** | **GC(MMSE) EF(TMT-A) MF(DS-F)** |
| **Moreira et al. (2021) [83]** | **Brazil** | **32 vs 34** | **70.84 ± 4.53 vs 70.76 ± 5.60** | **9/23 vs 6/28** | **healthy** | **Exergames based on Xbox 360 Kinect (squats, lunges, boxing, sideways and forward/backward movements)** | **NI_ExG** | **high** | **12 weeks** | **3 times per week** | **50 minutes** | **Conventional multicomponent exercise (balance and cardiorespiratory exercises)** | **RI** | **GC(MMSE) EF(TMT)** |
| **Moret et al. (2022) [68]** | **Italy** | **38 vs 19** | **70.13 ± 3.73 vs 71.11 ± 3.72** | **9/29 vs 6/13** | **healthy** | **Kinect-based exergame “Dr. Kawashima's Body and Brain Exercises” (requires proprioception and eye-hand/leg coordination, including several cognitive tasks)** | **NI_ExG** | **low** | **2 weeks-3 weeks** | **3–4 times per week** | **45 minutes** | **Usual care only** | **NI** | **EF(TMT,SCWT) MF(N-back task,Digit span test) IP(SDMT)** |
| **Moret et al. (2024) [24]** | **Italy** | **28 vs 27 vs 29** | **70.89 ± 4.33 vs 70.75 ± 3.89 vs 69.03 ± 5.97** | **6/22 vs 8/19 vs 10/19** | **healthy** | **Exergames based on Xbox 360 Kinect (cycling, crossing the road, shopping, etc.)** | **NI_ExG** | **low** | **8 weeks** | **2–3 times per week** | **45 minutes** | **Attention training using MS-Rehab system or usual care only** | **RI or NI** | **MF(Digit span test) IP(SDMT)** |
| **Mrakic-Sposta et al. (2018) [100]** | **Italy** | **5 vs 5** | **72.00 ± 5.15 vs 74.60 ± 6.43** | **2/3 vs 2/3** | **MCI** | **VR-based motor and cognitive training (cycling, crossing the street, shopping, etc.)** | **VR_EC** | **low** | **6 weeks** | **3 times per week** | **40-45 minutes** | **Usual care only** | **NI** | **GC(MMSE) EF(TMT-A) AT(AMT)** |
| **Olyaei et al. (2022) [84]** | **Iran** | **27 vs 25** | **70.43 ± 5.24 vs 71.22 ± 5.82** | **9/18 vs 10/15** | **healthy** | **Balance game training using Wii Fit (involving physical and cognitive tasks, single-leg stretches, trunk twists, table tilts, penguin fishing, soccer headers, and tightrope walking)** | **NI_ExG** | **high** | **6 weeks** | **3 times per week** | **60 minutes** | **Balance training with concurrent cognitive tasks** | **RI** | **EF(TMT,Go/no go task)** |
| **Park & Park (2018) [85]** | **South Korea** | **39 vs 39** | **66.95 ± 4.10 vs 67.64 ± 4.55** | **20/19 vs 22/17** | **MCI** | **Play exergames (table tennis, sword fighting, and archery) using Nintendo Wii** | **NI_ExG** | **low** | **10 weeks** | **3 times per week** | **30 minutes** | **Cognitive training (attention, memory, and visuospatial abilities)** | **RI** | **EF(TMT-B) MF(Digit span test,RAVLT)** |
| **Park & Yim (2016) [67]** | **South Korea** | **36 vs 36** | **72.97 ± 2.98 vs 74.11 ± 2.88** | **3/33 vs 1/35** | **healthy** | **After 30 minutes of traditional exercise (side steps, walking in a line, walking backwards, etc.), do 20 minutes of 3D virtual reality kayaking training** | **IVR_E** | **low** | **6 weeks** | **2 times per week** | **50 minutes** | **30-minute conventional exercise (side stepping, tandem walking, backward walking)** | **RI** | **GC(MoCA)** |
| **Ramnath et al. (2021) [86]** | **South Africa** | **23 vs 22** | **70.8 ± 4.52 vs 74.14 ± 5.8** | **NR** | **Subjective Memory Complaints** | **Exergames based on Xbox Kinect Sports (bowling, boxing, track and field, table tennis, beach volleyball, soccer, etc.)** | **NI_ExG** | **high** | **12 weeks** | **2 times per week** | **60 minutes** | **Conventional low-intensity multimodal exercise (standing and seated exercises)** | **RI** | **GC(MMSE) MF(N-back task)** |
| **Recio-Rodriguez et al. (2022) [34]** | **Spain** | **81 vs 79** | **69.9 ± 3.6 vs 71.7 ± 4.2** | **31/50 vs 26/53** | **healthy** | **Exercise using the EVIDENT 3 app on your smartphone or smart bracelet** | **RE** | **-** | **3 months** | **daily** | **—** | **Physical activity advice only** | **RI** | **GC(MMSE) EF(Clock test)** |
| **Salisbury et al. (2024) [19]** | **United States** | **20 vs 11 vs 8** | **74.7 ± 7.6 vs 75.8 ± 8.9 vs 72.6 ± 4.3** | **9/11 vs 2/9 vs 2/6** | **SCD** | **Performing moderate-intensity cycling exercises in a VR environment while undergoing cognitive training with the BrainFitRx® cognitive rehabilitation training system** | **VR_EC** | **high** | **12 weeks** | **3 times per week** | **50 minutes** | **Moderate-intensity aerobic exercise on stationary bicycle or gentle stretching (seated static stretching and range-of-motion exercises)** | **RI** | **EF(Flanker task)** |
| **Santen et al. (2020) [20]** | **Netherlands** | **73 vs 39** | **79 ± 6 vs 79 ± 7** | **37/36 vs 23/16** | **Dementia** | **Interactive bicycle exergame** | **NI_ExG** | **high** | **6 months** | **at least 2 times per week** | **60 minutes** | **Standard care** | **NI** | **GC(MMSE) EF(TMT)** |
| **Schoene et al. (2013) [87]** | **Australia** | **15 vs 17** | **77.5 ± 4.5 vs 78.4 ± 4.5** | **NR** | **healthy** | **Step game using a computerized step mat system connected to a TV** | **NI_ExG** | **low** | **8 weeks** | **2–3 times per week** | **15-20 minutes** | **Usual care only** | **NI** | **EF(TMT)** |
| **Shake et al. (2018) [95]** | **United States** | **60 vs 45** | **73.59 ± 7.87 vs 73.22 ± 7.79** | **9/51 vs 6/39** | **healthy** | **Use the Bingocize® app, which combines bingo games, health education, and physical exercise** | **RE** | **high** | **10 weeks** | **2 times per week** | **60 minutes** | **Bingocize® application (Bingo games and health education) without exercise components** | **RI** | **EF(Flanker task)** |
| **Sturnieks et al. (2024) [25]** | **Australia** | **111 vs 118 vs 120** | **72.6 ± 5.7 vs 72.6 ± 5.5 vs 72.5 ± 5.5** | **NR** | **healthy** | **Using smart step pads for exergames** | **NI_ExG** | **high** | **6 months** | **at least 2 times per week** | **80 minutes** | **Touchscreen-based cognitive training or physical activity advice only** | **RI** | **GC(ACE-R) EF(TMT) MF(Digit span test)** |
| **Swinnen et al. (2021) [21]** | **Belgium** | **23 vs 22** | **84.7 ± 5.6 vs 85.3 ± 6.5** | **5/18 vs 5/17** | **Major Neurocognitive Disorder** | **Gait game based on Dividat Senso exergame equipment** | **NI_ExG** | **low** | **8 weeks** | **3 times per week** | **15 minutes** | **Music video viewing** | **RI** | **GC(MoCA)** |
| **Torpil et al. (2021) [88]** | **Turkey** | **30 VS 31** | **70.12±2.57 VS 70.30±2.73** | **11/19 VS 14/17** | **MCI** | **Full-body interaction using Microsoft Kinect with 3D motion capture, including four exergames: boxing, parkour, soccer penalty, and parachute** | **NI_ExG** | **high** | **12 weeks** | **2 times per week** | **45 minutes** | **Only conventional cognitive rehabilitation intervention** | **RI** | **MF(LOTCA-G)** |
| **Wang et al. (2021) [89]** | **Taiwan, China** | **10 vs 10** | **71.3 ± 5.33 vs 73.5 ± 5.66** | **3/7 vs 4/6** | **healthy** | **Dual-task training based on Kinect, walking on a treadmill while playing exergames (cognitive and motor games)** | **NI_ExG** | **high** | **12 weeks** | **3 times per week** | **60 minutes** | **Home-based multicomponent exercise (aerobic, strength, and balance training)** | **RI** | **EF(TMT,SCWT)** |
| **Wong et al. (2024) [90]** | **Hong Kong, China** | **101 vs 101** | **78.8 ± 7.8 vs 78.8 ± 7.8** | **-** | **MCI** | **Personalized motion-based video games (MBVGs) combined with PEO model and reference framework, targeting balance, handgrip strength, BMI, cognition, and short-term memory training, with psychological support** | **NI_ExG** | **low** | **18 weeks** | **approximately 1 time per week** | **60 minutes** | **Only daily life activities** | **NI** | **GC(HK-MoCA) MF(Digit Span Forward)** |
| **Wu et al. (2023) [91]** | **Korea, Republic of** | **13 vs 11** | **78.8 ± 4.8 vs 81.2 ± 4.4** | **5/8 vs 3/8** | **Mild-to-Moderate Dementia** | **ExerHeart device for running/jumping games “Alchemist's Treasure”, providing visual, auditory, and motor stimulation to enhance attention, executive function, and lower limb strength** | **NI_ExG** | **high** | **12 weeks** | **3 times per week** | **30–50 minutes** | **Commercial seated stationary bike (EGOJIN 704), resistance progressively increased according to gender; maintain 60–65% heart rate reserve, simulating general aerobic exercise** | **RI** | **EF(Eriksen Flanker Test)** |
| **Yu et al. (2025) [22]** | **Taiwan, China** | **46 vs 45** | **63.73 ± 4.48 vs 62.46 ± 4.77** | **8/38 vs 8/37** | **healthy** | **Kinect-based exergames (jumping, squatting, running in place, and sideways movement)** | **NI_ExG** | **low** | **10 weeks** | **2 times per week** | **50 minutes** | **Usual care only** | **NI** | **EF(TOL) MF(Digit span test)** |

NOTES: NR=not reported; VR=virtual reality; MCI=Mild Cognitive Impairment; SCD=Subjective Cognitive Decline; NI = non-intervention; RI = routine interventions; IVR_E = Immersive Virtual Reality Exercise; NI_ExG = Non-Immersive Exergame; RE = Remote exercise; VR_EC = virtual reality exercise combined with cognitive training; GC=Global cognition; EF=Executive function; MF=Memory function; TMT = Trail Making Test; TMT-A = Trail Making Test Part A; TMT-B = Trail Making Test Part B; MFS-R = Mental Function Screening-R; MoCA = Montreal Cognitive Assessment; MMSE = Mini-Mental State Examination; CVVLT = Chinese Version of Verbal Learning Test; SDMT = Symbol Digit Modalities Test; RAVLT = Rey Auditory Verbal Learning Test; ACE-R = Addenbrooke's Cognitive Examination-Revised; LOTCA-G = Loewenstein Occupational Therapy Cognitive Assessment-Geriatric; HK-MoCA = Hong Kong Montreal Cognitive Assessment; TOL = Tower of London.

**References**

15. Kwan RYC, Liu J, Sin OSK, et al. Effects of Virtual Reality Motor-Cognitive Training for Older People With Cognitive Frailty: Multicentered Randomized Controlled Trial. J Med Internet Res. 2024;26:e57809. PMID:38687508. doi:10.2196/57809.

16. Lee J, Kim J, Park A, et al. Efficacy of a Mobile-Based Multidomain Intervention to Improve Cognitive Function and Health-Related Outcomes Among Older Korean Adults With Subjective Cognitive Decline. J Alzheimers Dis. 2023;93(4):1551-1562. PMID:37182885. doi:10.3233/JAD-221299.

17. Béraud-Peigné N, Maillot P, Perrot A. The effects of a new immersive multidomain training on cognitive, dual-task and physical functions in older adults. Geroscience. 2024;46(2):1825-1841. PMID:37773385. doi:10.1007/s11357-023-00952-w.

18. Karssemeijer EGA, Aaronson JA, Bossers WJR, et al. The quest for synergy between physical exercise and cognitive stimulation via exergaming in people with dementia: a randomized controlled trial. Alzheimers Res Ther. 2019;11(1):3. PMID:30616641. doi:10.1186/s13195-018-0454-z.

19. Salisbury DL, Pituch KA, Yu F. The Effects of Exergame Telerehabilitation in Persons With Subjective Cognitive Decline. Gerontologist. 2024;64(6):gnae028. PMID:38489725. doi:10.1093/geront/gnae028.

20. van Santen J, Dröes RM, Twisk JWR, et al. Effects of Exergaming on Cognitive and Social Functioning of People with Dementia: A Randomized Controlled Trial. J Am Med Dir Assoc. 2020;21(12):1958-1967.e5. PMID:32527568. doi:10.1016/j.jamda.2020.04.018.

21. Swinnen N, Vandenbulcke M, de Bruin ED, et al. The efficacy of exergaming in people with major neurocognitive disorder residing in long-term care facilities: a pilot randomized controlled trial. Alzheimers Res Ther. 2021;13(1):70. PMID:33752744. doi:10.1186/s13195-021-00806-7.

22. Yu TC, Wu PT, Wu WL, et al. Effects of Exergame Training on Executive Function and Heart Rate Variability in Middle-Aged and Older Adults: A Randomized Controlled Study. Eur J Sport Sci. 2025;25(2):e12249. PMID:39545638. doi:10.1002/ejsc.12249.

23. Hou HY, Chen J, Hai L, et al. Effects of exergame and bicycle exercise intervention on blood pressure and executive function in older adults with hypertension: A three-group randomized controlled study. Exp Gerontol. 2023;173:112099. PMID:36709841. doi:10.1016/j.exger.2023.112099.

24. Moret B, Kolasinska AB, Nucci M, et al. Cognitive benefits of the attentional vs exergame training in older adults. Aging Ment Health. 2024;28(3):531-541. PMID:37395085. doi:10.1080/13607863.2023.2228220.

25. Sturnieks DL, Hicks C, Smith N, et al. Exergame and cognitive training for preventing falls in community-dwelling older people: a randomized controlled trial. Nat Med. 2024;30(1):98-105. PMID:38167683. doi:10.1038/s41591-023-02739-0.

32. Lee J, Lim BO, Byeon JY, Seok R. Effects of participation in an eight-week, online video body-weight resistance training on cognitive function and physical fitness in older adults: A randomized control trial. Geriatr Nurs. 2024;58:98-103. PMID:38788481. doi:10.1016/j.gerinurse.2024.05.001.

33. Li F, Harmer P, Fitzgerald K, Winters-Stone K. A cognitively enhanced online Tai Ji Quan training intervention for community-dwelling older adults with mild cognitive impairment: A feasibility trial. BMC Geriatr. 2022;22(1):76. PMID:35073860. doi:10.1186/s12877-021-02747-0.

34. Recio-Rodríguez JI, Gonzalez-Sanchez S, Tamayo-Morales O, et al. Changes in lifestyles, cognitive impairment, quality of life and activity day living after combined use of smartphone and smartband technology: a randomized clinical trial (EVIDENT-Age study). BMC Geriatr. 2022;22(1):782. PMID:36209015. doi:10.1186/s12877-022-03487-5.

62. Liao YY, Chen IH, Lin YJ, et al. Effects of Virtual Reality-Based Physical and Cognitive Training on Executive Function and Dual-Task Gait Performance in Older Adults With Mild Cognitive Impairment: A Randomized Control Trial. Front Aging Neurosci. 2019;11:162. PMID:31333454. doi:10.3389/fnagi.2019.00162.

63. Liao YY, Tseng HY, Lin YJ, et al. Using virtual reality-based training to improve cognitive function, instrumental activities of daily living and neural efficiency in older adults with mild cognitive impairment. Eur J Phys Rehabil Med. 2020;56(1):47-57. PMID:31448892. doi:10.23736/S1973-9087.19.05899-4.

65. Anderson-Hanley C, Arciero PJ, Brickman AM, et al. Exergaming and older adult cognition: a cluster randomized clinical trial. Am J Prev Med. 2012;42(2):109-119. PMID:22261206. doi:10.1016/j.amepre.2011.10.016.

66. Anderson-Hanley C, Barcelos NM, Zimmerman EA, et al. The Aerobic and Cognitive Exercise Study (ACES) for Community-Dwelling Older Adults With or At-Risk for Mild Cognitive Impairment (MCI): Neuropsychological, Neurobiological and Neuroimaging Outcomes of a Randomized Clinical Trial. Front Aging Neurosci. 2018;10:76. PMID:29662453. doi:10.3389/fnagi.2018.00076.

67. Park J, Yim J. A New Approach to Improve Cognition, Muscle Strength, and Postural Balance in Community-Dwelling Elderly with a 3-D Virtual Reality Kayak Program. Tohoku J Exp Med. 2016;238(1):1-8. PMID:26742586. doi:10.1620/tjem.238.1.

68. Moret B, Nucci M, Campana G. Effects of exergames on mood and cognition in healthy older adults: A randomized pilot study. Front Psychol. 2022;13:1018601. PMID:36341052. doi:10.3389/fpsyg.2022.1018601.

69. Adcock M, Fankhauser M, Post J, et al. Effects of an In-home Multicomponent Exergame Training on Physical Functions, Cognition, and Brain Volume of Older Adults: A Randomized Controlled Trial. Front Med (Lausanne). 2020;6:321. PMID:32039218. doi:10.3389/fmed.2019.00321.

70. Bacha JMR, Gomes GCV, de Freitas TB, et al. Effects of Kinect Adventures Games Versus Conventional Physical Therapy on Postural Control in Elderly People: A Randomized Controlled Trial. Games Health J. 2018;7(1):24-36. PMID:29239677. doi:10.1089/g4h.2017.0065.

71. Chan PT, Chang WC, Chiu HL, et al. Effect of interactive cognitive-motor training on eye-hand coordination and cognitive function in older adults. BMC Geriatr. 2019;19(1):27. PMID:30691404. doi:10.1186/s12877-019-1029-y.

72. de Bruin ED, Baur H, Brülhart Y, et al. Combining Stochastic Resonance Vibration With Exergaming for Motor-Cognitive Training in Long-Term Care: A Sham-Control Randomized Controlled Pilot Trial. Front Med (Lausanne). 2020;7:507155. PMID:33195349. doi:10.3389/fmed.2020.507155.

73. Eggenberger P, Wolf M, Schumann M, de Bruin ED. Exergame and Balance Training Modulate Prefrontal Brain Activity during Walking and Enhance Executive Function in Older Adults. Front Aging Neurosci. 2016;8:66. PMID:27148041. doi:10.3389/fnagi.2016.00066.

74. Ghous M, Masood Q, Nawaz Malik A, et al. Comparison of Nonimmersive Virtual Reality and Task-Oriented Circuit Training on Gait, Balance, and Cognition Among Elderly Population: A Single-Blind Randomized Control Trial. Games Health J. 2024;13(3):164-171. PMID:38512706. doi:10.1089/g4h.2022.0205.

75. Gomes GCV, Simões MDS, Lin SM, et al. Feasibility, safety, acceptability, and functional outcomes of playing Nintendo Wii Fit Plus for frail older adults: A randomized feasibility clinical trial. Maturitas. 2018;118:20-28. PMID:30415799. doi:10.1016/j.maturitas.2018.10.002.

76. Gui W, Cui X, Miao J, et al. The Effects of Simultaneous Aerobic Exercise and Video Game Training on Executive Functions and Brain Connectivity in Older Adults. Am J Geriatr Psychiatry. 2024;32(10):1244-1258. PMID:38763834. doi:10.1016/j.jagp.2024.04.009.

77. Henrique PPB, Perez FMP, Dorneles G, et al. Exergame and/or conventional training-induced neuroplasticity and cognitive improvement by engaging epigenetic and inflammatory modulation in elderly women: A randomized clinical trial. Physiol Behav. 2023;258:113996. PMID:36336183. doi:10.1016/j.physbeh.2022.113996.

78. Htut TZC, Hiengkaew V, Jalayondeja C, Vongsirinavarat M. Effects of physical, virtual reality-based, and brain exercise on physical, cognition, and preference in older persons: a randomized controlled trial. Eur Rev Aging Phys Act. 2018;15:10. PMID:30377465. doi:10.1186/s11556-018-0199-5.

79. Liao YY, Chen IH, Hsu WC, et al. Effect of exergaming versus combined exercise on cognitive function and brain activation in frail older adults: A randomised controlled trial. Ann Phys Rehabil Med. 2021;64(5):101492. PMID:33866030. doi:10.1016/j.rehab.2021.101492.

80. Liu CL, Cheng FY, Wei MJ, Liao YY. Effects of Exergaming-Based Tai Chi on Cognitive Function and Dual-Task Gait Performance in Older Adults With Mild Cognitive Impairment: A Randomized Control Trial. Front Aging Neurosci. 2022;14:761053. PMID:35281519. doi:10.3389/fnagi.2022.761053.

81. Maillot P, Perrot A, Hartley A. Effects of interactive physical-activity video-game training on physical and cognitive function in older adults. Psychol Aging. 2012;27(3):589-600. PMID:22082054. doi:10.1037/a0026268.

82. Monteiro-Junior RS, Figueiredo LFDS, Maciel-Pinheiro PT, et al. Virtual Reality-Based Physical Exercise With Exergames (PhysEx) Improves Mental and Physical Health of Institutionalized Older Adults. J Am Med Dir Assoc. 2017;18(5):454.e1-454.e9. PMID:28242194. doi:10.1016/j.jamda.2017.01.001.

83. Moreira NB, Rodacki ALF, Costa SN, et al. Perceptive-Cognitive and Physical Function in Prefrail Older Adults: Exergaming Versus Traditional Multicomponent Training. Rejuvenation Res. 2021;24(1):28-36. PMID:32669004. doi:10.1089/rej.2020.2302.

84. Olyaei G, Khanmohammadi R, Talebian S, et al. The effect of exergaming on cognition and brain activity in older adults: a motor-related cortical potential study. Physiol Behav. 2022;255:113941. PMID:36113576. doi:10.1016/j.physbeh.2021.113941.

85. Park JH, Park JH. Does cognition-specific computer training have better clinical outcomes than non-specific computer training? A single-blind, randomized controlled trial. Clin Rehabil. 2018;32(2):213-222. PMID:28726521. doi:10.1177/0269215517719951.

86. Ramnath U, Rauch L, Lambert EV, Kolbe-Alexander T. Efficacy of interactive video gaming in older adults with memory complaints: A cluster-randomized exercise intervention. PLoS One. 2021;16(5):e0252016. PMID:34043642. doi:10.1371/journal.pone.0252016.

87. Schoene D, Lord SR, Delbaere K, et al. A randomized controlled pilot study of home-based step training in older people using videogame technology. PLoS One. 2013;8(3):e57734. PMID:23483935. doi:10.1371/journal.pone.0057734.

88. Torpil B, Şahin S, Pekçetin S, Uyanık M. The Effectiveness of a Virtual Reality-Based Intervention on Cognitive Functions in Older Adults with Mild Cognitive Impairment: A Single-Blind, Randomized Controlled Trial. Games Health J. 2021;10(2):109-114. PMID:33058735. doi:10.1089/g4h.2020.0086.

89. Wang RY, Huang YC, Zhou JH, et al. Effects of Exergame-Based Dual-Task Training on Executive Function and Dual-Task Performance in Community-Dwelling Older People: A Randomized-Controlled Trial. Games Health J. 2021;10(5):347-354. PMID:34388046. doi:10.1089/g4h.2021.0057.

90. Wong AKC, Zhang MQ, Bayuo J, et al. The Effect of Young People-Assisted, Individualized, Motion-Based Video Games on Physical, Cognitive, and Social Frailty Among Community-Dwelling Older Adults With Frailty: Randomized Controlled Trial. JMIR Serious Games. 2024;12:e57352. PMID:39622701. doi:10.2196/57352.

91. Wu S, Ji H, Won J, et al. The Effects of Exergaming on Executive and Physical Functions in Older Adults With Dementia: Randomized Controlled Trial. J Med Internet Res. 2023;25:e39993. PMID:36881445. doi:10.2196/39993.

92. Li A, Qiang W, Li J, et al. Effectiveness of an exergame-based training program on physical and cognitive function in older adults with cognitive impairment: a randomized controlled trial in rural China. BMC Geriatr. 2025;25(1):892. PMID:41219720. doi:10.1186/s12877-025-06341-6.

93. Callisaya ML, Jayakody O, Vaidya A, et al. A novel cognitive-motor exercise program delivered via a tablet to improve mobility in older people with cognitive impairment—StandingTall Cognition and Mobility. Exp Gerontol. 2021;152:111434. PMID:34116124. doi:10.1016/j.exger.2021.111434.

94. Choi W, Lee S. The Effects of Virtual Kayak Paddling Exercise on Postural Balance, Muscle Performance, and Cognitive Function in Older Adults with Mild Cognitive Impairment: A Randomized Controlled Trial. J Aging Phys Act. 2019;27(4):861-870. PMID:30995133. doi:10.1123/japa.2018-0020.

95. Shake MC, Crandall KJ, Mathews RP, et al. Efficacy of Bingocize: A Game-Centered Mobile Application to Improve Physical and Cognitive Performance in Older Adults. Games Health J. 2018;7(4):253-261. PMID:30048171. doi:10.1089/g4h.2017.0139.

96. Anderson-Hanley C, Arciero PJ, Westen SC, et al. Neuropsychological benefits of stationary bike exercise and a cybercycle exergame for older adults with diabetes: an exploratory analysis. J Diabetes Sci Technol. 2012;6(4):849-857. PMID:22920811. doi:10.1177/193229681200600416.

97. Delbroek T, Vermeylen W, Spildooren J. The effect of cognitive-motor dual task training with the biorescue force platform on cognition, balance and dual task performance in institutionalized older adults: a randomized controlled trial. J Phys Ther Sci. 2017;29(7):1137-1143. PMID:28744033. doi:10.1589/jpts.29.1137.

98. Ip WK, Soar J, Fong K, et al. An Exploratory Study on Virtual Reality Technology for Fall Prevention in Older Adults with Mild Cognitive Impairment. Sensors (Basel). 2025;25(10):3123. PMID:40431915. doi:10.3390/s25103123.

99. Kwan RYC, Liu JYW, Fong KNK, et al. Feasibility and Effects of Virtual Reality Motor-Cognitive Training in Community-Dwelling Older People With Cognitive Frailty: Pilot Randomized Controlled Trial. JMIR Serious Games. 2021;9(3):e28400. PMID:34323635. doi:10.2196/28400.

100. Mrakic-Sposta S, Di Santo SG, Franchini F, et al. Effects of Combined Physical and Cognitive Virtual Reality-Based Training on Cognitive Impairment and Oxidative Stress in MCI Patients: A Pilot Study. Front Aging Neurosci. 2018;10:282. PMID:30319398. doi:10.3389/fnagi.2018.00282.
