# Supplementary material for: Digital Physical Exercise Interventions for Cognitive Functions in Older Adults: Systematic Review and Bayesian Network Meta-Analysis of Randomized Controlled Trials
Source: J Med Internet Res. 2026 Aug 18;28:e92764. doi: 10.2196/92764 (PMC13485154; doi:10.2196/92764)
Supplement: Supplementary file 6 [file jmir-v28-e92764-s005.docx]

**Network ranking analysis.**

**Table S1.** Results of network ranking analysis for global cognition.

| **Interventions** | **1** | **2** | **3** | **4** | **5** | **6** | **SUCRA** |
| --- | --- | --- | --- | --- | --- | --- | --- |
| **NI** | **0.000** | **0.000** | **0.005** | **0.036** | **0.191** | **1.000** | **0.046** |
| **RI** | **0.000** | **0.002** | **0.088** | **0.472** | **0.929** | **1.000** | **0.298** |
| **IVR_E** | **0.743** | **0.869** | **0.943** | **0.978** | **0.992** | **1.000** | **0.905** |
| **NI_ExG** | **0.151** | **0.745** | **0.961** | **0.999** | **1.000** | **1.000** | **0.771** |
| **RE** | **0.050** | **0.210** | **0.584** | **0.851** | **0.969** | **1.000** | **0.533** |
| **VR_EC** | **0.055** | **0.174** | **0.419** | **0.663** | **0.919** | **1.000** | **0.446** |

.

Notes: NI = non-intervention; RI = routine interventions; IVR_E = Immersive Virtual Reality Exercise; NI_ExG = Non-Immersive Exergame; RE = Remote exercise; VR_EC = virtual reality exercise combined with cognitive training; SUCRA = surface under cumulative ranking curve.

**Table S2.** Results of network ranking analysis for executive function.

| **Interventions** | **1** | **2** | **3** | **4** | **5** | **6** | **SUCRA** |
| --- | --- | --- | --- | --- | --- | --- | --- |
| **NI** | **0.000** | **0.004** | **0.024** | **0.093** | **0.283** | **1.000** | **0.081** |
| **RI** | **0.001** | **0.011** | **0.086** | **0.370** | **0.853** | **1.000** | **0.264** |
| **IVR_E** | **0.283** | **0.516** | **0.700** | **0.855** | **0.943** | **1.000** | **0.660** |
| **NI_ExG** | **0.104** | **0.391** | **0.748** | **0.962** | **0.998** | **1.000** | **0.641** |
| **RE** | **0.326** | **0.594** | **0.787** | **0.921** | **0.978** | **1.000** | **0.721** |
| **VR_EC** | **0.286** | **0.484** | **0.655** | **0.799** | **0.945** | **1.000** | **0.634** |

Notes: NI = non-intervention; RI = routine interventions; IVR_E = Immersive Virtual Reality Exercise; NI_ExG = Non-Immersive Exergame; RE = Remote exercise; VR_EC = virtual reality exercise combined with cognitive training; SUCRA = surface under cumulative ranking curve.

**Table S3.** Results of network ranking analysis for memory function.

| **Interventions** | **1** | **2** | **3** | **4** | **5** | **6** | **SUCRA** |
| --- | --- | --- | --- | --- | --- | --- | --- |
| **NI** | **0.001** | **0.015** | **0.082** | **0.323** | **0.829** | **1.000** | **0.250** |
| **RI** | **0.000** | **0.011** | **0.122** | **0.606** | **0.903** | **1.000** | **0.329** |
| **IVR_E** | **0.104** | **0.751** | **0.885** | **0.941** | **0.981** | **1.000** | **0.732** |
| **NI_ExG** | **0.011** | **0.226** | **0.842** | **0.973** | **0.997** | **1.000** | **0.610** |
| **RE** | **0.881** | **0.965** | **0.981** | **0.989** | **0.995** | **1.000** | **0.963** |
| **VR_EC** | **0.003** | **0.032** | **0.087** | **0.167** | **0.295** | **1.000** | **0.117** |

Notes: NI = non-intervention; RI = routine interventions; IVR_E = Immersive Virtual Reality Exercise; NI_ExG = Non-Immersive Exergame; RE = Remote exercise; VR_EC = virtual reality exercise combined with cognitive training; SUCRA = surface under cumulative ranking curve.

**Consistency test.**

**Table S4.** Global cognition consistency test.

| **Metric** | **Consistent Model** | **Inconsistent Model** |
| --- | --- | --- |
| **Posterior Mean Deviance (Dbar)** | **31.31** | **30.70** |
| **Effective Number of Parameters (pD)** | **16.14** | **17.13** |
| **Deviance Information Criterion (DIC)** | **47.45** | **47.83** |
| **ratio** | **1.04** | **1.02** |
| **I^2^** | **7%** | **6%** |

**Table S5.** Executive function cognition consistency test.

| **Metric** | **Consistent Model** | **Inconsistent Model** |
| --- | --- | --- |
| **Posterior Mean Deviance (Dbar)** | **35.30** | **35.46** |
| **Effective Number of Parameters (pD)** | **23.74** | **26.26** |
| **Deviance Information Criterion (DIC)** | **59.04** | **61.72** |
| **ratio** | **1.04** | **1.01** |
| **I^2^** | **7%** | **7%** |

**Table S6.** Memory function cognition consistency test.

| **Metric** | **Consistent Model** | **Inconsistent Model** |
| --- | --- | --- |
| **Posterior Mean Deviance (Dbar)** | **21.63** | **22.21** |
| **Effective Number of Parameters (pD)** | **16.67** | **18.13** |
| **Deviance Information Criterion (DIC)** | **38.30** | **40.34** |
| **ratio** | **0.98** | **1.01** |
| **I^2^** | **3%** | **5%** |

**Inconsistency test.**

**Figure S1.** Global cognition inconsistency test (node-splitting method). Abbreviations: NI = non-intervention; RI = routine interventions; IVR_E = Immersive Virtual Reality Exercise; NI_ExG = Non-Immersive Exergame; RE = Remote exercise; VR_EC = virtual reality exercise combined with cognitive training.

**Figure S2.** Executive function inconsistency test (node-splitting method). Abbreviations: NI = non-intervention; RI = routine interventions; IVR_E = Immersive Virtual Reality Exercise; NI_ExG = Non-Immersive Exergame; RE = Remote exercise; VR_EC = virtual reality exercise combined with cognitive training.

**Figure S3.** Memory function inconsistency test (node-splitting method). Abbreviations: NI = non-intervention; RI = routine interventions; IVR_E = Immersive Virtual Reality Exercise; NI_ExG = Non-Immersive Exergame; RE = Remote exercise; VR_EC = virtual reality exercise combined with cognitive training.
