# Supplementary material for: Digital Physical Exercise Interventions for Cognitive Functions in Older Adults: Systematic Review and Bayesian Network Meta-Analysis of Randomized Controlled Trials
Source: J Med Internet Res. 2026 Aug 18;28:e92764. doi: 10.2196/92764 (PMC13485154; doi:10.2196/92764)
Supplement: Supplementary file 7 [file jmir-v28-e92764-s006.docx]

**Global Cognition.**

**Figure S1.** Forest plot for testing overall heterogeneity in global cognition

**Figure S2.** Forest plot for testing overall heterogeneity in global cognition (excluding outliers)

**Figure S3.** Sensitivity analysis of global cognition

**Figure S4.** Global cognition Subgroup Analysis 1 (Cognitive Status)

**Figure S5.** Global cognition Subgroup Analysis 1 (Cognitive Status, excluding outliers)

**Figure S6.** Global cognition Subgroup Analysis 2 (Interventions)

**Figure S7.** Global cognition Subgroup Analysis 2 (Interventions, excluding outliers)

**Figure S8.** Global cognition Subgroup Analysis 3 (Total dose)

**Figure S9.** Global cognition Subgroup Analysis 3 (Total dose, excluding outliers)

**Executive Function.**

**Figure S10.** Forest plot for testing overall heterogeneity in executive function

**Figure S11.** Sensitivity analysis of executive function

**Figure S12.** Executive function Subgroup Analysis 1 (Cognitive Status)

**Figure S13.** Executive function Subgroup Analysis 2 (Interventions)

**Figure S14.** Executive function Subgroup Analysis 3 (Total dose)

**Memory Function.**

**Figure S15.** Forest plot for testing overall heterogeneity in memory function

**Figure S16.** Sensitivity analysis of memory function

**Figure S17.** Memory Function Subgroup Analysis 1 (Cognitive Status)

**Figure S18.** Memory Function Subgroup Analysis 2 (Interventions)

**Figure S19.** Memory Function Subgroup Analysis 3 (Total dose)

**References**

15. Kwan RYC, Liu J, Sin OSK, et al. Effects of Virtual Reality Motor-Cognitive Training for Older People With Cognitive Frailty: Multicentered Randomized Controlled Trial. J Med Internet Res. 2024;26:e57809. PMID:38687508. doi:10.2196/57809.

16. Lee J, Kim J, Park A, et al. Efficacy of a Mobile-Based Multidomain Intervention to Improve Cognitive Function and Health-Related Outcomes Among Older Korean Adults With Subjective Cognitive Decline. J Alzheimers Dis. 2023;93(4):1551-1562. PMID:37182885. doi:10.3233/JAD-221299.

17. Béraud-Peigné N, Maillot P, Perrot A. The effects of a new immersive multidomain training on cognitive, dual-task and physical functions in older adults. Geroscience. 2024;46(2):1825-1841. PMID:37773385. doi:10.1007/s11357-023-00952-w.

18. Karssemeijer EGA, Aaronson JA, Bossers WJR, et al. The quest for synergy between physical exercise and cognitive stimulation via exergaming in people with dementia: a randomized controlled trial. Alzheimers Res Ther. 2019;11(1):3. PMID:30616641. doi:10.1186/s13195-018-0454-z.

19. Salisbury DL, Pituch KA, Yu F. The Effects of Exergame Telerehabilitation in Persons With Subjective Cognitive Decline. Gerontologist. 2024;64(6):gnae028. PMID:38489725. doi:10.1093/geront/gnae028.

20. van Santen J, Dröes RM, Twisk JWR, et al. Effects of Exergaming on Cognitive and Social Functioning of People with Dementia: A Randomized Controlled Trial. J Am Med Dir Assoc. 2020;21(12):1958-1967.e5. PMID:32527568. doi:10.1016/j.jamda.2020.04.018.

21. Swinnen N, Vandenbulcke M, de Bruin ED, et al. The efficacy of exergaming in people with major neurocognitive disorder residing in long-term care facilities: a pilot randomized controlled trial. Alzheimers Res Ther. 2021;13(1):70. PMID:33752744. doi:10.1186/s13195-021-00806-7.

22. Yu TC, Wu PT, Wu WL, et al. Effects of Exergame Training on Executive Function and Heart Rate Variability in Middle-Aged and Older Adults: A Randomized Controlled Study. Eur J Sport Sci. 2025;25(2):e12249. PMID:39545638. doi:10.1002/ejsc.12249.

23. Hou HY, Chen J, Hai L, et al. Effects of exergame and bicycle exercise intervention on blood pressure and executive function in older adults with hypertension: A three-group randomized controlled study. Exp Gerontol. 2023;173:112099. PMID:36709841. doi:10.1016/j.exger.2023.112099.

24. Moret B, Kolasinska AB, Nucci M, et al. Cognitive benefits of the attentional vs exergame training in older adults. Aging Ment Health. 2024;28(3):531-541. PMID:37395085. doi:10.1080/13607863.2023.2228220.

25. Sturnieks DL, Hicks C, Smith N, et al. Exergame and cognitive training for preventing falls in community-dwelling older people: a randomized controlled trial. Nat Med. 2024;30(1):98-105. PMID:38167683. doi:10.1038/s41591-023-02739-0.

32. Lee J, Lim BO, Byeon JY, Seok R. Effects of participation in an eight-week, online video body-weight resistance training on cognitive function and physical fitness in older adults: A randomized control trial. Geriatr Nurs. 2024;58:98-103. PMID:38788481. doi:10.1016/j.gerinurse.2024.05.001.

33. Li F, Harmer P, Fitzgerald K, Winters-Stone K. A cognitively enhanced online Tai Ji Quan training intervention for community-dwelling older adults with mild cognitive impairment: A feasibility trial. BMC Geriatr. 2022;22(1):76. PMID:35073860. doi:10.1186/s12877-021-02747-0.

34. Recio-Rodríguez JI, Gonzalez-Sanchez S, Tamayo-Morales O, et al. Changes in lifestyles, cognitive impairment, quality of life and activity day living after combined use of smartphone and smartband technology: a randomized clinical trial (EVIDENT-Age study). BMC Geriatr. 2022;22(1):782. PMID:36209015. doi:10.1186/s12877-022-03487-5.

62. Liao YY, Chen IH, Lin YJ, et al. Effects of Virtual Reality-Based Physical and Cognitive Training on Executive Function and Dual-Task Gait Performance in Older Adults With Mild Cognitive Impairment: A Randomized Control Trial. Front Aging Neurosci. 2019;11:162. PMID:31333454. doi:10.3389/fnagi.2019.00162.

63. Liao YY, Tseng HY, Lin YJ, et al. Using virtual reality-based training to improve cognitive function, instrumental activities of daily living and neural efficiency in older adults with mild cognitive impairment. Eur J Phys Rehabil Med. 2020;56(1):47-57. PMID:31448892. doi:10.23736/S1973-9087.19.05899-4.

65. Anderson-Hanley C, Arciero PJ, Brickman AM, et al. Exergaming and older adult cognition: a cluster randomized clinical trial. Am J Prev Med. 2012;42(2):109-119. PMID:22261206. doi:10.1016/j.amepre.2011.10.016.

66. Anderson-Hanley C, Barcelos NM, Zimmerman EA, et al. The Aerobic and Cognitive Exercise Study (ACES) for Community-Dwelling Older Adults With or At-Risk for Mild Cognitive Impairment (MCI): Neuropsychological, Neurobiological and Neuroimaging Outcomes of a Randomized Clinical Trial. Front Aging Neurosci. 2018;10:76. PMID:29662453. doi:10.3389/fnagi.2018.00076.

67. Park J, Yim J. A New Approach to Improve Cognition, Muscle Strength, and Postural Balance in Community-Dwelling Elderly with a 3-D Virtual Reality Kayak Program. Tohoku J Exp Med. 2016;238(1):1-8. PMID:26742586. doi:10.1620/tjem.238.1.

68. Moret B, Nucci M, Campana G. Effects of exergames on mood and cognition in healthy older adults: A randomized pilot study. Front Psychol. 2022;13:1018601. PMID:36341052. doi:10.3389/fpsyg.2022.1018601.

69. Adcock M, Fankhauser M, Post J, et al. Effects of an In-home Multicomponent Exergame Training on Physical Functions, Cognition, and Brain Volume of Older Adults: A Randomized Controlled Trial. Front Med (Lausanne). 2020;6:321. PMID:32039218. doi:10.3389/fmed.2019.00321.

70. Bacha JMR, Gomes GCV, de Freitas TB, et al. Effects of Kinect Adventures Games Versus Conventional Physical Therapy on Postural Control in Elderly People: A Randomized Controlled Trial. Games Health J. 2018;7(1):24-36. PMID:29239677. doi:10.1089/g4h.2017.0065.

71. Chan PT, Chang WC, Chiu HL, et al. Effect of interactive cognitive-motor training on eye-hand coordination and cognitive function in older adults. BMC Geriatr. 2019;19(1):27. PMID:30691404. doi:10.1186/s12877-019-1029-y.

72. de Bruin ED, Baur H, Brülhart Y, et al. Combining Stochastic Resonance Vibration With Exergaming for Motor-Cognitive Training in Long-Term Care: A Sham-Control Randomized Controlled Pilot Trial. Front Med (Lausanne). 2020;7:507155. PMID:33195349. doi:10.3389/fmed.2020.507155.

73. Eggenberger P, Wolf M, Schumann M, de Bruin ED. Exergame and Balance Training Modulate Prefrontal Brain Activity during Walking and Enhance Executive Function in Older Adults. Front Aging Neurosci. 2016;8:66. PMID:27148041. doi:10.3389/fnagi.2016.00066.

74. Ghous M, Masood Q, Nawaz Malik A, et al. Comparison of Nonimmersive Virtual Reality and Task-Oriented Circuit Training on Gait, Balance, and Cognition Among Elderly Population: A Single-Blind Randomized Control Trial. Games Health J. 2024;13(3):164-171. PMID:38512706. doi:10.1089/g4h.2022.0205.

75. Gomes GCV, Simões MDS, Lin SM, et al. Feasibility, safety, acceptability, and functional outcomes of playing Nintendo Wii Fit Plus for frail older adults: A randomized feasibility clinical trial. Maturitas. 2018;118:20-28. PMID:30415799. doi:10.1016/j.maturitas.2018.10.002.

76. Gui W, Cui X, Miao J, et al. The Effects of Simultaneous Aerobic Exercise and Video Game Training on Executive Functions and Brain Connectivity in Older Adults. Am J Geriatr Psychiatry. 2024;32(10):1244-1258. PMID:38763834. doi:10.1016/j.jagp.2024.04.009.

77. Henrique PPB, Perez FMP, Dorneles G, et al. Exergame and/or conventional training-induced neuroplasticity and cognitive improvement by engaging epigenetic and inflammatory modulation in elderly women: A randomized clinical trial. Physiol Behav. 2023;258:113996. PMID:36336183. doi:10.1016/j.physbeh.2022.113996.

78. Htut TZC, Hiengkaew V, Jalayondeja C, Vongsirinavarat M. Effects of physical, virtual reality-based, and brain exercise on physical, cognition, and preference in older persons: a randomized controlled trial. Eur Rev Aging Phys Act. 2018;15:10. PMID:30377465. doi:10.1186/s11556-018-0199-5.

79. Liao YY, Chen IH, Hsu WC, et al. Effect of exergaming versus combined exercise on cognitive function and brain activation in frail older adults: A randomised controlled trial. Ann Phys Rehabil Med. 2021;64(5):101492. PMID:33866030. doi:10.1016/j.rehab.2021.101492.

80. Liu CL, Cheng FY, Wei MJ, Liao YY. Effects of Exergaming-Based Tai Chi on Cognitive Function and Dual-Task Gait Performance in Older Adults With Mild Cognitive Impairment: A Randomized Control Trial. Front Aging Neurosci. 2022;14:761053. PMID:35281519. doi:10.3389/fnagi.2022.761053.

81. Maillot P, Perrot A, Hartley A. Effects of interactive physical-activity video-game training on physical and cognitive function in older adults. Psychol Aging. 2012;27(3):589-600. PMID:22082054. doi:10.1037/a0026268.

82. Monteiro-Junior RS, Figueiredo LFDS, Maciel-Pinheiro PT, et al. Virtual Reality-Based Physical Exercise With Exergames (PhysEx) Improves Mental and Physical Health of Institutionalized Older Adults. J Am Med Dir Assoc. 2017;18(5):454.e1-454.e9. PMID:28242194. doi:10.1016/j.jamda.2017.01.001.

83. Moreira NB, Rodacki ALF, Costa SN, et al. Perceptive-Cognitive and Physical Function in Prefrail Older Adults: Exergaming Versus Traditional Multicomponent Training. Rejuvenation Res. 2021;24(1):28-36. PMID:32669004. doi:10.1089/rej.2020.2302.

84. Olyaei G, Khanmohammadi R, Talebian S, et al. The effect of exergaming on cognition and brain activity in older adults: a motor-related cortical potential study. Physiol Behav. 2022;255:113941. PMID:36113576. doi:10.1016/j.physbeh.2021.113941.

85. Park JH, Park JH. Does cognition-specific computer training have better clinical outcomes than non-specific computer training? A single-blind, randomized controlled trial. Clin Rehabil. 2018;32(2):213-222. PMID:28726521. doi:10.1177/0269215517719951.

86. Ramnath U, Rauch L, Lambert EV, Kolbe-Alexander T. Efficacy of interactive video gaming in older adults with memory complaints: A cluster-randomized exercise intervention. PLoS One. 2021;16(5):e0252016. PMID:34043642. doi:10.1371/journal.pone.0252016.

87. Schoene D, Lord SR, Delbaere K, et al. A randomized controlled pilot study of home-based step training in older people using videogame technology. PLoS One. 2013;8(3):e57734. PMID:23483935. doi:10.1371/journal.pone.0057734.

88. Torpil B, Şahin S, Pekçetin S, Uyanık M. The Effectiveness of a Virtual Reality-Based Intervention on Cognitive Functions in Older Adults with Mild Cognitive Impairment: A Single-Blind, Randomized Controlled Trial. Games Health J. 2021;10(2):109-114. PMID:33058735. doi:10.1089/g4h.2020.0086.

89. Wang RY, Huang YC, Zhou JH, et al. Effects of Exergame-Based Dual-Task Training on Executive Function and Dual-Task Performance in Community-Dwelling Older People: A Randomized-Controlled Trial. Games Health J. 2021;10(5):347-354. PMID:34388046. doi:10.1089/g4h.2021.0057.

90. Wong AKC, Zhang MQ, Bayuo J, et al. The Effect of Young People-Assisted, Individualized, Motion-Based Video Games on Physical, Cognitive, and Social Frailty Among Community-Dwelling Older Adults With Frailty: Randomized Controlled Trial. JMIR Serious Games. 2024;12:e57352. PMID:39622701. doi:10.2196/57352.

91. Wu S, Ji H, Won J, et al. The Effects of Exergaming on Executive and Physical Functions in Older Adults With Dementia: Randomized Controlled Trial. J Med Internet Res. 2023;25:e39993. PMID:36881445. doi:10.2196/39993.

92. Li A, Qiang W, Li J, et al. Effectiveness of an exergame-based training program on physical and cognitive function in older adults with cognitive impairment: a randomized controlled trial in rural China. BMC Geriatr. 2025;25(1):892. PMID:41219720. doi:10.1186/s12877-025-06341-6.

93. Callisaya ML, Jayakody O, Vaidya A, et al. A novel cognitive-motor exercise program delivered via a tablet to improve mobility in older people with cognitive impairment—StandingTall Cognition and Mobility. Exp Gerontol. 2021;152:111434. PMID:34116124. doi:10.1016/j.exger.2021.111434.

94. Choi W, Lee S. The Effects of Virtual Kayak Paddling Exercise on Postural Balance, Muscle Performance, and Cognitive Function in Older Adults with Mild Cognitive Impairment: A Randomized Controlled Trial. J Aging Phys Act. 2019;27(4):861-870. PMID:30995133. doi:10.1123/japa.2018-0020.

95. Shake MC, Crandall KJ, Mathews RP, et al. Efficacy of Bingocize: A Game-Centered Mobile Application to Improve Physical and Cognitive Performance in Older Adults. Games Health J. 2018;7(4):253-261. PMID:30048171. doi:10.1089/g4h.2017.0139.

96. Anderson-Hanley C, Arciero PJ, Westen SC, et al. Neuropsychological benefits of stationary bike exercise and a cybercycle exergame for older adults with diabetes: an exploratory analysis. J Diabetes Sci Technol. 2012;6(4):849-857. PMID:22920811. doi:10.1177/193229681200600416.

97. Delbroek T, Vermeylen W, Spildooren J. The effect of cognitive-motor dual task training with the biorescue force platform on cognition, balance and dual task performance in institutionalized older adults: a randomized controlled trial. J Phys Ther Sci. 2017;29(7):1137-1143. PMID:28744033. doi:10.1589/jpts.29.1137.

98. Ip WK, Soar J, Fong K, et al. An Exploratory Study on Virtual Reality Technology for Fall Prevention in Older Adults with Mild Cognitive Impairment. Sensors (Basel). 2025;25(10):3123. PMID:40431915. doi:10.3390/s25103123.

99. Kwan RYC, Liu JYW, Fong KNK, et al. Feasibility and Effects of Virtual Reality Motor-Cognitive Training in Community-Dwelling Older People With Cognitive Frailty: Pilot Randomized Controlled Trial. JMIR Serious Games. 2021;9(3):e28400. PMID:34323635. doi:10.2196/28400.

100. Mrakic-Sposta S, Di Santo SG, Franchini F, et al. Effects of Combined Physical and Cognitive Virtual Reality-Based Training on Cognitive Impairment and Oxidative Stress in MCI Patients: A Pilot Study. Front Aging Neurosci. 2018;10:282. PMID:30319398. doi:10.3389/fnagi.2018.00282.
