# Supplementary material for: Digital Physical Exercise Interventions for Cognitive Functions in Older Adults: Systematic Review and Bayesian Network Meta-Analysis of Randomized Controlled Trials
Source: J Med Internet Res. 2026 Aug 18;28:e92764. doi: 10.2196/92764 (PMC13485154; doi:10.2196/92764)
Supplement: Supplementary file 8 [file jmir-v28-e92764-s007.docx]

**Figure S1.** Global cognition corrected comparison funnel plot

**Figure S2.** Executive function corrected comparison funnel plot

**Figure S3.** Memory function corrected comparison funnel plot
