# Supplementary material for: Digital Physical Exercise Interventions for Cognitive Functions in Older Adults: Systematic Review and Bayesian Network Meta-Analysis of Randomized Controlled Trials
Source: J Med Internet Res. 2026 Aug 18;28:e92764. doi: 10.2196/92764 (PMC13485154; doi:10.2196/92764)
Supplement: Supplementary file 9 [file jmir-v28-e92764-s008.docx]

**Table S1. GRADE Summary of Findings tables**

Certainty of evidence for each network comparison across the three cognitive outcomes (global cognition, executive function, and memory function) in older adults aged ≥60 years. Tables were generated with GRADEpro GDT (GRADE Working Group). Effect sizes are network meta-analysis estimates expressed as standardised mean differences (SMD); certainty ratings were derived from the CINeMA (Confidence in Network Meta-Analysis) framework.

**GRADE Working Group grades of evidence**

High certainty: We are very confident that the true effect lies close to that of the estimate of the effect.

Moderate certainty: We are moderately confident in the effect estimate; the true effect is likely to be close to the estimate of the effect, but there is a possibility that it is substantially different.

Low certainty: Our confidence in the effect estimate is limited; the true effect may be substantially different from the estimate of the effect.

Very low certainty: We have very little confidence in the effect estimate; the true effect is likely to be substantially different from the estimate of effect.

*CI: confidence interval; SMD: standardised mean difference; RCT: randomised controlled trial.*

**Summary of findings: IVR_E (immersive virtual reality exercise) compared to NI (non-intervention) for cognitive function in older adults aged ≥60 years**

| **Outcomes** | **Anticipated absolute effects* (95% CI)** | **№ of participants (studies)** | **Certainty of the evidence (GRADE)** |
| --- | --- | --- | --- |
| **Global cognition** | SMD 0.74 SD higher (0.11 higher to 1.36 higher) | (0 RCTs) | ⨁◯◯◯ **Very low**  (a, b) |
| **Executive function** | SMD 0.44 SD higher (0.19 lower to 1.08 higher) | (0 RCTs) | ⨁◯◯◯ **Very low**  (a, b, c, d) |
| **Memory function** | SMD 0.54 SD higher (0.22 lower to 1.29 higher) | (0 RCTs) | ⨁◯◯◯ **Very low**  (a, c, e) |

** The risk in the intervention group (and its 95% confidence interval) is based on the assumed risk in the comparison group and the relative effect of the intervention (and its 95% CI).*

**Explanations**

a. Risk of bias: Most contributing trials were at unclear or high risk of bias, chiefly owing to unclear allocation concealment and unblinded outcome assessment. In addition, this comparison is a network estimate informed only by indirect evidence, with no direct head-to-head trials.

b. Inconsistency – very serious: Substantial statistical heterogeneity together with incoherence between direct and indirect estimates in the network.

c. Imprecision – serious: The 95% confidence interval is wide and crosses the null value (no effect).

d. Publication bias: Funnel-plot asymmetry and a significant Egger test for this outcome raised concern about reporting bias.

e. Substantial statistical heterogeneity among the studies informing this estimate.

**Summary of findings: IVR_E (immersive virtual reality exercise) compared to RI (routine intervention) for cognitive function in older adults aged ≥60 years**

| **Outcomes** | **Anticipated absolute effects* (95% CI)** | **№ of participants (studies)** | **Certainty of the evidence (GRADE)** |
| --- | --- | --- | --- |
| **Global cognition** | SMD 0.54 SD higher (0.00 to 1.09 higher) | 106 (2 RCTs) | ⨁◯◯◯ **Very low**  (a, b) |
| **Executive function** | SMD 0.26 SD higher (0.25 lower to 0.78 higher) | 159 (4 RCTs) | ⨁◯◯◯ **Very low**  (a, c, d) |
| **Memory function** | SMD 0.46 SD higher (0.20 lower to 1.11 higher) | 97 (2 RCTs) | ⨁◯◯◯ **Very low**  (a, c, e) |

** The risk in the intervention group (and its 95% confidence interval) is based on the assumed risk in the comparison group and the relative effect of the intervention (and its 95% CI).*

**Explanations**

a. Most contributing trials were at unclear or high risk of bias, chiefly owing to unclear allocation concealment and unblinded outcome assessment (Cochrane Risk of Bias tool, version 1).

b. Substantial statistical heterogeneity together with incoherence between direct and indirect estimates in the network.

c. The 95% confidence interval is wide and crosses the null value (no effect).

d. Funnel-plot asymmetry and a significant Egger test for executive function raised concern about reporting bias.

e. Substantial statistical heterogeneity among the studies informing this estimate.

**Summary of findings: NI_ExG (non-immersive exergame) compared to NI (non-intervention) for cognitive function in older adults aged ≥60 years**

| **Outcomes** | **Anticipated absolute effects* (95% CI)** | **№ of participants (studies)** | **Certainty of the evidence (GRADE)** |
| --- | --- | --- | --- |
| **Global cognition** | SMD 0.51 SD higher (0.26 higher to 0.78 higher) | 636 (6 RCTs) | ⨁◯◯◯ **Very low**  (a, b) |
| **Executive function** | SMD 0.39 SD higher (0.04 higher to 0.76 higher) | 381 (7 RCTs) | ⨁◯◯◯ **Very low**  (a, c, d) |
| **Memory function** | SMD 0.29 SD higher (0.07 lower to 0.65 higher) | 526 (7 RCTs) | ⨁◯◯◯ **Very low**  (a, c, e) |

** The risk in the intervention group (and its 95% confidence interval) is based on the assumed risk in the comparison group and the relative effect of the intervention (and its 95% CI).*

**Explanations**

a. Most contributing trials were at unclear or high risk of bias, chiefly owing to unclear allocation concealment and unblinded outcome assessment (Cochrane RoB tool, version 1).

b. Substantial statistical heterogeneity together with incoherence between direct and indirect estimates in the network.

c. Substantial statistical heterogeneity among the studies informing this estimate.

d. Funnel-plot asymmetry and a significant Egger test for executive function suggest possible reporting bias.

e. The 95% confidence interval is wide and crosses the null value (no effect).

**Summary of findings: NI_ExG (non-immersive exergame) compared to RI (routine intervention) for cognitive function in older adults aged ≥60 years**

| **Outcomes** | **Anticipated absolute effects* (95% CI)** | **№ of participants (studies)** | **Certainty of the evidence (GRADE)** |
| --- | --- | --- | --- |
| **Global cognition** | SMD 0.32 SD higher (0.12 higher to 0.53 higher) | 670 (12 RCTs) | ⨁⨁◯◯ **Low**  (a, b) |
| **Executive function** | SMD 0.22 SD higher (0.06 lower to 0.51 higher) | 648 (11 RCTs) | ⨁◯◯◯ **Very low**  (a, b, c, d) |
| **Memory function** | SMD 0.21 SD higher (0.11 lower to 0.54 higher) | 477 (6 RCTs) | ⨁◯◯◯ **Very low**  (a, b, c) |

** The risk in the intervention group (and its 95% confidence interval) is based on the assumed risk in the comparison group and the relative effect of the intervention (and its 95% CI).*

**Explanations**

a. Most contributing trials were at unclear or high risk of bias, chiefly owing to unclear allocation concealment and unblinded outcome assessment (Cochrane RoB tool, version 1).

b. Substantial statistical heterogeneity across the contributing studies.

c. The 95% confidence interval is wide and crosses the null value (no effect).

d. Funnel-plot asymmetry and a significant Egger test for this outcome raised concern about reporting bias.

**Summary of findings: RE (remote exercise) compared to NI (non-intervention) for cognitive function in older adults aged ≥60 years**

| **Outcomes** | **Anticipated absolute effects* (95% CI)** | **№ of participants (studies)** | **Certainty of the evidence (GRADE)** |
| --- | --- | --- | --- |
| **Global cognition** | SMD 0.35 SD higher (0.05 lower to 0.77 higher) | 104 (2 RCTs) | ⨁◯◯◯ **Very low**  (a, b, c) |
| **Executive function** | SMD 0.48 SD higher (0.07 lower to 1.06 higher) | 32 (1 RCT) | ⨁◯◯◯ **Very low**  (a, c, d, e) |
| **Memory function** | SMD 1.30 SD higher (0.15 higher to 2.44 higher) | (0 RCTs) | ⨁⨁◯◯ **Low**  (d, f) |

** The risk in the intervention group (and its 95% confidence interval) is based on the assumed risk in the comparison group and the relative effect of the intervention (and its 95% CI).*

**Explanations**

a. Most contributing trials were at unclear or high risk of bias, chiefly owing to unclear allocation concealment and unblinded outcome assessment (Cochrane Risk of Bias tool, version 1).

b. Substantial statistical heterogeneity together with incoherence between direct and indirect estimates in the network.

c. The 95% confidence interval is wide and crosses the null value (no effect).

d. Substantial statistical heterogeneity among the studies informing this estimate.

e. Funnel-plot asymmetry and a significant Egger test for executive function raised concern about reporting bias.

f. Most contributing trials were at unclear or high risk of bias, chiefly owing to unclear allocation concealment and unblinded outcome assessment. In addition, this comparison is a network estimate informed only by indirect evidence, with no direct head-to-head trials.

**Summary of findings: RE (remote exercise) compared to RI (routine intervention) for cognitive function in older adults aged ≥60 years**

| **Outcomes** | **Anticipated absolute effects* (95% CI)** | **№ of participants (studies)** | **Certainty of the evidence (GRADE)** |
| --- | --- | --- | --- |
| **Global cognition** | SMD 0.15 SD higher (0.20 lower to 0.53 higher) | 267 (3 RCTs) | ⨁◯◯◯ **Very low**  (a, b, c) |
| **Executive function** | SMD 0.31 SD higher (0.14 lower to 0.79 higher) | 369 (4 RCTs) | ⨁◯◯◯ **Very low**  (a, c, d, e) |
| **Memory function** | SMD 1.22 SD higher (0.15 higher to 2.28 higher) | 47 (1 RCT) | ⨁⨁◯◯ **Low**  (a, d) |

** The risk in the intervention group (and its 95% confidence interval) is based on the assumed risk in the comparison group and the relative effect of the intervention (and its 95% CI).*

**Explanations**

a. Most contributing trials were at unclear or high risk of bias, chiefly owing to unclear allocation concealment and unblinded outcome assessment (Cochrane Risk of Bias tool, version 1).

b. Substantial statistical heterogeneity together with incoherence between direct and indirect estimates in the network.

c. The 95% confidence interval is wide and crosses the null value (no effect).

d. Substantial statistical heterogeneity among the studies informing this estimate.

e. Funnel-plot asymmetry and a significant Egger test for executive function raised concern about reporting bias.

**Summary of findings: RE (remote exercise) compared to VR_EC (virtual reality exercise combined with cognitive training) for cognitive function in older adults aged ≥60 years**

| **Outcomes** | **Anticipated absolute effects* (95% CI)** | **№ of participants (studies)** | **Certainty of the evidence (GRADE)** |
| --- | --- | --- | --- |
| **Memory function** | SMD 1.54 SD higher (0.24 higher to 2.89 higher) | (0 RCTs) | ⨁⨁⨁◯ **Moderate**  (a) |

** The risk in the intervention group (and its 95% confidence interval) is based on the assumed risk in the comparison group and the relative effect of the intervention (and its 95% CI).*

**Explanations**

a. Most contributing trials were at unclear or high risk of bias, chiefly owing to unclear allocation concealment and unblinded outcome assessment. In addition, this comparison is a network estimate informed only by indirect evidence, with no direct head-to-head trials.

**Summary of findings: VR_EC (virtual reality exercise combined with cognitive training) compared to NI (non-intervention) for cognitive function in older adults aged ≥60 years**

| **Outcomes** | **Anticipated absolute effects* (95% CI)** | **№ of participants (studies)** | **Certainty of the evidence (GRADE)** |
| --- | --- | --- | --- |
| **Global cognition** | SMD 0.28 SD higher (0.14 lower to 0.73 higher) | 375 (4 RCTs) | ⨁◯◯◯ **Very low**  (a, b, c) |
| **Executive function** | SMD 0.42 SD higher (0.14 lower to 0.97 higher) | 358 (3 RCTs) | ⨁◯◯◯ **Very low**  (a, b, c, d) |
| **Memory function** | SMD 0.24 SD lower (1.01 lower to 0.48 higher) | 293 (1 RCT) | ⨁◯◯◯ **Very low**  (a, e) |

** The risk in the intervention group (and its 95% confidence interval) is based on the assumed risk in the comparison group and the relative effect of the intervention (and its 95% CI).*

**Explanations**

a. Most contributing trials were at unclear or high risk of bias, chiefly owing to unclear allocation concealment and unblinded outcome assessment (Cochrane Risk of Bias tool, version 1).

b. Substantial statistical heterogeneity among the studies informing this estimate.

c. The 95% confidence interval is wide and crosses the null value (no effect).

d. Funnel-plot asymmetry and a significant Egger test for executive function raised concern about reporting bias.

e. Evidence from a single small study with a very wide 95% confidence interval that crosses the null value.

**Summary of findings: VR_EC (virtual reality exercise combined with cognitive training) compared to RI (routine intervention) for cognitive function in older adults aged ≥60 years**

| **Outcomes** | **Anticipated absolute effects* (95% CI)** | **№ of participants (studies)** | **Certainty of the evidence (GRADE)** |
| --- | --- | --- | --- |
| **Global cognition** | SMD 0.08 SD higher (0.39 lower to 0.61 higher) | 17 (1 RCT) | ⨁◯◯◯ **Very low**  (a, b) |
| **Executive function** | SMD 0.25 SD higher (0.39 lower to 0.89 higher) | 20 (1 RCT) | ⨁◯◯◯ **Very low**  (a, c, d) |
| **Memory function** | SMD 0.32 SD lower (1.13 lower to 0.44 higher) | 20 (1 RCT) | ⨁◯◯◯ **Very low**  (a, b) |

** The risk in the intervention group (and its 95% confidence interval) is based on the assumed risk in the comparison group and the relative effect of the intervention (and its 95% CI).*

**Explanations**

a. Most contributing trials were at unclear or high risk of bias, chiefly owing to unclear allocation concealment and unblinded outcome assessment (Cochrane Risk of Bias tool, version 1).

b. Evidence from a single small study with a very wide 95% confidence interval that crosses the null value.

c. The 95% confidence interval is wide and crosses the null value (no effect).

d. Funnel-plot asymmetry and a significant Egger test for executive function raised concern about reporting bias.

**Table 2. Global cognition_GRADE_random_SMD_Report.**

| **Comparison** | **Number of studies** | **Within-study bias** | **Reporting bias** | **Indirectness** | **Imprecision** | **Heterogeneity** | **Incoherence** | **Confidence rating** | **Reason(s) for downgrading** |
| --- | --- | --- | --- | --- | --- | --- | --- | --- | --- |
| **IVR_E:RI** | **2** | **Some concerns** | **Low risk** | **No concerns** | **No concerns** | **Some concerns** | **Major concerns** | **Very low** | **["Within-study bias","Heterogeneity","Incoherence"]** |
| **NI:NI_ExG** | **6** | **Some concerns** | **Low risk** | **No concerns** | **No concerns** | **Some concerns** | **Major concerns** | **Very low** | **["Within-study bias","Heterogeneity","Incoherence"]** |
| **NI:RE** | **1** | **Some concerns** | **Low risk** | **No concerns** | **Some concerns** | **Some concerns** | **Some concerns** | **Very low** | **["Within-study bias","Imprecision","Heterogeneity","Incoherence"]** |
| **NI:RI** | **2** | **Some concerns** | **Low risk** | **No concerns** | **Some concerns** | **Some concerns** | **No concerns** | **Very low** | **["Within-study bias","Imprecision","Heterogeneity"]** |
| **NI:VR_EC** | **3** | **Some concerns** | **Low risk** | **No concerns** | **Some concerns** | **Some concerns** | **No concerns** | **Very low** | **["Within-study bias","Imprecision","Heterogeneity"]** |
| **NI_ExG:RI** | **14** | **Some concerns** | **Low risk** | **No concerns** | **No concerns** | **Some concerns** | **No concerns** | **Low** | **["Within-study bias","Heterogeneity"]** |
| **RE:RI** | **3** | **Some concerns** | **Low risk** | **No concerns** | **Some concerns** | **Some concerns** | **Some concerns** | **Very low** | **["Within-study bias","Imprecision","Heterogeneity","Incoherence"]** |
| **RI:VR_EC** | **1** | **Some concerns** | **Low risk** | **No concerns** | **Major concerns** | **No concerns** | **No concerns** | **Very low** | **["Within-study bias","Imprecision"]** |
| **IVR_E:NI** | **0** | **Some concerns** | **Low risk** | **No concerns** | **No concerns** | **Some concerns** | **Major concerns** | **Very low** | **["Within-study bias","Heterogeneity","Incoherence"]** |
| **IVR_E:NI_ExG** | **0** | **Some concerns** | **Low risk** | **No concerns** | **Major concerns** | **No concerns** | **Major concerns** | **Very low** | **["Within-study bias","Imprecision","Incoherence"]** |
| **IVR_E:RE** | **0** | **Some concerns** | **Low risk** | **No concerns** | **Major concerns** | **No concerns** | **Major concerns** | **Very low** | **["Within-study bias","Imprecision","Incoherence"]** |
| **IVR_E:VR_EC** | **0** | **Some concerns** | **Low risk** | **No concerns** | **Major concerns** | **No concerns** | **Major concerns** | **Very low** | **["Within-study bias","Imprecision","Incoherence"]** |
| **NI_ExG:RE** | **0** | **Some concerns** | **Low risk** | **No concerns** | **Some concerns** | **Some concerns** | **Major concerns** | **Very low** | **["Within-study bias","Imprecision","Heterogeneity","Incoherence"]** |
| **NI_ExG:VR_EC** | **0** | **Some concerns** | **Low risk** | **No concerns** | **Major concerns** | **No concerns** | **Major concerns** | **Very low** | **["Within-study bias","Imprecision","Incoherence"]** |
| **RE:VR_EC** | **0** | **Some concerns** | **Low risk** | **No concerns** | **Major concerns** | **No concerns** | **Major concerns** | **Very low** | **["Within-study bias","Imprecision","Incoherence"]** |

**Fig. 1.** Risk of bias for global cognition (contribution of each study to the network)

**Table 3. Executive function_GRADE_random_SMD_Report.**

| **Comparison** | **Number of studies** | **Within-study bias** | **Reporting bias** | **Indirectness** | **Imprecision** | **Heterogeneity** | **Incoherence** | **Confidence rating** | **Reason(s) for downgrading** |
| --- | --- | --- | --- | --- | --- | --- | --- | --- | --- |
| **IVR_E:NI_ExG** | **1** | **Some concerns** | **Some concerns** | **No concerns** | **Major concerns** | **No concerns** | **No concerns** | **Very low** | **["Within-study bias","Reporting bias","Imprecision"]** |
| **IVR_E:RI** | **4** | **Some concerns** | **Some concerns** | **No concerns** | **Major concerns** | **No concerns** | **No concerns** | **Very low** | **["Within-study bias","Reporting bias","Imprecision"]** |
| **NI:NI_ExG** | **7** | **Some concerns** | **Some concerns** | **No concerns** | **No concerns** | **Major concerns** | **No concerns** | **Very low** | **["Within-study bias","Reporting bias","Heterogeneity"]** |
| **NI:RE** | **1** | **Some concerns** | **Some concerns** | **No concerns** | **Some concerns** | **Some concerns** | **No concerns** | **Very low** | **["Within-study bias","Reporting bias","Imprecision","Heterogeneity"]** |
| **NI:RI** | **2** | **Some concerns** | **Some concerns** | **No concerns** | **Some concerns** | **Some concerns** | **No concerns** | **Very low** | **["Within-study bias","Reporting bias","Imprecision","Heterogeneity"]** |
| **NI:VR_EC** | **3** | **Some concerns** | **Some concerns** | **No concerns** | **Some concerns** | **Some concerns** | **No concerns** | **Very low** | **["Within-study bias","Reporting bias","Imprecision","Heterogeneity"]** |
| **NI_ExG:RI** | **13** | **Some concerns** | **Some concerns** | **No concerns** | **Some concerns** | **Some concerns** | **No concerns** | **Very low** | **["Within-study bias","Reporting bias","Imprecision","Heterogeneity"]** |
| **RE:RI** | **4** | **Some concerns** | **Some concerns** | **No concerns** | **Some concerns** | **Some concerns** | **No concerns** | **Very low** | **["Within-study bias","Reporting bias","Imprecision","Heterogeneity"]** |
| **RI:VR_EC** | **1** | **Some concerns** | **Some concerns** | **No concerns** | **Major concerns** | **No concerns** | **No concerns** | **Very low** | **["Within-study bias","Reporting bias","Imprecision"]** |
| **IVR_E:NI** | **0** | **Some concerns** | **Some concerns** | **No concerns** | **Some concerns** | **Some concerns** | **Major concerns** | **Very low** | **["Within-study bias","Reporting bias","Imprecision","Heterogeneity","Incoherence"]** |
| **IVR_E:RE** | **0** | **Some concerns** | **Some concerns** | **No concerns** | **Major concerns** | **No concerns** | **Major concerns** | **Very low** | **["Within-study bias","Reporting bias","Imprecision","Incoherence"]** |
| **IVR_E:VR_EC** | **0** | **Some concerns** | **Some concerns** | **No concerns** | **Major concerns** | **No concerns** | **Major concerns** | **Very low** | **["Within-study bias","Reporting bias","Imprecision","Incoherence"]** |
| **NI_ExG:RE** | **0** | **Some concerns** | **Some concerns** | **No concerns** | **Major concerns** | **No concerns** | **Major concerns** | **Very low** | **["Within-study bias","Reporting bias","Imprecision","Incoherence"]** |
| **NI_ExG:VR_EC** | **0** | **Some concerns** | **Some concerns** | **No concerns** | **Major concerns** | **No concerns** | **Major concerns** | **Very low** | **["Within-study bias","Reporting bias","Imprecision","Incoherence"]** |
| **RE:VR_EC** | **0** | **Some concerns** | **Some concerns** | **No concerns** | **Major concerns** | **No concerns** | **Major concerns** | **Very low** | **["Within-study bias","Reporting bias","Imprecision","Incoherence"]** |

**Fig. 2.** Risk of bias for executive function (contribution of each study to the network)

**Table 4. Memory function_GRADE_random_SMD_Report.**

| **Comparison** | **Number of studies** | **Within-study bias** | **Reporting bias** | **Indirectness** | **Imprecision** | **Heterogeneity** | **Incoherence** | **Confidence rating** | **Reason(s) for downgrading** |
| --- | --- | --- | --- | --- | --- | --- | --- | --- | --- |
| **IVR_E:NI_ExG** | **1** | **Some concerns** | **Low risk** | **No concerns** | **Major concerns** | **No concerns** | **No concerns** | **Very low** | **["Within-study bias","Imprecision"]** |
| **IVR_E:RI** | **2** | **Some concerns** | **Low risk** | **No concerns** | **Some concerns** | **Some concerns** | **No concerns** | **Very low** | **["Within-study bias","Imprecision","Heterogeneity"]** |
| **NI:NI_ExG** | **7** | **Some concerns** | **Low risk** | **No concerns** | **Some concerns** | **Some concerns** | **No concerns** | **Very low** | **["Within-study bias","Imprecision","Heterogeneity"]** |
| **NI:RI** | **3** | **Some concerns** | **Low risk** | **No concerns** | **Major concerns** | **No concerns** | **No concerns** | **Very low** | **["Within-study bias","Imprecision"]** |
| **NI:VR_EC** | **1** | **Some concerns** | **Low risk** | **No concerns** | **Major concerns** | **No concerns** | **No concerns** | **Very low** | **["Within-study bias","Imprecision"]** |
| **NI_ExG:RI** | **9** | **Some concerns** | **Low risk** | **No concerns** | **Some concerns** | **Some concerns** | **No concerns** | **Very low** | **["Within-study bias","Imprecision","Heterogeneity"]** |
| **RE:RI** | **1** | **Some concerns** | **Low risk** | **No concerns** | **No concerns** | **Some concerns** | **No concerns** | **Low** | **["Within-study bias","Heterogeneity"]** |
| **RI:VR_EC** | **1** | **Some concerns** | **Low risk** | **No concerns** | **Major concerns** | **No concerns** | **No concerns** | **Very low** | **["Within-study bias","Imprecision"]** |
| **IVR_E:NI** | **0** | **Some concerns** | **Low risk** | **No concerns** | **Some concerns** | **Some concerns** | **No concerns** | **Very low** | **["Within-study bias","Imprecision","Heterogeneity"]** |
| **IVR_E:RE** | **0** | **Some concerns** | **Low risk** | **No concerns** | **Major concerns** | **No concerns** | **No concerns** | **Very low** | **["Within-study bias","Imprecision"]** |
| **IVR_E:VR_EC** | **0** | **Some concerns** | **Low risk** | **No concerns** | **Some concerns** | **Some concerns** | **No concerns** | **Very low** | **["Within-study bias","Imprecision","Heterogeneity"]** |
| **NI:RE** | **0** | **Some concerns** | **Low risk** | **No concerns** | **No concerns** | **Some concerns** | **No concerns** | **Low** | **["Within-study bias","Heterogeneity"]** |
| **NI_ExG:RE** | **0** | **Some concerns** | **Low risk** | **No concerns** | **No concerns** | **Major concerns** | **No concerns** | **Very low** | **["Within-study bias"]** |
| **NI_ExG:VR_EC** | **0** | **Some concerns** | **Low risk** | **No concerns** | **Some concerns** | **Some concerns** | **No concerns** | **Very low** | **["Within-study bias","Imprecision","Heterogeneity"]** |
| **RE:VR_EC** | **0** | **Some concerns** | **Low risk** | **No concerns** | **No concerns** | **No concerns** | **No concerns** | **Moderate** | **["Within-study bias"]** |

**Fig. 3.** Risk of bias for memory function (contribution of each study to the network)
