# Supplementary material for: Digital Physical Exercise Interventions for Cognitive Functions in Older Adults: Systematic Review and Bayesian Network Meta-Analysis of Randomized Controlled Trials
Source: J Med Internet Res. 2026 Aug 18;28:e92764. doi: 10.2196/92764 (PMC13485154; doi:10.2196/92764)
Supplement: Supplementary file 10 [file jmir-v28-e92764-s009.docx]

| **Section and Topic** | **Item #** | **Checklist item** | **Location where item is reported** |
| --- | --- | --- | --- |
| **TITLE** | | |  |
| Title | 1 | Identify the report as a systematic review. | Title page — the title explicitly includes 'Systematic Review and Bayesian Network Meta-Analysis' |
| **ABSTRACT** | | |  |
| Abstract | 2 | See the PRISMA 2020 for Abstracts checklist. | Abstract section (see separate PRISMA 2020 for Abstracts checklist) |
| **INTRODUCTION** | | |  |
| Rationale | 3 | Describe the rationale for the review in the context of existing knowledge. | Introduction, Rationale subsection — describes cognitive decline burden, limitations of prior single-modality reviews (refs [26–31,35,36]), and the gap in multi-modal comparisons across cognitive domains |
| Objectives | 4 | Provide an explicit statement of the objective(s) or question(s) the review addresses. | Introduction, Objectives subsection — 'to systematically compare and rank the effectiveness of four digital physical exercise interventions (IVR_E, NI_ExG, RE, VR_EC) against RI or NI on global cognition, executive function, and memory function in older adults aged ≥60 years' |
| **METHODS** | | |  |
| Eligibility criteria | 5 | Specify the inclusion and exclusion criteria for the review and how studies were grouped for the syntheses. | Methods, Eligibility Criteria section — Type of Studies (peer-reviewed RCTs), Participants (≥60 years), Interventions (four digital modalities), Controls (RI or NI), Outcomes (untrained cognitive outcomes); exclusion criteria stated. Grouping described under Data Items, Other Variables |
| Information sources | 6 | Specify all databases, registers, websites, organisations, reference lists and other sources searched or consulted to identify studies. Specify the date when each source was last searched or consulted. | Methods, Information Sources — PubMed, Embase, Web of Science, CENTRAL, PsycINFO, CINAHL (initial search 29 April 2025; updated 15 April 2026); ClinicalTrials.gov and ICTRP; reference lists of included studies and prior reviews |
| Search strategy | 7 | Present the full search strategies for all databases, registers and websites, including any filters and limits used. | Methods, Search Strategy — concept blocks, controlled vocabulary (MeSH, EMTREE, APA Thesaurus, CINAHL Headings), Boolean operators, validated RCT filters; full strategies in Multimedia Appendix 1 |
| Selection process | 8 | Specify the methods used to decide whether a study met the inclusion criteria of the review, including how many reviewers screened each record and each report retrieved, whether they worked independently, and if applicable, details of automation tools used in the process. | Methods, Selection Process — two independent reviewers screened titles/abstracts and full texts; disagreements resolved by discussion and third reviewer; no automation tools; duplicates removed using EndNote X21 |
| Data collection process | 9 | Specify the methods used to collect data from reports, including how many reviewers collected data from each report, whether they worked independently, any processes for obtaining or confirming data from study investigators, and if applicable, details of automation tools used in the process. | Methods, Data Collection Process — one reviewer extracted data; second reviewer independently verified; disagreements resolved by discussion/third reviewer; no automation tools; no contact with investigators; handling of multiple reports described |
| Data items | 10a | List and define all outcomes for which data were sought. Specify whether all results that were compatible with each outcome domain in each study were sought (e.g. for all measures, time points, analyses), and if not, the methods used to decide which results to collect. | Methods, Data Items, Outcomes — three domains defined: global cognition (composite instruments, e.g., MMSE, MoCA), executive function (control/flexibility/inhibition, e.g., TMT-B, Stroop), memory function (encoding/storage/retrieval, e.g., digit span, verbal learning); all compatible results sought; most commonly used instrument selected when multiple reported; Multimedia Appendix 2 lists all tools |
|  | 10b | List and define all other variables for which data were sought (e.g. participant and intervention characteristics, funding sources). Describe any assumptions made about any missing or unclear information. | Methods, Data Items, Other Variables — author, year, country, sample size, mean age, sex ratio, cognitive status, intervention type/duration/frequency/dose/delivery mode; missing information treated as missing; no assumptions made |
| Study risk of bias assessment | 11 | Specify the methods used to assess risk of bias in the included studies, including details of the tool(s) used, how many reviewers assessed each study and whether they worked independently, and if applicable, details of automation tools used in the process. | Methods, Study Risk of Bias Assessment — Cochrane Risk of Bias tool version 1; two independent reviewers; six domains assessed (random sequence generation, allocation concealment, blinding of outcome assessors, incomplete data, selective reporting, other bias); worst-domain rule; third reviewer for disagreements; no automation tools |
| Effect measures | 12 | Specify for each outcome the effect measure(s) (e.g. risk ratio, mean difference) used in the synthesis or presentation of results. | Methods, Effect Measures — standardized mean difference (SMD) and 95% CI for all three continuous cognitive outcomes; effect sizes classified as small (<0.40), moderate (0.40–0.70), or large (>0.70) per Cochrane Handbook |
| Synthesis methods | 13a | Describe the processes used to decide which studies were eligible for each synthesis (e.g. tabulating the study intervention characteristics and comparing against the planned groups for each synthesis (item #5)). | Methods, Synthesis Methods — studies eligible if sufficient data to calculate SMD and SE for ≥1 cognitive domain; required to form a connected network; eligibility for each synthesis determined by data availability and network connectivity |
|  | 13b | Describe any methods required to prepare the data for presentation or synthesis, such as handling of missing summary statistics, or data conversions. | Methods, Synthesis Methods and Data Collection Process — means and SDs calculated from medians/95% CIs using Cochrane Handbook conversion methods [44] where not directly reported |
|  | 13c | Describe any methods used to tabulate or visually display results of individual studies and syntheses. | Methods, Synthesis Methods — forest plots (pairwise meta-analyses), network plots (network geometry), league tables (all NMA pairwise comparisons), SUCRA plots; presented in Results figures and tables |
|  | 13d | Describe any methods used to synthesize results and provide a rationale for the choice(s). If meta-analysis was performed, describe the model(s), method(s) to identify the presence and extent of statistical heterogeneity, and software package(s) used. | Methods, Synthesis Methods — Bayesian NMA using gemtc package v1.0-2 in R (v4.4.2); random-effects model; four independent MCMC chains, 15,000 iterations, 5,000 burn-in; SUCRA for ranking [50]; PIs via netmeta package using Noma 2023 [56]; consistency assessed by node-splitting; pairwise meta-analyses in STATA 15.0; I² for heterogeneity (thresholds 25%/50%/75%) |
|  | 13e | Describe any methods used to explore possible causes of heterogeneity among study results (e.g. subgroup analysis, meta-regression). | Methods, Synthesis Methods — pre-specified subgroup analyses by cognitive status (healthy vs. impaired), intervention type, and total training dose (high >1000 min vs. low ≤1000 min); statistical test for interaction between subgroups; detailed in Multimedia Appendix 6 |
|  | 13f | Describe any sensitivity analyses conducted to assess robustness of the synthesized results. | Methods, Synthesis Methods — leave-one-out sensitivity analyses (each study systematically omitted); trim-and-fill for publication bias; results in Multimedia Appendix 6 |
| Reporting bias assessment | 14 | Describe any methods used to assess risk of bias due to missing results in a synthesis (arising from reporting biases). | Methods, Reporting Bias Assessment — comparison-adjusted funnel plots and Egger test for each cognitive outcome; two independent reviewers; Multimedia Appendix 7 |
| Certainty assessment | 15 | Describe any methods used to assess certainty (or confidence) in the body of evidence for an outcome. | Methods, Certainty Assessment — CINeMA framework [58,59] (GRADE adaptation for NMA); six domains: within-study bias, reporting bias, indirectness, imprecision, heterogeneity, incoherence; rated as no/some/major concerns; overall confidence: high/moderate/low/very low; two independent reviewers; CINeMA web application (https://cinema.ispm.unibe.ch); full assessments in Multimedia Appendix 8 |
| **RESULTS** | | |  |
| Study selection | 16a | Describe the results of the search and selection process, from the number of records identified in the search to the number of studies included in the review, ideally using a flow diagram. | Results, Study Selection, Flow of Studies — 30,454 database records + 239 registry records; 15,872 duplicates removed; 14,582 screened; 936 full-texts assessed; 51 unique studies included; Figure 1 (PRISMA flow diagram) |
|  | 16b | Cite studies that might appear to meet the inclusion criteria, but which were excluded, and explain why they were excluded. | Results, Study Selection, Excluded Studies — five examples cited with reasons (quasi-RCT, age <60, no physical component, cognitive training only, non-randomized); complete list of 39 excluded studies with reasons in Multimedia Appendix 3 |
| Study characteristics | 17 | Cite each included study and present its characteristics. | Results, Study Characteristics — 51 studies (3,673 participants); cognitive status, intervention type, study count, and participant counts summarized; all studies cited [15–25, 32–34, 61–99]; detailed characteristics in Multimedia Appendix 4 |
| Risk of bias in studies | 18 | Present assessments of risk of bias for each included study. | Results, Risk of Bias in Studies — domain-level and overall judgments for all 51 studies; Figure 2 (risk of bias diagram); 8 low risk, 30 some concerns, 13 high risk overall |
| Results of individual studies | 19 | For all outcomes, present, for each study: (a) summary statistics for each group (where appropriate) and (b) an effect estimate and its precision (e.g. confidence/credible interval), ideally using structured tables or plots. | Results, Results of Individual Studies — SMDs and 95% CIs for each study per outcome; Figure 3 (forest plots A–C); overall pooled SMD with 95% CI and PI reported for each domain |
| Results of syntheses | 20a | For each synthesis, briefly summarise the characteristics and risk of bias among contributing studies. | Results, Results of Syntheses, Pairwise Meta-Analysis — characteristics and risk of bias patterns described for each comparison; contributing study counts (k) reported in Table 1; heterogeneity statistics in Table 2 |
|  | 20b | Present results of all statistical syntheses conducted. If meta-analysis was done, present for each the summary estimate and its precision (e.g. confidence/credible interval) and measures of statistical heterogeneity. If comparing groups, describe the direction of the effect. | Results, Results of Syntheses — pairwise meta-analysis results (Table 1); NMA results including league table (Table 3), SUCRA rankings, and PIs; network plots (Figure 3); consistency test results (Multimedia Appendix 5) |
|  | 20c | Present results of all investigations of possible causes of heterogeneity among study results. | Results, Heterogeneity Investigations — subgroup analyses by cognitive status, intervention type, and training dose; I² and p-values summarized in Table 2; detailed results in Multimedia Appendix 6 |
|  | 20d | Present results of all sensitivity analyses conducted to assess the robustness of the synthesized results. | Results, Sensitivity Analyses — leave-one-out results stable (range ~0.20–0.52 for global cognition, 0.07–0.42 for executive function, 0.02–0.53 for memory); no single study shifted estimate direction; Multimedia Appendix 6 |
| Reporting biases | 21 | Present assessments of risk of bias due to missing results (arising from reporting biases) for each synthesis assessed. | Results, Reporting Biases — funnel plots and Egger test results for all three outcomes; no significant asymmetry for global cognition (p = 0.12) or memory (p = 0.68); significant asymmetry for executive function (p = 0.005) with trim-and-fill estimating 0 missing studies; Multimedia Appendix 7 |
| Certainty of evidence | 22 | Present assessments of certainty (or confidence) in the body of evidence for each outcome assessed. | Results, Certainty of Evidence — CINeMA ratings for all 45 pairwise comparisons (15 per outcome): most rated very low; one low for global cognition (NI_ExG vs. RI); one moderate for memory (RE vs. VR_EC); dominant downgrading factors: within-study bias (all outcomes), heterogeneity, incoherence; Multimedia Appendix 8 |
| **DISCUSSION** | | |  |
| Discussion | 23a | Provide a general interpretation of the results in the context of other evidence. | Discussion — Principal Findings paragraph; detailed paragraphs on global cognition, executive function, and memory function; Implications section — interpreted in context of prior exergame reviews [28–31], traditional exercise NMAs [35,36], and mechanistic evidence [72,76,101,105,106] |
|  | 23b | Discuss any limitations of the evidence included in the review. | Discussion, Limitations of the Evidence — very low CINeMA certainty; within-study bias; substantial heterogeneity in NI_ExG and RE; sparse IVR_E network; RE memory advantage from single direct comparison; short- to medium-term follow-up only |
|  | 23c | Discuss any limitations of the review processes used. | Discussion, Limitations of the Review Processes — English-language restriction; no PRESS peer review of search; no author contact; no grey literature; possible residual reporting bias for executive function; broad modality grouping; meta-regression underpowered; reliance on indirect evidence in memory domain |
|  | 23d | Discuss implications of the results for practice, policy, and future research. | Discussion, Implications; Conclusions — clinical: domain-tailored modality selection; public health: scalability of RE/NI_ExG for rural settings; policy: evidence base for digital health investment; future research: multi-site RCTs, IPD dose–response analyses, mechanistic studies |
| **OTHER INFORMATION** | | |  |
| Registration and protocol | 24a | Provide registration information for the review, including register name and registration number, or state that the review was not registered. | Other Information, Registration and Protocol; Abstract (Trial Registration); Ethical Considerations — PROSPERO CRD420251030142 |
|  | 24b | Indicate where the review protocol can be accessed, or state that a protocol was not prepared. | Other Information, Registration and Protocol — 'The review protocol is publicly accessible via the PROSPERO registry. |
|  | 24c | Describe and explain any amendments to information provided at registration or in the protocol. | Other Information, Registration and Protocol — three amendments listed: (1) search updated to April 2026; (2) addition of prediction intervals (Noma 2023 method); (3) addition of CINeMA certainty grading |
| Support | 25 | Describe sources of financial or non-financial support for the review, and the role of the funders or sponsors in the review. | Other Information, Support — National Natural Science Foundation of China (grant numbers: 32371132 and 32571260); funders had no role in design, data collection, analysis, interpretation, or publication decision |
| Competing interests | 26 | Declare any competing interests of review authors. | Other Information, Competing Interests — 'All authors declare that they have no competing interests’. |
| Availability of data, code and other materials | 27 | Report which of the following are publicly available and where they can be found: template data collection forms; data extracted from included studies; data used for all analyses; analytic code; any other materials used in the review. | Other Information, Availability of Data, Code, and Other Materials — OSF repository (https://osf.io/6q7tu/): data extraction form template, coded study-level data from all included RCTs, all R scripts for Bayesian NMA; view-only link provided |

*From:*  Page MJ, McKenzie JE, Bossuyt PM, Boutron I, Hoffmann TC, Mulrow CD, et al. The PRISMA 2020 statement: an updated guideline for reporting systematic reviews. BMJ 2021;372:n71. doi: 10.1136/bmj.n71
