# Supplementary material for: Digital Physical Exercise Interventions for Cognitive Functions in Older Adults: Systematic Review and Bayesian Network Meta-Analysis of Randomized Controlled Trials
Source: J Med Internet Res. 2026 Aug 18;28:e92764. doi: 10.2196/92764 (PMC13485154; doi:10.2196/92764)
Supplement: Supplementary file 12 [file jmir-v28-e92764-s012.docx]

**PRISMA-S Checklist**

| **Section/topic** | **#** | **Checklist item** | | **Location(s) Reported** |
| --- | --- | --- | --- | --- |
| **INFORMATION SOURCES AND METHODS** | | | | |
| Database name | 1 | Name each individual database searched, stating the platform for each. | | Methods, Information Sources — PubMed (MEDLINE, via PubMed.gov); Embase (via Embase.com); Web of Science Core Collection (SCI-EXPANDED and SSCI); CENTRAL (Cochrane Library); PsycINFO (via EBSCOhost); CINAHL Plus with Full Text (via EBSCOhost). Each database was searched individually; no multi-database searching on a single platform was performed |
| Multi-database searching | 2 | If databases were searched simultaneously on a single platform, state the name of the platform, listing all of the databases searched. | | Methods, Information Sources — Not applicable: each database was searched individually on its own platform; no simultaneous multi-database searching was performed |
| Study registries | 3 | List any study registries searched. | | Methods, Information Sources — ClinicalTrials.gov and the WHO International Clinical Trials Registry Platform (ICTRP) |
| Online resources and browsing | 4 | Describe any online or print source purposefully searched or browsed (e.g., tables of contents, print conference proceedings, web sites), and how this was done. | | Methods, Information Sources — 'No additional online sources, conference proceedings, or grey literature repositories were purposefully browsed' |
| Citation searching | 5 | Indicate whether cited references or citing references were examined, and describe any methods used for locating cited/citing references (e.g., browsing reference lists, using a citation index, setting up email alerts for references citing included studies). | | Methods, Information Sources — 'We also performed a manual search of the reference lists of all included studies and relevant previous reviews to identify additional eligible studies'; 18 additional records identified; 3 met eligibility |
| Contacts | 6 | Indicate whether additional studies or data were sought by contacting authors, experts, manufacturers, or others. | | Methods, Information Sources — 'We did not contact study authors or experts to obtain additional data or unpublished studies' |
| Other methods | 7 | Describe any additional information sources or search methods used. | | Methods, Information Sources — 'No other supplementary search methods were used.' No natural language processing tools, no automated translation, no text frequency analysis tools were used |
| **SEARCH STRATEGIES** | | | | |
| Full search strategies | 8 | Include the search strategies for each database and information source, copied and pasted exactly as run. | | Methods, Search Strategy — 'The complete search strategies for all databases, copied and pasted exactly as run, are presented in Multimedia Appendix 1' |
| Limits and restrictions | 9 | Specify that no limits were used, or describe any limits or restrictions applied to a search (e.g., date or time period, language, study design) and provide justification for their use. | | Methods, Search Strategy — Date restriction: January 1, 2010–April 1, 2026 (justified: digital physical exercise interventions for older adults emerged as distinct research field around 2010). Language restriction: English-language only in five databases (due to resource constraints for translation); no language restriction applied in CENTRAL to minimize language bias |
| Search filters | 10 | Indicate whether published search filters were used (as originally designed or modified), and if so, cite the filter(s) used. | | Methods, Search Strategy — Cochrane Highly Sensitive Search Strategy for RCTs (PubMed) [41]; Cochrane Embase RCT filter 2023 revision (Embase) [42]; Cochrane CINAHL filter (CINAHL) [43]; adapted filter (PsycINFO); no design filter applied to CENTRAL |
| Prior work | 11 | Indicate when search strategies from other literature reviews were adapted or reused for a substantive part or all of the search, citing the previous review(s). | | Methods, Search Strategy — 'The search strategies were developed independently for this review and were not adapted from prior published reviews' |
| Updates | 12 | Report the methods used to update the search(es) (e.g., rerunning searches, email alerts). | | Methods, Information Sources; Other Information, Registration and Protocol — 'the database search was updated on April 15, 2026, by rerunning the identical search strategies across all six databases to extend coverage to April 1, 2026' |
| Dates of searches | 13 | For each search strategy, provide the date when the last search occurred. | | Methods, Information Sources — Initial search: April 29, 2025; Updated search: April 15, 2026; both covering January 1, 2010–April 1, 2026. Also reported in Abstract Methods |
| **PEER REVIEW** | | | | |
| Peer review | 14 | Describe any search peer review process. | | Methods, Search Strategy — 'The search strategies were reviewed by all members of the review team, but a formal peer review process using a validated instrument such as Peer Review of Electronic Search Strategies (PRESS) was not conducted' |
| **MANAGING RECORDS** | | | | |
| Total Records | 15 | Document the total number of records identified from each database and other information sources. | | Methods, Search Strategy; Results, Study Selection — 'The searches yielded a total of 30,454 records across all six databases (PubMed: 6,842; Embase: 9,517; Web of Science: 5,963; CENTRAL: 3,286; PsycINFO: 2,714; CINAHL: 1,893), plus 239 records from trial registries'; Figure 1 (PRISMA flow diagram) |
| Deduplication | 16 | Describe the processes and any software used to deduplicate records from multiple database searches and other information sources. | | Methods, Selection Process — 'Duplicate records were removed using EndNote X21 reference management software, followed by manual verification'; 15,872 duplicates removed from 30,693 total records |
|  |  |  | |  |
| PRISMA-S: An Extension to the PRISMA Statement for Reporting Literature Searches in Systematic Reviews | | | |  |
| Rethlefsen ML, Kirtley S, Waffenschmidt S, Ayala AP, Moher D, Page MJ, Koffel JB, PRISMA-S Group. | | | |  |
| Last updated February 27, 2020. | | |  |  |
